# Integration of oral health promotion in primary school health programs: A scoping review

**DOI:** 10.1371/journal.pgph.0007390

**Published:** 2026-10-05

**Authors:** Nikita Surani, Nathalia Carolina Fernandes Fagundes, Harmanpreet Kaur, Rebecca Gokiert, Arnaldo Perez-Garcia, Maryam Amin

**Affiliations:** 1 Mike Petryk School of Dentistry, Faculty of Medicine and Dentistry, University of Alberta, Edmonton, Alberta, Canada; 2 School of Public Health, College of Health Sciences, University of Alberta, Edmonton, Alberta, Canada; PLOS: Public Library of Science, UNITED STATES OF AMERICA

## Abstract

Children experience significant oral health problems, compromising learning and quality of life. Schools are effective in promoting children’s overall health, within which oral health can be integrated. This scoping review aimed to map integration of oral health within primary school health programs. The review was guided by Arksey and O’Malley’s framework and adhered to Preferred Reporting Items for Systematic reviews and Meta-Analyses extensions for Scoping Reviews (PRISMA-ScR). JBI Manual for Evidence Synthesis was referred to for data extraction. A systematic search was performed across six databases with no restrictions on dates and locations. The review included 31 articles published between 1981 and 2025. Over 70% studies were concentrated exclusively in high-income and upper-middle income regions. Quantitative methods were reported in twenty-one studies. All studies reported programs targeting children. Only 12 included parents or guardians, mostly as passive recipients. Most common intervention delivery agents were teachers, identified in 25 studies. Unhealthy diet and poor hygiene were frequently targeted common risk factors, identified in 18 and 11 studies respectively. Tobacco use, stress, and injury were largely overlooked. Twenty-seven studies reported activity-based learning to promote general health. Classroom education, followed by interest holder engagement and structured toothbrushing were used to promote oral health, focusing on student skill-building and empowerment, alongside teacher involvement. None of the studies applied integration-specific or implementation science frameworks or models. Twenty-six studies assessed child-level oral health outcomes. In contrast, only seven reported changes in knowledge, attitudes, and practices among educators and parents. Ten reported program-related outcomes. Structured programs engaging children, targeting risk factors related to hygiene and diet alongside interest holder engagement and curriculum integration hold promise. This review highlights the need for theoretically informed, comprehensive, policy-focused, multi-level, multi-risk factor approaches to support sustainable integration of oral health into school health programs.

## Introduction

Dental caries among children is a public health problem with high burden in developing countries [1,2]. It can cause dental pain, leading to school absenteeism and poor learning outcomes [3]. Poor oral health among children is associated with suboptimal academic performance, compromised growth and development, as well as reduced quality of life [4–6]. Early oral infections are also associated with increased risk of other chronic diseases [7]. Beyond untreated decay leading to tooth loss, traumatic dental injuries are an emerging cause of compromised oral health in children [8].

The World Health Organization recognizes schools as ideal settings to promote and improve children’s oral health [9]. Activities part of school routine tend to have positive effects on children’s oral hygiene [10]. School interventions combining service access, skills-based education, teacher training, and parental engagement have the potential to improve children’s oral health outcomes [11]. Such combined interventions have yielded significantly lower Decayed Missing Filled Teeth (DMFT), Decayed Missing Filled Surfaces (DMFS), risk of DMFT, and plaque scores [11].

Dental screenings, toothpaste and toothbrush distribution, brushing demonstrations, dental sealant application, and fluoride treatments have been the main focus of oral health promotion in most schools [12]. Although important, these isolated activities can lead to only short-term results, such as a reduction in children’s plaque and gingival scores, with no significant change in mean caries status [13]. Furthermore, many such interventions have limited sustainability, given their high costs and resource requirements [14] – a limitation that can be addressed through integrated program models [15].

Most literature on integration is limited to health service delivery [16,17], with no known universal definition [18] applicable to public health programs. Based on existing literature, ‘integration’ can be understood as ‘merging into a single entity’ [19], ‘making of a unified whole [16], combining different disciplines [20], through actions or activities within and between organizations facilitated through coordination and collaboration [16,19] at several levels’ [17,21].

A promising strategy for integrating oral health within school health programs is the Common Risk Factor [CRF] approach [15] [Fig 1], which targets shared behavioural and social determinants of oral and other non-communicable diseases [22,24]. By addressing unhealthy diets, tobacco and alcohol use, injuries, poor hygiene, and overlapping social determinants, integrated interventions may be cost-effective, sustainable, and equity-oriented. Many existing school health programs already address some common risk factors [25–32], creating natural platform to embed oral health promotion within established initiatives [10]. However, despite endorsements of this approach from global health organizations, oral health is seldom embedded in existing programs [33].

**Fig 1:**
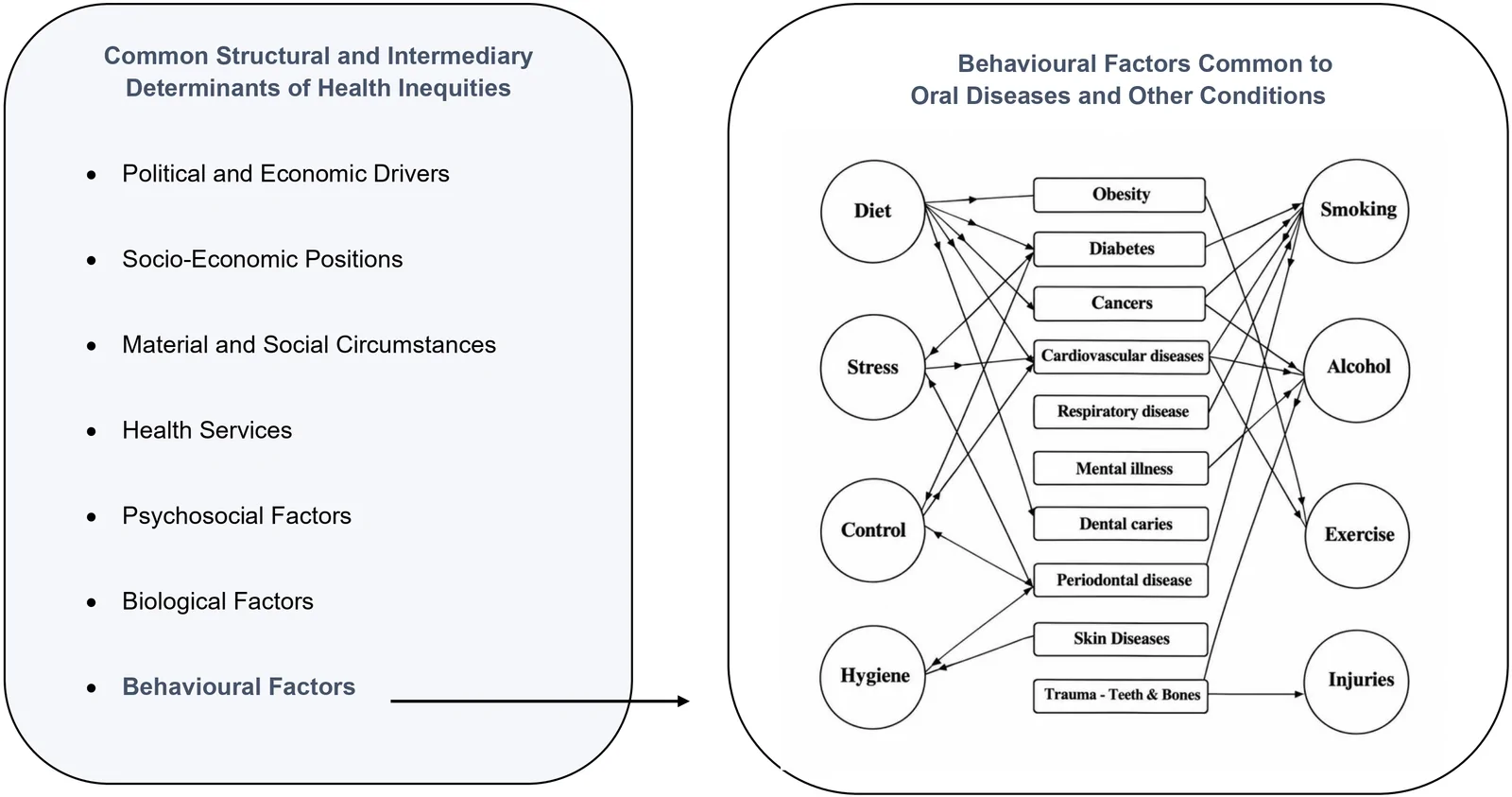
Common risk factor approach within social determinants framework for health inequities [22,23].

Drawing on these concepts, our review defines ‘integration of oral health into school health’ as ‘explicit or implicit incorporation of oral health-related activities into existing school health programs, by addressing shared risk factors common to oral diseases and other health conditions, to create connections and unification between oral health and other health promotion activities’. Such integration may be achieved through educational activities, preventive services, capacity building, skill development, curriculum-based initiatives, policy interventions, or other coordinated program components addressing shared risk factors. Accordingly, this review included studies if they explicitly or implicitly demonstrated this integration through strategies involving student skill building, curriculum restructuring, school policy changes, teacher and family involvement, professional engagement, resource access, or other coordinated whole school approaches rather than delivering oral health as a standalone intervention without any general health component(s).

Using established platforms such as schools allows delivery of integrated health promotion programs [34] that are acceptable, feasible [33], and lead to improved outcomes [35,36] – while reducing the need for additional resources, ensuring accessibility, continuity, and sustainability [37].

In addition to the CRF approach, several frameworks of integration exist explaining how health interventions can be coordinated across domains [38,39] through shared resources, structures, policies, norms, and collaboration [16]. However, little is known about how these frameworks can be operationalized in school health practice. Integrating oral health in school health programs has several benefits. However, its implementation is also not well articulated in literature [40] with little evidence on challenges of this approach [41].

To the best of our knowledge, no prior research has examined how oral health is integrated within school health programs. This gap is significant given that school-based health promotion has been widely recognized as an effective and equitable approach for addressing diverse health needs [42,43]. Oral health remains underrepresented and siloed from broader health agendas despite its shared determinants with other chronic conditions [24,44].

This review thus aimed to understand how oral health is incorporated within existing primary school health programs across different contexts, with a focus on program contents, components, and implementation approaches.

## Methods

A scoping review methodology was used because the review aimed to comprehensively map and summarize the breadth and nature of literature [45,46] on primary school health promotion programs with an oral health component, including understanding implementation strategies used and identify knowledge gaps to guide future research and programs. Consistent with established methodological guidance for scoping reviews, a risk of bias assessment was not conducted [46].

The review was guided by Arksey and O’Malley’s framework [45]. Data extraction and synthesis adhered to Joanna Briggs Institute [JBI] Manual for Evidence Synthesis [47]. Results were reported using Preferred Reporting Items for Systematic Reviews and Meta-Analyses extensions for scoping reviews [PRISMA-ScR] [48–50]. S1 Appendix shows the completed PRISMA checklist.

Four research questions guided this review and included:

1. How is oral health integrated within existing primary school health promotion programs [to understand program characteristics such as population targeted, duration, and content; and implementation process in the form of activities, strategies, and common risk factors addressed]?
2. What conceptual frameworks or models are utilized in designing primary school health promotion programs, of which oral health is a part?
3. What are the outcomes of primary school health promotion programs, of which oral health is a part?
4. What are the characteristics [geographical distribution, timeframe, and study population] of the studies that report on primary school health promotion programs, of which oral health is a part?

These questions were framed based on Population Concept Context [PCC] framework [46]. Population included [1] ‘program’ population, i.e., those who were program recipients or beneficiaries or those who were engaged in program delivery and [2] ‘study’ population, i.e., those who were studied to assess the program and may not necessarily have received the program. Program population included primary school children [grades one to six]; parents, caregivers, and families; school personnel [leadership, principals, teachers, support staff, other persons associated with schools]; and health professionals, research staff, policymakers, corporates, and communities.

Concept of ‘integration’ for this review was defined as ‘explicit or implicit inclusion of oral health-related activities within existing school health programs’ through curriculum changes; policy initiatives; skill building activities; preventive services; health screenings; teacher and health worker training; interest holders’ involvement; experiential, participatory, and hands-on approaches’ or other coordinated strategies addressing shared CRFs [e.g., unhealthy diet focused on sugar restriction]. Studies were eligible if they demonstrated this form of integration rather than standalone oral health interventions.

Context includes global primary school settings with programs implemented at public or private school-level. For the purpose of this study, primary or elementary school health programs were defined as those that offered health promotion programs or related activities to children studying in grades one to six.

Following the finalization of research questions and eligibility criteria, the review was conducted in four stages as described below.

### Stage 1: Identification of relevant studies

Based on the definition of integration, peer-reviewed primary studies describing school health promotion initiatives that explicitly included oral health-related activities or implicitly addressed oral health through shared CRFs such as diet, tobacco and/or alcohol use, poor hygiene, stress, or injury were eligible for inclusion For instance, nutrition-focused health promotion programs addressing sugar consumption or personal hygiene activities incorporating oral hygiene in any form were considered to address CRFs and therefore implied inclusion of oral health. During full-text screening and data extraction, we examined the study objectives and methods to identify any oral health-related components and the results sections to identify oral health-related outcomes, even when oral health integration was not explicitly stated. Studies evaluating implementation outcomes – including feasibility, acceptability, barriers, facilitators, sustainability, and cost-effectiveness were also eligible for inclusion.

The search was not limited by publication year or geographical location. Studies describing school health programs without an oral health focus or implied targeting CRFs [as concluded from study objectives, methods, or results] and those not based in primary school settings were excluded. Studies describing interventions for pre-primary, middle, or secondary school grades were included only if the program was clearly described for grades one to six and corresponding findings could be extracted separately. Additional exclusion criteria included letters to editors, commentaries and opinion or perspectives papers, review articles, study protocols, conference abstracts, thesis or practicum reports, program manuals, guidelines, government reports, curriculum and policy documents, studies describing clinical interventions, non-English publications, and unavailability of full text.

Six electronic databases – Scopus, Medline, Embase, Global Health, Eric, and Education Research were searched with assistance from a health sciences librarian experienced in scoping reviews. The initial search was performed in October 2023 and updated in April 2025 and February 2026 to identify new articles. Same search strategy was used during each update. Complete search strategy adopted for each database is presented in S2 Appendix.

### Stage 2: Study selection

Search results from each database were exported to Covidence, a review screening web-based tool [51]. Duplicate entries were removed using automated tools and were also manually cross-checked by two reviewers [NS and NF]. Study selection was performed in two phases using eligibility criteria. After duplicate removal, two reviewers independently screened titles and abstracts [NS and HK] and read full texts of relevant studies [NS and NF]. Reasons for exclusion of full-text studies not meeting inclusion criteria were noted. Reference lists of included articles were checked to enhance identification of pertinent studies.

### Stage 3: Data charting and extraction

Studies selected during full text screening were read independently by two reviewers [NS and NF]. Disagreements were resolved through discussion and consensus. Senior research team member [MA] was consulted as needed. Data extraction form was developed based on research questions using resources from JBI Manual for Evidence Synthesis [47]. The form was pilot tested with five studies and was modified to ensure capturing of relevant information. Unclear or missing information was confirmed by emailing the corresponding author, waiting a minimum of one week to send a final follow-up email. Studies were excluded for lack of sufficient information if responses from authors were not received within a week of the follow-up email. A calibration exercise was conducted to ensure that all reviewers understood and consistently applied the inclusion and exclusion criteria.

### Stage 4: Summarization and presentation of results

Data extracted from included studies are presented in Tables 1–3.

**Table 1:** Study designs used in included sources of evidence.

Study Number	Covidence Study ID	Author(s) and publication Year	Study Design
1	# 2121	Truin et. al. 1981	Cross-sectional
2	# 11130	Knight and Grantham-McGregor 1985	Before-After Study / Pre-Post Intervention Study
3	# 2007	Butcher et al. 1988	Not reported (intervention description implied)
4	# 1668	Freeman et al. 2001	Experimental with block randomization
5	# 17548	O’Neill and O’Donnell 2002	Not reported (study used mixed methods)
6	# 1579	Petersen et al. 2004	Evaluation study (prospective cohort implied)
7	# 1315	Freeman and Oliver 2009	Matched controlled prospective trial
8	# 3416	Franze et al. 2011	Not reported (cross-sectional implied)
9	# 1189	Muirhead and Lawrence 2011	Ecologic (reported)
10	# 989	Monse et al. 2013	Longitudinal cohort design with concurrent and non-concurrent control groups
11	# 1005	Nammontri, Robinson, and Baker 2013	Cluster randomized controlled trial
12	# 927	Yusof and Jaafar 2013	Retrospective cohort
13	# 3024	Reddy and Singh 2015	Not reported (use of explorative mixed methods)
14	# 605	Borges-Yáñez, Castrejón-Pérez, and Camacho 2017	Pragmatic cluster randomized trial
15	# 607	Duijster et al. 2017	Non-randomized clustered controlled trial / Longitudinal cohort
16	# 570	Edasseri et. al. 2017	Prospective cohort
17	# 2865	Reddy and Singh 2017	Not reported (use of mixed methods)
18	# 486	Qadri et. al. 2018	Randomized controlled trial
19	# 18169	Shrestha et al. 2019	Not reported (qualitative study)
20	# 437	Tomazoni et. al. 2019	Cluster randomized controlled trial
21	# 301	Duijster et. al. 2020	Parallel cluster randomized controlled trial
22	# 14348	Riya, Arief, and Kurnia 2020	Quasi-experimental pre-test post-test design with control group design
23	# 2298	Loch et. al. 2021	Not reported (mixed-methods)
24	# 8538	Petrus-Vancea and Stirb 2021	Not reported (case series implied)
25	# 17165	Riglea et. al. 2022	Cross-sectional
26	# 9281	Sharma. et. al. 2022	Cross-sectional (reported)
27	# 18173	Szczepanska et al. 2022	Not reported; Before and after (implied)
28	# 4723	Yamgai et. al. 2022	Not reported (before and after / pre-post implied)
29	# 21	Nishio et. al. 2023	Cross-sectional
30	# 9111	Moussi et al. 2024	Longitudinal study
31	# 21026	Shaikh, Prasidda, and Eruva, 2025	Pre and post intervention design

**Table 2:** Details of program population [Intervention recipients and delivery agents].

Study Number	Covidence Study ID	Author(s) and Publication Year	Program Population (Intervention Recipients)	Program Population (Intervention Delivery Agents)
1	# 2121	Truin et. al. 1981	Children in grades 2, 4, and 6;	School health representative committee (multidisciplinary working group consisting of a physician, a dentist, a dietician and some educators); Teachers; Health service workers
2	# 11130	Knight and Grantham-McGregor 1985	Children in grades 3, 5, and 7 (mean age = 10 years)	Teachers; Principal researcher (assistance)
3	# 2007	Butcher et al. 1988	Children in grades 4–6; Parents; Food service workers in cafeteria	Teachers; Volunteers
4	# 1668	Freeman et al. 2001	Children aged 9 years old; Parents	Teachers; Researchers; Community practitioners; School Board governors; Dieticians; School meal advisors;Health promotion officers; Local dairy suppliers of school milk
5	# 17548	O’Neill and O’Donnell 2002	Children in primary schools, nursery and playgroup; Parents	Health promotion staff; Dietetic staff; Dental staff; Environmental health staff; Education staff
6	# 1579	Petersen et al. 2004	Children in grade 1; Mothers	Teachers
7	# 1315	Freeman and Oliver 2009	Children in grade 5 (9 years); Parents	Teachers; Wholesalers and providers of milk, fruit and vegetables (to support break-time policy)
8	# 3416	Franze et al. 2011	Children in grades 5 and 6 (9–13 years)	Teachers; Dentists
9	# 1189	Muirhead and Lawrence 2011	Elementary and high school children	Community (partnerships); Other delivery agents unclear
10	# 989	Monse et al. 2013	Children in grade 1 for intervention group (mean age = 7.56 years); Children in grades 2 and 4 for control groups (mean age of control group 1 = 7.39 years; mean age of control group 2 = 7.47 years)	Teachers; Nurses
11	# 1005	Nammontri, Robinson, and Baker 2013	Children in grade 5 (10–12 years)	Teachers; Researcher support to deliver activities
12	# 927	Yusof and Jaafar 2013	Children in grades 4–6 (10–12 years at endline)	Teachers; Peers (Dokter Muda conducted health talks)
13	# 3024	Reddy and Singh 2015	Primary school children	Schols through curriculum (presumably teachers); Department of Health; Community (partnerships)
14	# 605	Borges-Yáñez, Castrejón-Pérez, and Camacho 2017	Children between 6–13 years; Parents/guardians (mean age = 36.9 ± 8.5 years) and 90% were mothers or grandmothers	Teachers; Program supervisors
15	# 607	Duijster et al. 2017	Children in grade 1 (6–7 years)	Educational staff
16	# 570	Edasseri et. al. 2017	Children aged 8–10 years (at baseline) and 10–12 years (at endline); Parents and families; Teachers; School management; Community	Nutritionist; Dental health professionals; Community (partnerships)
17	# 2865	Reddy and Singh 2017	Children in grade 1; Parents; Educators	School health teams; School nurses; Colgate World of Care; Department of Health; Community (partnerships)
18	# 486	Qadri et. al. 2018	Children in grade 5 at baseline; Children in grade 6 at endline (9–12 years); Parents	Teachers; National Health System
19	# 18169	Shrestha et al. 2019	Children in grade 5 (as per response from author)	Teachers; Community (mobilization)
20	# 437	Tomazoni et. al. 2019	Children in grades 4 and 5	Teachers; Researcher
21	# 301	Duijster et. al. 2020	Primary school children	Teachers; Peers (student leader)
22	# 14348	Riya, Arief, and Kurnia 2020	Children in grades 4 and 5	Facilitators; Researcher
23	# 2298	Loch et. al. 2021	Children aged 5–12 years	Teachers; School principal; Support staff; Trained and calibrated students and staff from dentistry, oral health and human nutrition; Peers (older students supporting younger students)
24	# 8538	Petrus-Vancea and Stirb 2021	Children in grades 5, 6, and 7; Parents	Teachers
25	# 17165	Riglea et. al. 2022	Elementary school children	Teachers; Nurses; Social workers; Dental hygienists; Community partnerships
26	# 9281	Sharma. et. al. 2022	Children in grades K to 2; Parents	Teachers; School administration; Trained project staff
27	# 18173	Szczepanska et al. 2022	Children in grade 2 (7–9 years)	Teachers
28	# 4723	Yamgai et. al. 2022	Primary school children; Parents / caregivers; Teachers; Canteen operators; School administration; Government	Teachers; Staff from non-government organization (NGO); Community (partnerships)
29	# 21	Nishio et. al. 2023	Primary, middle, and high school children	Schools through curriculum (presumably by teachers)
30	# 9111	Moussi et al. 2024	Children in grades K to 7 (3–12 year)	Nurses
31	# 21026	Shaikh, Prasidda, and Eruva 2025	Children in grades 1–5 (6–12 years)	Paediatricians and Preventive and Social Medicine (PSM) experts

**Table 3:** Key results from included studies.

Study Number	Covidence Study ID	Author(s) and Publication Year	Program Duration Reported	Oral Health Component(s) Reported	Common Risk Factor(s) Identified	Program Implementation Strategies Identified	Key Outcomes Reported
1	# 2121	Truin et. al. 1981	8 to 9 years	Dental health	Unclear	Building health-related skills through classroom instruction, school engagement, teacher involvement, didactic materials for teachers, other strategies	• Significant reduction in decayed and filled surfaces (highest among 7-year-olds) • Increase in caries-free children (highest among 7-year-olds) • Initial increase in fluoride tablet use in 9- and 11-year-olds, followed by decrease
2	# 11130	Knight and Grantham-McGregor 1985	10 months (one school year)	Healthy diet (good and bad foods for health), care of teeth	Unclear	Building health-related skills through classroom instruction and group activities, teacher involvement, parental engagement, professional supervision of activities	• Improvements in knowledge of immunization and dental care among children • Increase in guardians’ knowledge about tooth decay
3	# 2007	Butcher et al. 1988	Unclear	Nutrition, diet	Unhealthy diet	Building health-related skills through group activities, teachers training, didactic materials for teachers	Not applicable
4	# 1668	Freeman et al. 2001	2 years	Healthy diet	Unhealthy diet	School policy, teacher involvement, principal involvement, parental engagement, community partnerships	• Significant increase in DMFT (Decayed Missing Filled Teeth) and mean number of missing teeth and filled teeth • Moderate increase in mean number of sound permanent teeth among children in intervention schools
5	# 17548	O’Neill and O’Donnell 2002	3 years	Healthy foods	Unhealthy diet	School policy, changes in physical and/or social environment, parental engagement	Perceptions of children: • All children were aware of scheme and importance of healthy snacking, ate lunch better, were less interested in sugary and fatty foods, and some wanted to consume ‘naughty’ snacks once a week Perceptions of schools, teachers, and parents: • Schools perceived significant improvements in litter reduction; school meal wastage; children’s behaviours, alertness, and concentration; and dental health after scheme introduction • More than 70% schools discussed usefulness of providing resource pack and fruits and vegetables • Control schools considered scheme criteria restrictive, were involved in another award, and did not participate • Teachers felt that children (58%) were more aware of healthy eating as a result of the scheme • As per teachers, children brought fruits and vegetables for break time, encouraging healthier choices, leading to incorporation of scheme in curriculum • Teachers requested – (i) suggestions on dealing with class parties and rewards, (ii) assistance with providing fruits and vegetables in school, (iii) a local contact person for healthy breaks scheme, and (iv) resources and teaching materials • Teachers and parents stated that if children skipped breakfast, they would consume additional snacks during snack time • While parents were happy with the scheme and felt that children ate their lunch better and were less interested in sugary and fatty foods, a small proportion considered scheme criteria to be strict and suggested inclusion of sandwiches
6	# 1579	Petersen et al. 2004	3 years	Oral health, teeth and their functions, dental plaque, tooth decay, diet, sugar, and health (general and dental), self-care for oral health, importance of dental visits	Unhealthy diet	Building health-related skills through supervised actions and group activities, teachers training, didactic materials for teachers, teacher involvement, principal involvement, parental engagement, professional supervision of activities, making oral health part of existing curriculum	Outcomes related to children: • Mean increments in filled surfaces (primary teeth) was 0.33 and 0.06 and of filled surfaces (permanent teeth) was 0.16 and 0.03 in experimental and control groups respectively • Children’s twice-a-day toothbrushing, fluoride toothpaste use, and dental visits were significantly higher in experimental group (26%, 11%, and 10% respectively) compared to control group (19%, 5%, and 3% respectively) • Cake and biscuit consumption frequency declined in by 5% experimental group compared to 5% increase in control group Outcomes related to teachers and parents: • Knowledge and positive attitude of teachers in experimental group changed (40% and 20% respectively) compared to control group (5% and 8%) • Higher proportion of teachers allocated more time for oral health education and delivering instructions to children in experimental group (34%) versus control group (7%) • Significantly higher proportion of mothers in experimental group performed weekly cleaning and checking their child’s teeth after brushing
7	# 1315	Freeman and Oliver 2009	2 years	Healthy diet (healthier snacking habits in school and wider environment)	Unhealthy diet	School policy, teacher involvement	• At baseline, 60% children had at least one sugar snack in their rubbish bag and 47% visited shops around school hours, which increased to 100% and 63% respectively at follow-up • Frequent snack-related rubbish was found among children stopping on the way home from school • Children in policy schools initially consumed healthier snacks versus control school children who consumed more chocolates and crisps. By 24 months, both groups showed increased consumption of chocolates, crisps, and sugary drinks • Within schools, intervention group children had significantly lower mean baseline scores for sugar snack scores, but equivalent mean follow-up sugar snack scores compared to children attending control schools • Intervention had no effect on sugar snack scores outside the school Decay at follow-up was predicted by school intervention status and evidence of sugar snacks consumption outside school and at home
8	# 3416	Franze et al. 2011	15 months	Cariogenic impact of sugar- and carbohydrate-containing foods; role of dental plaque; fluoride toothpaste; brushing technique and frequency; cariogenic snacks	Unhealthy diet, tobacco use	Building health-related skills through group activities, student empowerment, teachers training, didactic materials for teachers	• Low refusal rates indicated acceptability of program and teacher training • Participating teachers from intervention schools shared that program occurred on health days and was integrated within school mission statement; however, it was not implemented within classes (during first implementation meeting), reporting inclusion in classes during second meeting
9	# 1189	Muirhead and Lawrence 2011	1 year	Healthy eating, injury prevention, substance use and abuse	Unhealthy diet, tobacco use, injury	Building health-related skills through classroom instruction, changes in physical and/or social environment, improving access to resources, community partnerships	• Participating schools, especially those that engaged in multiple (more than three) health-related activities had significantly lower percentage of children with more than two decayed teeth and requiring urgent dental treatment compared to schools with one to two health-related activities or no health promotion • Among low-income schools, those participating had lower proportion of children with more than two decayed teeth, requiring preventive care and treatment, compared to those in non-participating schools
10	# 989	Monse et al. 2013	1 year	Oral hygiene	Poor hygiene	Building health-related skills through group activities, changes in physical and/or social environment	• No changes in prevalence of caries and dental infections • Significantly higher increase in mean body mass index (BMI) • Lower prevalence of moderate to heavy soil-transmitted helminths infection than control group (after year 1)
11	# 1005	Nammontri, Robinson, andBaker 2013	1 month	Toothbrushing; healthy mouth; healthy food choices; positive thinking about health, self, others, and mouths; inevitability of oral diseases; health as a continuum; body image, self-care, self-efficacy, self-esteem, and self-confidence; satisfaction rating related to oral health and how to improve it; acceptance of heart or oral diseases	Unhealthy diet, stress	Building health-related skills through supervised actions, classroom instruction, and group activities; student empowerment; teachers training; didactic materials for teachers; teacher involvement; whole-school approach; school engagement; making products available; school policy	• Intervention group showed higher sense of coherence and reported better oral health-related quality of life (lower score on Child Perception Questionnaire) than the control group at 2 weeks and 3 months post-intervention • At 3 months, a higher proportion of children in intervention group had healthy gingivae than those in control group
12	# 927	Yusof and Jaafar 2013	6 years	Oral hygiene, anti-smoking	Poor hygiene, tobacco use	Building health-related skills through group activities, student empowerment, teachers training, teacher involvement	• Intervention group reported fewer impacts on tooth cleaning and less severe impacts on speaking and emotional well-being • Significantly lower proportion of intervention children reported bleeding gums and plaque/calculus compared to control group • Overall oral impacts on daily performance were similar between intervention and control groups
13	# 3024	Reddy and Singh 2015	Unclear	Oral health education, toothbrushing, other preventive treatments (rarely)	Unclear	Building health-related skills through classroom instruction, community partnerships	• Policies included statements on oral health promotion, but was not translated into practice at school level • Program implementation challenges included lack of funds, human resources, knowledge and ownership, as well as high workloads and time constraints
14	# 605	Borges-Yáñez, Castrejón-Pérez, and Camacho 2017	Unclear	Toothbrushing (oral hygiene), healthy eating (nutrition)	Unhealthy diet, poor hygiene	Building health-related skills through supervised actions and classroom instruction	• Significantly higher mean percentage differences for plaque-free surfaces (8.8 ± 28.5%) and healthy gingival units (23.3% ± 23.2%) in intervention group
15	# 607	Duijster et al. 2017	2 years	Group toothbrushing (dental hygiene)	Poor hygiene	Building health-related skills through supervised actions and group activities, teachers training, teacher involvement, changes in physical and/or social environment, making products available, whole-school approach	• Lower increment (average of 24%) in number of decayed, missing, and filled permanent teeth between baseline and follow-up among children in intervention schools in all three countries
16	# 570	Edasseri et. al. 2017	Unclear	Dental care	Unclear	Teacher involvement, principal involvement, school engagement, parental engagement, changes in physical and/or social environment, community partnerships, whole-school approach	• Three distinct school environment types were identified: ◦ Type 1 schools having strong healthy eating programs and favourable neighbourhood food environments ◦ Type 2 schools having strong healthy eating programs and unfavourable neighbourhood food environments ◦ Type 3 schools having weak healthy eating programs and unfavourable neighbourhood food environments • Adjusting for potential confounders, children attending Type 1 and 2 schools had 21% and 6% lower 2-year incidence of dental caries, respectively, compared with Type 3 schools
17	# 2865	Reddy and Singh 2017	Unclear	Toothbrushing, fluoride mouthrinse, fissure sealant, oral health awareness, unhealthy diet, nutrition	Unhealthy diet, poor hygiene	Building health-related skills through supervised actions, teacher involvement, school policy, making products available, parental engagement, community partnerships, making oral health part of existing curriculum, other strategies	Overall Outcomes: • Health messages formed part of curriculum (96% schools) • Program created oral health awareness for educators / parents (23% schools) and awareness of correct brushing technique (31% schools) • Daily toothbrushing was conducted by only 8 schools • Only 27% students were caries free and 90% required preventive care Reported Challenges: • Staff were not confident in implementing health curriculum due to lack of oral health knowledge • Time constraints limited implementation (70% schools) • Low staff numbers resulted in higher workloads (8% schools) • Students chewed toothbrush / ate toothpaste (23% schools) • Lack of adequate basins, taps, and cups for rinsing and storage Learners left the space untidy, leading to time wastage in cleaning • Hygienic storage of toothbrushes and toothpaste supplies was challenging, especially in larger classes • Labelling and distribution of toothbrushes was time consuming • Learners did not have toothbrushes and paste to brush their teeth at home (92% schools) • Educators recommended parents to purchase supplies, but this was unaffordable for parents • Educators sent toothbrushes and toothpaste home for learners to use; however, some learners did not use them at home or lost them • Availability of some support for oral health interventions (39% schools) • No budget for oral health from provincial Department of Basic Education • Support from Colgate World of Care and provincial Department of Health was not continuous and was dependent on availability of supplies, and only one school had nurse support • Water access was a major barrier to intervention implementation and success (38% schools) • Resource availability was a challenge to program success (100% schools) • Program discontinued once supplies were depleted due to inability to secure sponsorships for supplies and large classes
18	# 486	Qadri et. al. 2018	Unclear	Tooth paste use, brushing. and fluoride use (oral hygiene); dental caries prevention and management; healthy eating (nutrition); smoking	Unhealthy diet, tobacco use	Teacher training, making oral health part of existing curriculum, other strategies	• 35% higher caries risk was observed in control group, versus 94% reduction in incidence risk ratio associated with high socio-economic status (p < 0.001)
19	# 18169	Shrestha et al. 2019	4 years	Oral health, oral health check-ups	Unclear	Building health-related skills through classroom instruction and group activities, teachers training, teacher involvement, school policy, changes in physical and/or social environment, other strategies	• Improved knowledge of health, nutrition, hygiene, and sanitary practices among students • Positive changes in students’ nutritional behaviours, hygiene practices, and life skills • Improved school attendance, enrolment, retention, and academic performance among students • Parents started sending tiffin from home and started teeth brushing • Reduction in open defecation, diarrhoea, parasitic infections, anaemia, blindness, and hearing loss among students; and improved cooperation between schools and community, communities were mobilized leading to community awareness and building of more toilets • Challenges in program implementation included lack of stakeholder coordination and planning for sustainability and scale-up; insufficient funds, human resources and physical infrastructure; and limited training opportunities • Stakeholders suggested integrating program in government system, active involvement of Ministry of Education, coordination between stakeholders to generate resource, and frequent training of teachers, expanded to other parts of the country
20	# 437	Tomazoni et. al. 2019	2 months	Oral health and healthy mouth; thinking positively about health, self, others, and condition of mouth; inevitability of oral diseases and health as a continuum; good body image, self-efficacy, and self-confidence; how to improve and maintain health; brushing teeth effectively under supervision of teachers and researchers; healthy food choices	Unhealthy diet, stress	Building health-related skills through supervised actions, classroom instruction, and group activities; student empowerment; teachers training; didactic materials for teachers; teacher involvement; whole-school approach; school engagement; making products available; school policy	• Children in intervention group had significantly improved sense of coherence predicting better quality of life and fewer impacts of oral health on daily lives compared to those in control group at 3-month post intervention • Improved sense of coherence, older children, high education of mothers, lower DMFT scores, and intervention indirectly predicted better functional status at 3-month follow-up via better perceived symptoms • Higher age of child and intervention also indirectly predicted better functional status at 3-month follow-up via sense of coherence improvement • Intervention predicted better-perceived symptom at 3-month follow-up via sense of coherence improvement
21	# 301	Duijster et. al. 2020	Unclear	Toothbrushing (oral hygiene)	Poor hygiene	Building health-related skills through supervised actions; changes in physical and/or social environment	• Outcomes included no significant difference in handwashing and toothbrushing behaviours and plaque score between intervention and control schools, four-months post-intervention
22	# 14348	Riya, Arief, and Kurnia 2020	Less than 1 month	Toothbrushing (oral hygiene)	Poor hygiene	Building health-related skills through supervised actions and classroom instruction	• Personal hygiene knowledge increased from 8% to 97% in treatment groups, with no change in control group • Positive attitudes towards personal hygiene increased from 61% to 100% at post-test in treatment group, with slight decrease in control groups (100% to 92%) • Hand hygiene and tooth brushing practices increased from 0% to 92% and from 3% to 100% respectively, with no changes in control group
23	# 2298	Loch et. al. 2021	6 months	Sugar and sugary drinks and their effects; identify hidden sugars in sugary drinks and foods; microorganisms in body and mouth; effect of acid on teeth; how can we protect our teeth; how fissure sealants and fluoride can protect our teeth; what do oral health professionals do; dental anxiety; brushing frequency and technique	Unhealthy diet	Building health-related skills through group activities, teacher involvement, principal involvement, professional supervision of activities, other strategies	• Improvements in oral hygiene habits, diet, and related knowledge, including understanding of effect of fluoride on teeth • Decline in daily consumption of sugary drinks • Not all dental terminology and scientific knowledge was retained long term, but improvements were observed when messages were reinforced • Awareness on oral health practices may not translate into implementation
24	# 8538	Petrus-Vancea and Stirb 2021	1 year	Maintaining oral health, influence of nutrition on oral health, human anatomy and physiology including oral cavity hygiene	Unhealthy diet, poor hygiene	Building health-related skills through classroom instruction, curriculum redesign/restructuring, parental engagement, making oral health part of existing curriculum, school engagement, other strategies	• Occurrence of cavities acknowledged by 66% grade 5 students versus 25–33% from all grades unable to do so at baseline • Responses of students from grades 6 and 7 were concentrated on bad breath during baseline • Increased awareness of dental cavities • At post-test, all students were aware that dental problems lead to other diseases.
25	# 17165	Riglea et. al. 2022	1 year	Dental health and oral hygiene, tobacco use prevention	Tobacco use	Building health-related skills through classroom instruction and group activities; teacher involvement; community partnerships; other strategies	• Relatively few principals viewed dental problems (30%) or cigarette smoking (5%) as important • More than 80% schools offered health promotion interventions (HPIs) related to oral health, bullying, physical activity, and sex education • Less than half the schools offered HPIs related to injury prevention (45%) or mental health (42%), and only 9% offered tobacco control HPIs • HPI availability generally aligned with perceived importance of a health issue (91% of principals reported no tobacco control in their school and 87% of them regarded smoking as unimportant; 94% schools reported interventions, but only 29% principals considered dental problems important.
26	# 9281	Sharma et. al. 2022	1 year	Regular dental care and visits, oral hygiene, toothbrushing, and flossing; dental trauma and injury prevention; unhealthy diet and nutrition (tooth-healthy and unhealthy foods and beverages - sugary, starchy, sticky); building self-efficacy (for healthy eating, drinking, mealtime, and snacking behaviours) and focus on subjective norms	Unhealthy diet, injury	Building health-related skills through supervised actions and classroom instruction, teachers training, didactic materials for teachers, teacher involvement, professional supervision of activities, parental engagement, school engagement	• All lessons and activities implemented • Adequate equipment was available • Variability in implementation existed • Majority of teachers and physical education coaches were enthusiastic about activities, including school toothbrushing • Lessons and activities, especially toothbrushing activity was enjoyed by majority of the children • 77% of toothbrushing was conducted in bathroom after lunch and 23% was conducted in classroom as part of a class
27	# 18173	Szczepanska et al. 2022	24 months (two 12-month cycles)	Healthy nutrition	Unhealthy diet	Building health-related skills through classroom instruction and group activities, student empowerment	• Significant post-intervention knowledge gain (99.05%) for both girls and boys (p < 0.0001)
28	# 4723	Yamgai et. al. 2022	Unclear	Unhealthy diet and nutrition (foods high in fibre, fat, sugar, salt, minerals); usual breakfast and favourite foods; toothbrushing (oral hygiene)	Unhealthy diet, poor hygiene	Building health-related skills through group activities, teacher involvement, school engagement, involvement of school canteen, parental engagement, involvement of PTA (Parent Teacher Association), changes in physical and/or social environment, improving access to resources, making products available, community partnerships, political involvement	• Significant increase in overall nutrition attitude score • Food choices and handwashing improved significantly, with boys being more competent • Nutrient source competence was negatively affected, especially among girls • Children over 9 years showed significant increases in knowledge, attitude, practices, food choices, and handwashing • Children under 10 years significantly improved in practices and handwashing while decreasing their score in dental care • One school engaged in a nutrition education promotional activity during the Global Handwashing Day, while another school exploited recipes learned during nutrition course to ensure children’s regular protein intake
29	# 21	Nishio et. al. 2023	Unclear	Oral health education (no sugar, no alcohol, no smoking); injury prevention	Unhealthy diet, injury	Making oral health part of existing curriculum	• Hygiene and oral health were more likely to be taught in primary schools than in high schools in all countries as part of health, physical education, sciences, social studies, home economics, morals, religion, and language (except FSM and Palau) • Mental health and healthy eating were taught in all countries (except FSM) as part of moral or religion in Japan on Indonesia Nutrition and oral health were taught as part of health, morals, home economics, and social studies
30	# 9111	Moussi et al. 2024	Unclear (took place between November 14–19, 2022)	Oral hygiene	Poor hygiene	Building health-related skills through classroom instruction	• Significant increase in overall knowledge and practice scores of healthy lifestyles among 8- (34.09%) and 11-year-old (30.21%) children • Scores influenced by governorate, normal-weight status, and close supervision
31	# 21026	Shaikh, Prasidda, and Eruva, 2025	4 weeks (one month)	Dental care	Poor hygiene	Building health-related skills through classroom instruction, professional supervision of activities	• Significant increase in personal and oral hygiene behaviours • Decrease in diarrheal episodes, and respiratory and skin infections • Hygiene improvement significantly associated with reduced morbidity

Both quantitative and qualitative inductive content analysis of the data were performed as per research questions. Included studies are summarized through descriptive statistics. Results also include a narrative description of general health programs and oral health interventions. Common risk factors targeted through the programs described in each study, implementation strategies, and outcomes have also been listed. Risk of bias assessment was not performed. Inductively developed codes and categories [47] from extracted data can be found in S3 Appendix.

Protocol for this scoping review was registered in Open Science Framework on May 25, 2025 [Registration DOI: https://doi.org/10.17605/OSF.IO/Y3E2V].

## Results

### Study selection

Our search yielded a total of 27158 records. After removing duplicates, 4390 records were screened, of which 324 articles were considered for full-text review. Final synthesis included 31 studies. Fig 2 shows PRISMA flow diagram illustrating the search and selection process. Reasons for exclusion of studies during full-text review are presented in S4Appendix.

**Fig 2:**
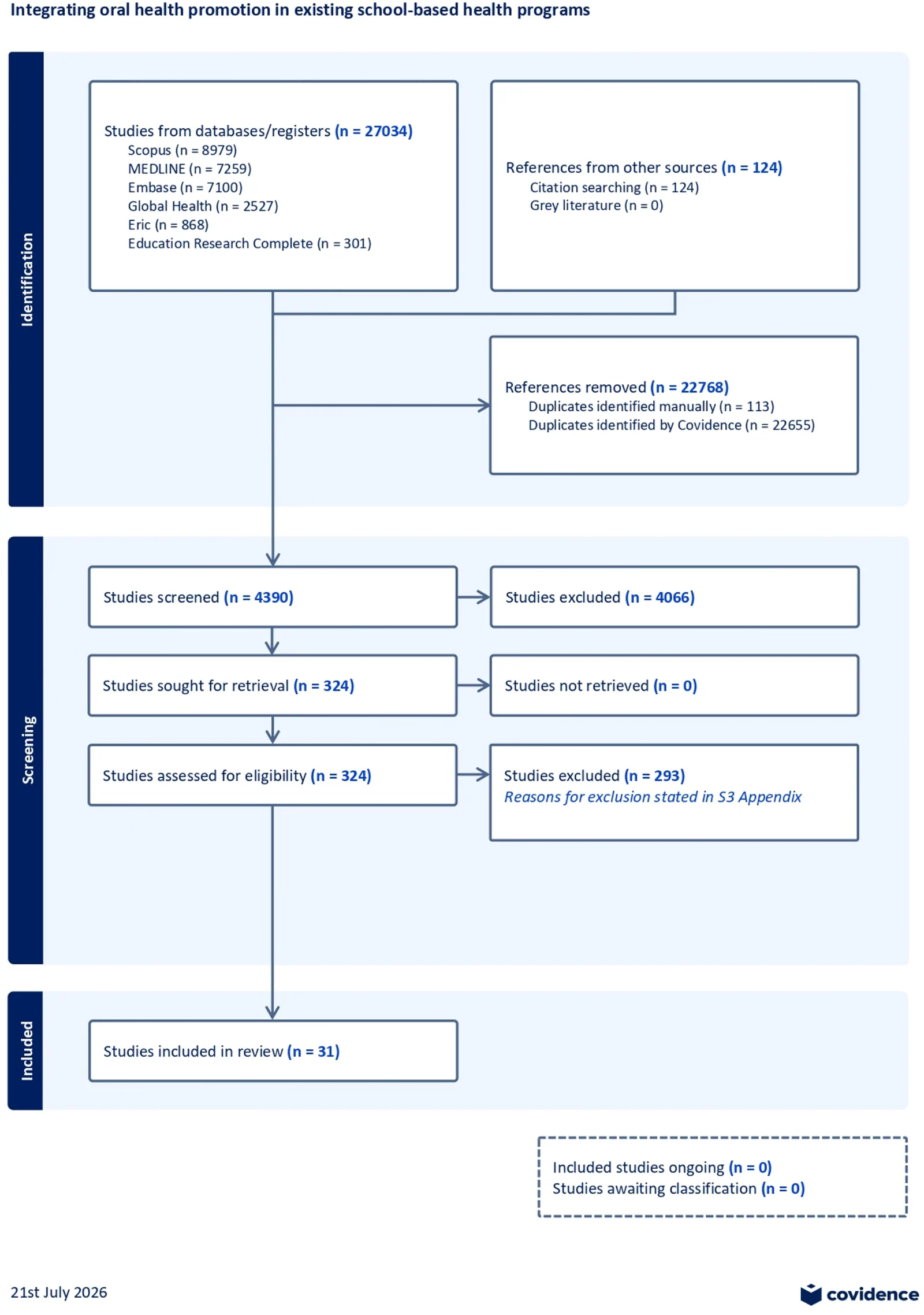
PRISMA flow chart.

### Characteristics of included studies

Included studies were published between 1981 and 2025. Fourteen were conducted in high-income countries in North America [52–56] Europe [57–64] and the Pacific [61]. Nine were carried out in upper middle-income regions in South-east and East Asia [65–68], Africa [69–72], South America [60], North America [59], and Caribbean [73]. Only a handful [n = 6] were based in lower middle-income regions of South-east Asia [74,75], South Asia [70,76], Africa [71], and Middle-east [72]. None represented low-income countries. Two were multi-country studies spanning more than one income region [62,77]. Fig 3 shows geographical distribution of studies based on World Bank Group Income Classifications [July 1, 2025 – June, 2026] [78].

**Fig 3:**
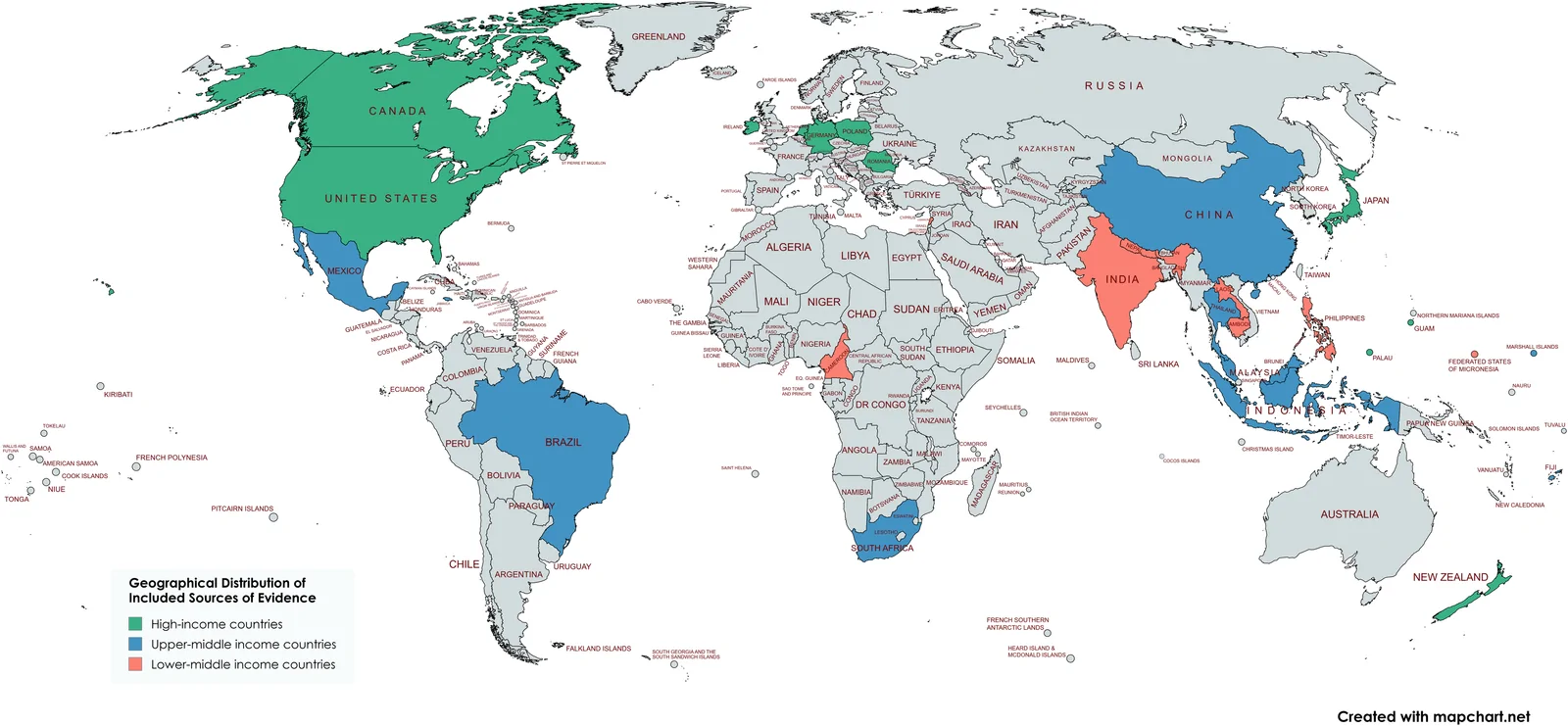
Geographical distribution of included sources of evidence.

Twenty four studies primarily focused on investigating or comparing outcomes, including program feasibility, efficacy, effectiveness, impact, and evaluation [52,53,56–62,64–68,70–76,79–81]. Remaining examined program efficiency and sustainability [82]; program contents (topics covered) and components or activities implemented [77,80]; methodology and methods [75,80], intervention framework [52], and program implementation as well as challenges [71] – either independently or in addition to outcomes assessment. Three studies reported aims that could not be categorized into any group [54,55,83]. Only one did not explicitly state the aims [63].

Study design was reported in 21 studies [Table 1], and included randomized controlled trials [57,59,60,65,74,81]; cross‐sectional [52,55,64,77]; longitudinal cohort [53,67,72,75]; non-randomized [62,84]; pre-post interventional [68,70,73]; evaluation [66]; and ecological studies [56]. Remaining 10 studies did not mention study design, although one was qualitative [76] and four employed mixed-methods [61,63,82,83].

### Program population

Included studies reported on two related groups – study population (participants from whom data were collected) and program population [intervention recipients or delivery agents]. Information on study aims, study population, and sample size can be found in S5 Appendix. Details of program population are summarized in Table 2.

Primary school children constituted the program population receiving intervention in all studies. Close to half the studies [n = 15] reported children’s age [53,57–59,61,62,65,67,70,72,73,75,80,81,84]. ranging from 3 to 13 years. In addition to primary school children, few studies also reported children studying in pre-schools, kindergarten, middle schools, and high schools [52,56,63,72,73,77,79].

Twelve studies reported parents, guardians, or caregivers as the program population [52–54,57,59,63,66,71,79,82,84,85] receiving intervention through receipt of educational materials, social support, school policy or program updates, and meal preparation guidance; supervising children’s hygiene behaviours and meal planning; and participation in meetings, committees, or policy consultations. Mean age of parents was reported in only one study as 36.9 ± 8.5 years [59].

Seven studies reported engagement of school leadership, administration, or principals [52,53,57,61,71]; school canteen operators or food service workers [54,71]; school health teams [82], and other school staff [61] in advocacy, awareness, behaviour change, program planning, or related activities.

Teachers were the program population primarily engaged as intervention delivery agents in majority of the studies [n = 25] [52–55,57–62,64–67,69,71,73–75,77,79,80,84–86]. Their involvement included delivering classroom education; handwashing and toothbrushing supervision; supporting policy implementation; participation in consultations, trainings, or other program-related tasks, and less frequently in receiving awareness sessions and behaviour change interventions. Three studies included student peers in program delivery [61,67,74]. Three studies reported engagement of educators or educational staff, without specifying who constituted these personnel [62–64,82].

Four studies reported nurse involvement [55,72,75,82] in delivering educational activities and preventive services.

Studies also reported engaging dieticians, nutritionists, or school meal advisors [53,57,63,64]; dental professionals [53,55,63,64,80]; physicians and medical experts [64,70]; dentistry and nutrition students [61]; allied health professionals [57,63,64]; local wholesalers, suppliers, provider of milk, fruits, and vegetables [57,84]; non-government or community organizations, community practitioners, volunteers, social workers, and community [53–57,71,82,83,86]; project staff or researchers [52,57,59,60,65,68,73];as well as government [69,71,82,85] and private sector [82] in intervention planning, design, delivery, monitoring, supervision, partnerships, mobilization, and providing program, policy, or partnership support.

Program population was unclear in one study [56].

### Program duration and content

Of the 21 studies reporting program duration [Table 3], six were short-term – lasting less than a month to 10 months [60,61,65,68,70,73], five lasted one-year [52,55,56,75,79], and ten were long-term programs exceeding one year [57,58,62–64,66,67,76,80,84].

Most commonly addressed general health topics were diet and nutrition identified in 24 studies [52–67,71,72,77,79–82,84], followed by personal hygiene in 15 [58,59,62,67,68,70–72,74–77,79,82,83]. Vaccination, pain, sleep, screen time, adolescent health, HIV/AIDS, growth and development, infectious disease, human body, deworming, iron supplementation, and first aid were covered in 14 studies [55,56,58,62,67,72,73,76,77,79–83].

Within oral health interventions, programs most commonly focused on oral hygiene and dental care, reported in 24 studies [52,53,55,59–62,64–68,70–76,79–83]. Diet and nutrition was reported in 19 studies [52,54,56–61,63,65,66,71,73,77,79–82,84]. Less frequently addressed topics included oral diseases and related concepts in six [60,61,65,79–81]; substance use, including tobacco and alcohol use in five [55,56,67,77,81]; skill-building components such as positive thinking, body image, and subjective norms in three [52,60,65]; dental trauma or injury prevention in three [52,56,77] studies; and dental anxiety in one study [61].

CRFs were identified in 26 studies, of which unhealthy diet [n = 18] [52,54,56–61,63,65,66,71,77,79–82,84] was the most frequently identified CRF, followed by poor hygiene [n = 11] [59,62,67,68,74,75,77–79,82]; tobacco use [n = 5] [55,56,67,80,85]; injury [n = 3] [52,56,77], and stress [n = 2] [60,65]. Twelve studies addressed more than one CRF [52,56,59,60,65,67,71,77,79–82].

Oral health contents and CRFs addressed are shown in Table 3. Contents of general health programs can be found in S6 Appendix.

### Program activities [General and oral health promotion]

All, except two studies did not report general [56,61] and oral health activities [56,58].

General health programs most commonly included workshops, group projects, hygiene routines, and physical activity; interest holder engagement, empowerment, and advocacy; and classroom teaching, discussions, and instructions [n = 27] [52–55,57–60,62–68,70–75,79–84]. Most frequently implemented oral health-related activities comprised of health education, instructions, and discussions; interest holder engagement, empowerment, and advocacy, structured toothbrushing, and experiential learning through workshops or group activities [n = 26] [52–55,57,59–68,70,72–75,79–84]. Less commonly reported activities included monitoring and professional support, dental screenings and service provision; teacher training; use of educational materials; school nutrition policies and environmental changes; curriculum inclusion or restructuring; provision of supplies; events and campaigns; and peer support [n = 25] [52–55,57,59–67,71,73–75,77,79–84]. Overall, interactive and participatory approaches such as workshops, group activities, and interest holder engagement were widely implemented, while structural or policy interventions were less frequent.

Details of activities implemented as part of general and oral health programs reported across included studies are available in S6 Appendix.

### Program implementation strategies

Table 3 shows program implementation strategies identified in included studies, grouped in following categories: [1] Student skill-building or empowerment [n = 25] [52,54–56,58–62,64–68,70–76,79,80,82,83]; [2] Teacher-focused [n = 19] [52–55,57,60–62,64–67,71,73,76,80–82,84]; [3] Interest holder involvement [n = 16] [52,53,55–57,60,61,63–66,71,73,79,82,83]; [4] Changes in school structure or policies [n = 13] [53,56,57,60,62,63,65,71,74–76,82,84]; [5] Other strategies, including whole school approach [n = 11] [53,55,60–62,64,65,76,79,81,82]; [6] Oral health inclusion in existing curriculum or curriculum restructuring [n = 5] [66,77,79,81,82]; [7] Professional support [n = 5] [52,61,66,70,73]; and [8] Improving resource access or product availability [n = 6] [56,60,62,65,71,82]. All studies utilization of multiple strategies, except two that used single strategy [72,77].

### Program Design [Frameworks, models, and theories]

None of the included studies reported integration-focused models or implementation science frameworks to guide program design. Only four specified a theoretical or conceptual framework. One applied social cognitive theory alongside Centres for Disease Control and Prevention Coordinated School Health Component Model to address diet and physical activity [52]. Another drew on social learning theory, emphasizing self-efficacy through knowledge acquisition, skill mastery, and positive role-modelling [54]. A third was based on Nutbeam’s health literacy framework [80]. One study employed *Fit for School action* framework [75].

Interventions in two studies were informed by prior evidence, consultations, expert input, and identification of successful intervention enablers [60,65].

### Reported outcomes

Table 3 shows oral health-related outcomes reported in 31 studies. All outcomes were grouped into four broad categories.

Child-level outcomes [n = 26] included: [1] clinical outcomes, such as increase in caries-free and plaque-free status as well as healthy gums; reduction in decayed, missing, and filled teeth; and lower dental treatment urgency [n = 13] [53,56,57,59,62,64–67,74,81,82,84] as well as improvements in general health and academic outcomes [n = 3] [70,75,76]; [2] behavioural outcomes, including improvements in oral hygiene practices, toothbrushing behaviours, toothpaste and fluoride tablet use, dental visits, dietary habits and food choices, and overall healthy lifestyle behaviours [n = 10] [61,64,66,68,70–72,74,76,84]; [3] knowledge and attitude outcomes, reflecting changes in understanding of personal and oral hygiene, dental caries, importance of fluoride, diet and nutrition, healthy snacking, and healthy lifestyles [n = 9] [58,61,63,68,71–73,76,79]; and [4] oral health-related quality of life [n = 3] [60,65,67].

Outcomes related to educators and parents included changes in knowledge and attitudes in five studies [52,55,66,73,82] and behavioural outcomes such as increased engagement in children's oral hygiene and nutrition practices as well as education in three studies [66,71,76].

One study reported perceptions of school, teachers, and parents on children's oral health knowledge, behaviours, and clinical outcomes [63].

Ten studies reported program-related outcomes. These included description of health curriculum [77] and program implementation [52,55,71,82]; feedback and suggestions from children, parents, and/or teachers [52,63,76,80]; implementation challenges [76,82,83]; and changes in oral health-related school policies, including strong healthy eating programs, formal policy statements, and supportive neighbourhood environments [53,76,83].

## Discussion

This study provides a comprehensive overview of how oral health promotion has been integrated within broader primary school health programs across diverse contexts over the past four decades. Findings reveal both encouraging trends and notable gaps to inform future program design, policy, and research, based on which key recommendations have been summarized in Table 4.

**Table 4:** Summary of recommendations for integrated school health programming.

Domain	Key Recommendation
Program Design	Integrate oral health with broader school health initiatives using the Common Risk Factor (CRF) Approach
	Embed oral health within existing school health policies and curricula
Implementation	Use appropriate behavioural and implementation science theories and relevant frameworks of integration to guide program design and evaluation
	Engage multiple interest holders (education, health, families, communities, policymakers)
	Adapt programs to local context and prioritize equity
Evaluation	Assess implementation outcomes (adoption, acceptability, feasibility, fidelity, sustainability) alongside effectiveness
	Include system-level and equity-related outcomes
Future Research	Conduct long-term studies using rigorous study designs
	Expand research in low- and middle-income countries

### Geographical and methodological scope

Despite the wide distribution of included studies across 29 countries, the literature remains clustered in high- and upper-middle income counties in North America, Europe, and certain Southeast Asian countries, limiting generalizability. This geographic imbalance is particularly concerning given the disproportionate burden of oral diseases in low- and lower middle-income countries [1,2] where school health programs are often under-resourced [87]. The earliest study was published in 1981; however, overall number of studies remains limited. Theis highlights slow progress in systematically embedding oral health within school health frameworks.

Most studies relied on quantitative study designs with fewer examples of mixed-methods or qualitative approaches. While quantitative evidence provides insight into program effectiveness, lack of qualitative exploration limits understanding of implementation processes, contextual adaptations, and interest holder perspectives [88,89]. Furthermore, considerable proportion of studies did not report research methods clearly, highlighting challenges in reproducibility. Our findings demonstrate a reliance on quantitative outcomes, with less attention to implementation processes. This limits insights into contextual adaptations and mechanisms through which integration succeeds or fails.

### Nature of integration and theoretical gaps

Most programs targeted primary school children This aligns with the importance of early child-focused interventions [90]. However, it underscores the need for multi-level approaches s [91–93].Despite global calls for whole-school approaches [94,95], majority of programs remain child-focused, with limited involvement of gatekeepers such as parents, school leadership, and the broader school community, that play a critical role in shaping policies and sustaining interventions [92,93,96].

Programs were primarily delivered by teachers, with external professional support in some cases. While central to school health promotion, there are concerns regarding teachers’ existing workload [97], suggesting that greater involvement of other school staff, integration within school curriculum, school environment restructuring, and community support may improve program sustainability.

Interventions included diverse program activities. Those that centred around changes in school policy, environment, or curriculum were limited, despite evidence that environmental and policy-level interventions yield long-term impact [98,99]. Notably, most programs employed multiple implementation strategies simultaneously, suggesting that multi-component approaches are preferred for school-based interventions.

Consistent with the CRF approach [24] and in alignment with integrated health promotion principles, most programs addressed unhealthy diets and poor hygiene. However, substance use, stress, and injury remain largely neglected in school health interventions, despite being widely recognized as critical shared determinants [23]. Even when diet and oral hygiene were incorporated, programs seldom addressed the full spectrum of CRFs as part of a comprehensive health promotion package. This suggests that CRF approach has not been fully operationalized, thereby limiting the breadth and potential impact of integrated school health programming. Consequently, valuable opportunities to address several overlapping determinants of oral and general health are being missed.

A striking gap identified in this review is the absence of frameworks guiding program design. Although some studies drew upon behavioural or educational theories, none explicitly applied integration- or implementation science-specific models or frameworks. This omission limits the ability to systematically design, implement, and evaluate interventions that merge oral health promotion with broader school health agendas. Furthermore, a theoretical grasp of how an intervention leads to change is essential [88,100], as it allows identification of gaps and aids in program improvement.

Most programs reported positive oral health outcomes, including improvements in children’s oral hygiene behaviours, dietary practices, and knowledge. Some demonstrated clinical benefits, while others highlighted program-related challenges, feedback, and content descriptions. However, outcomes related to parents, educators, and systemic change were less commonly reported. Long-term follow-up data were scarce, leaving the sustainability of interventions uncertain. Predominance of descriptive reporting across included studies limited cross-study comparison of implementation approaches.

### Strengthening school oral health integration

Findings from this review suggest several directions for strengthening the integration of oral health into school health programs. First, there is a need for rigorous and transparent reporting of study designs and methods, alongside qualitative research to capture contextual nuances. Second, future interventions should engage multiple interest holders, including families, school community, and policymakers, to ensure program sustainability and maximization of favourable outcomes. Third, greater emphasis should be placed on addressing a range of common risk factors, including substance use, stress, and injury, along with broader socio-economic and structural determinants, which remain underexplored. Most critically, researchers and practitioners should draw on appropriate implementation science frameworks including but not limited to Consolidated Framework for Implementation Research for examining contextual determinants [101], Expert Recommendations for Implementing Change taxonomy for classifying implementation strategies [102], and Implementation Outcomes Framework for evaluating implementation success [103]. These would to improve consistency, transparency, and reproducibility of evidence. These frameworks alongside ecological approach [104,105] and integration-specific models such as Rainbow Model of Integrated Care (reference), Singer’s model [16], or Fogarty’s model [106] may provide complementary guidance for designing, operationalizing, and evaluating integrated school-based oral health initiatives. Together, these strategies would enhance coherence across interventions, reduce duplication of efforts, and maximize limited resources.

### Strengths and limitations

This review is strengthened by its global scope and comprehensive methodology, including searches across six databases and reference lists of extracted studies. To the best of our knowledge, this study is the first scoping review to systematically map the integration of oral health into primary school health programs. However, several limitations warrant mention. Restricting inclusion to English-language publications may have led to omission of relevant evidence from non-English-speaking regions. Heterogeneity of program designs and reporting limited our ability to compare program effectiveness across studies. Reporting on program duration was inconsistent, and several studies failed to specify age of program population, further limiting comparability. Finally, the lack of integration or other relevant frameworks made it challenging to assess the fidelity of implementation.

The broad inclusion criteria may have captured programs in which oral health integration was incidental. To address variations in degree of integration, we developed an operational definition of integration and applied this consistently during full text screening and data extraction. Lastly, a risk of bias assessment was not conducted since the review was not intended to answer a narrow clinical question, evaluate intervention effectiveness, or assess the quality of included studies. Review findings should therefore be interpreted as mapping of available evidence rather than assessment of its methodological quality.

### Implications for research, policy, and practice

This scoping review underscores the importance of embedding oral health within broader school health initiatives rather than relying on siloed, short-term progress. It highlights that aligning oral health with broader school health priorities, guided by evidence-based multi-level strategies, and supported by sustainable policy and environmental change can drive its integration into school health programs. Study findings offer timely, actionable evidence to inform future research and policy as well as guide the design of integrated interventions and promote sustainable, equitable health outcomes. Furthermore, the study’s implications extend beyond oral health, offering a transferable model for embedding underprioritized health issues into existing multi-component school health strategies. Future research should examine system-level effects of integrated programs and incorporate cost-effectiveness analyses to better inform policy decisions. Use of well-established theoretical frameworks can strengthen program design, enhance replicability, and support scale-up across diverse contexts.

Strategic investment in teacher and support staff training, parental engagement, and creating a supportive school environment can help normalize oral health as a routine component of school health systems. Aligning oral health efforts with nutrition, hygiene, and health literacy initiatives can improve efficiency, reduce inequalities, and advance the overall well-being of children.

## Conclusions

Integrating oral health into existing school health structures, rather than creating standalone and resource-intensive programs, offers a more sustainable, equitable, and impactful pathway to improving both oral and general health outcomes among children. While oral health is included in primary school health programs, its integration is insufficiently grounded in theory and often limited in scope and duration. Future interventions could benefit from being explicitly guided by behavioural and implementation science theories, alongside ecological and integration frameworks, while engaging multiple interest holders, and evaluation of system-level and equity-related outcomes. Future school health initiatives should fully operationalize the CRF approach to address shared determinants of health, enhance program effectiveness and sustainability, and improve oral and general health outcomes for children. Greater attention to low- and lower middle-income settings is also needed, given the disproportionate burden of childhood oral disease in these contexts.

## Supporting information

S1 Appendix Completed PRISMA checklist. (PDF)

S2 Appendix Search strategy – Terms used on database search. (PDF)

S3 Appendix Codes and categories created to report extracted data. (PDF)

S4 Appendix Reasons for exclusion of studies during full-text screening. (PDF)

S5 Appendix Study aims, study population, and sample size. (PDF)

S6 Appendix Content and activities of general health programs and activities of oral health programs. (PDF)
