# Supplementary material for: Integration of oral health promotion in primary school health programs: A scoping review
Source: PLOS Glob Public Health. 2026 Oct 5;6(10):e0007390. doi: 10.1371/journal.pgph.0007390 (PMC13638057; doi:10.1371/journal.pgph.0007390)
Supplement: Supplementary file 2 [file pgph.0007390.s002.pdf]

S2 Appendix. Search Strategy – Terms Used on Database Search

Database	Search Format
Scopus<br>(n = 3,208)	*Search Date: February 6, 2026*<br>(TITLE-ABS-KEY((Health or wellness) W/4 (promotion or program* or project* or class* or unit or units or course or courses or education* or check-up* or awareness or screening* or intervention* or curricula or curriculum or subject or subjects)) AND TITLE-ABS-KEY(Oral-health or dental-health or oral-hygiene or dental-hygiene or dental-care or oral-wellness or dental-wellness or fluoride* or dental-sealant* or oral-check-up* or dental-check-up* or oral-screening* or dental-screening* or toothbrush* or toothpaste* or preventive-dentistry) AND TITLE-ABS-KEY(program* or project* or class or classes or unit or units or course or courses or screening or intervention* or curricula or curriculum or subject or subjects)) OR (TITLE-ABS-KEY(Oral-health or dental-health or oral-hygiene or dental-hygiene or dental-care or oral-wellness or dental-wellness or fluoride* or dental-sealant* or oral-check-up* or dental-check-up* or oral-screening* or dental-screening* or toothbrush* or toothpaste* or preventive-dentistry) AND TITLE-ABS-KEY(health-promoting-school*)) AND TITLE-ABS-KEY(School-based or schoolchildren or school-age* or preschool* or kindergarten* or kindergarten-school* or "nursery school*" or "early childhood education" or grade-one or grade-two or grade-three or grade-four or grade-five or grade-six or grade-seven or "grade 1" or "grade 2" or "grade 3" or "grade 4" or "grade 5" or "grade 6" or "grade 7" or "first grade*" or "second grade*" or "third grade*" or "fourth grade*" or "fifth grade*" or "sixth grade*" or "seventh grade*" or "primary school*" or "primary student*" or "primary teacher*" or "primary education" or "elementary school*" or "elementary ed*" or "elementary student*" or "elementary teacher*" or (school* W/5 ("k-7" or "k-6")))
Medline via Ovid<br>(n = 2,574)	*Ovid MEDLINE(R) ALL 1946 to February 05, 2026*<br>1 Health promotion/ or health education/ 145553<br>2 ((Health or wellness) adj4 (promotion or program* or project* or class* or unit or units or course or courses or education* or check-up* or awareness or screening* or intervention* or curricula or curriculum or subject or subjects)).mp. 495506<br>3 1 or 2 495506<br>4 Oral Health/ 23690<br>5 exp preventive dentistry/ or exp oral hygiene/ 38925<br>6 dental care/ or dental care for children/ 28257<br>7 (Oral health or dental health or oral hygiene or dental hygiene or dental care or oral wellness or dental wellness or fluoride* or dental sealant* or oral check-up* or dental check-up* or oral screening* or dental screening* or toothbrush* or toothpaste*).mp. 189399<br>8 4 or 5 or 6 or 7 200193<br>9 (program* or project* or class or classes or unit or units or course or courses or screening or intervention* or curricula or curriculum or subject or subjects).mp. 7450192<br>10 health promoting school*.mp. 523<br>11 (School-based or schoolchildren or school-age* or preschool* or kindergarten* or kindergarten school* or "nursery school*" or "early childhood education" or grade one or grade two or grade three or grade four or grade five or grade six or grade seven or "grade 1" or "grade 2" or "grade 3" or "grade 4" or "grade 5" or "grade 6" or "grade 7" or "first grade*" or "second grade*" or "third grade*" or "fourth grade*" or "fifth grade*" or "sixth grade*" or "seventh grade*" or "primary school*" or "primary student*" or "primary teacher*" or "primary education" or "elementary school*" or "elementary ed*" or "elementary student*" or "elementary teacher*" or (school* adj5 ("k-7" or "k-6"))).mp. 1272470<br>12 (11 and 3 and 8 and 9) or (8 and 10) **2574**

Embase via Ovid<br>(n = 2,576)	*Embase <1974 to 2026 February 04> (OVID interface)*<br>1 health promotion/ or health education/ 236022<br>2 ((Health or wellness) adj4 (promotion or program* or project* or class* or unit or units or course or courses or education* or check-up* or awareness or screening* or intervention* or curricula or curriculum or subject or subjects)).mp. 699494<br>3 1 or 2 699494<br>4 preventive dentistry/ 8832<br>5 mouth hygiene/ 39535<br>6 dental health/ 6298<br>7 (Oral health or dental health or oral hygiene or dental hygiene or dental care or oral wellness or dental wellness or fluoride* or dental sealant* or oral check-up* or dental check-up* or oral screening* or dental screening* or toothbrush* or toothpaste*).mp. 184476<br>8 4 or 5 or 6 or 7 203877<br>9 (program* or project* or class or classes or unit or units or course or courses or screening or intervention* or curricula or curriculum or subject or subjects).mp. 10939819<br>10 health promoting school*.mp. 612<br>11 (School-based or schoolchildren or school-age* or preschool* or kindergarten* or kindergarten school* or "nursery school*" or "early childhood education" or grade one or grade two or grade three or grade four or grade five or grade six or grade seven or "grade 1" or "grade 2" or "grade 3" or "grade 4" or "grade 5" or "grade 6" or "grade 7" or "first grade*" or "second grade*" or "third grade*" or "fourth grade*" or "fifth grade*" or "sixth grade*" or "seventh grade*" or "primary school*" or "primary student*" or "primary teacher*" or "primary education" or "elementary school*" or "elementary ed*" or "elementary student*" or "elementary teacher*" or (school* adj5 ("k-7" or "k-6"))).mp. 1071667<br>12 (11 and 3 and 8 and 9) or (8 and 10) 2654<br>13 limit 12 to conference abstracts 78<br>14 12 not 13 **2576**
Global Health<br>(n = 956)	*Global Health <1910 to 2026 Week 05> OVID interface*<br>1 health promotion/45920<br>2 health education/ 35404<br>3 ((Health or wellness) adj4 (promotion or program* or project* or class* or unit or units or course or courses or education* or check-up* or awareness or screening* or intervention* or curricula or curriculum or subject or subjects)).mp. 232436<br>4 1 or 2 or 3 232436<br>5 oral health/ or dental health/ 21038<br>6 dental hygiene/ 1485<br>7 preventive dentistry/ 235<br>8 (Oral health or dental health or oral hygiene or dental hygiene or dental care or oral wellness or dental wellness or fluoride* or dental sealant* or oral check-up* or dental check-up* or oral screening* or dental screening* or toothbrush* or toothpaste*).mp. 39733<br>9 5 or 6 or 7 or 8 39749<br>10 (program* or project* or class or classes or unit or units or course or courses or screening or intervention* or curricula or curriculum or subject or subjects).mp. 1738655<br>11 health promoting school*.mp. 504<br>12 (School-based or schoolchildren or school-age* or preschool* or kindergarten* or kindergarten school* or "nursery school*" or "early childhood education" or grade one or grade two or grade three or grade four or grade five or grade six or grade seven or "grade 1" or "grade 2" or "grade 3" or "grade 4" or "grade 5" or "grade 6" or "grade 7" or "first grade*" or "second grade*" or "third grade*" or "fourth grade*" or "fifth grade*" or "sixth grade*" or "seventh grade*" or "primary school*" or "primary student*" or "primary teacher*" or "primary education" or "elementary school*" or "elementary ed*" or "elementary student*" or "elementary teacher*" or (school* adj5 ("k-7" or "k-6"))).mp. 118549

	13 4 and 9 and 10 and 12 944<br>14 9 and 11 38<br>15 13 or 14 **956**
Eric<br>(n = 298)	*Search Date: February 6, 2026*<br>S1 ((Health or wellness) N4 (promotion or program* or project* or class* or unit or units or course or courses or education* or check-up* or awareness or screening* or intervention* or curricula or curriculum or subject or subjects))<br>S2 (Oral-health or dental-health or oral-hygiene or dental-hygiene or dental-care or oral-wellness or dental-wellness or fluoride* or dental-sealant* or oral-check-up* or dental-check-up* or oral-screening* or dental-screening* or toothbrush* or toothpaste* or preventive-dentistry)<br>S3 (program* or project* or class or classes or unit or units or course or courses or screening or intervention* or curricula or curriculum or subject or subjects)<br>S4 (health-promoting-school*)<br>S5 (School-based or schoolchildren or school-age* or preschool* or kindergarten* or kindergarten-school* or "nursery school*" or "early childhood education" or grade-one or grade-two or grade-three or grade-four or grade-five or grade-six or grade-seven or "grade 1" or "grade 2" or "grade 3" or "grade 4" or "grade 5" or "grade 6" or "grade 7" or "first grade*" or "second grade*" or "third grade*" or "fourth grade*" or "fifth grade*" or "sixth grade*" or "seventh grade*" or "primary school*" or "primary student*" or "primary teacher*" or "primary education" or "elementary school*" or "elementary ed*" or "elementary student*" or "elementary teacher*" or (school* N5 ("k-7" or "k-6")))<br>S6 (S1 AND S2 AND S3 AND S5) OR (S2 AND S4)
Education Research Complete (Ebscohost Interface)<br>(n = 109)	*Search Date: February 6, 2026*<br>S1 ((Health or wellness) N4 (promotion or program* or project* or class* or unit or units or course or courses or education* or check-up* or awareness or screening* or intervention* or curricula or curriculum or subject or subjects))<br>S2 (Oral-health or dental-health or oral-hygiene or dental-hygiene or dental-care or oral-wellness or dental-wellness or fluoride* or dental-sealant* or oral-check-up* or dental-check-up* or oral-screening* or dental-screening* or toothbrush* or toothpaste* or preventive-dentistry)<br>S3 (program* or project* or class or classes or unit or units or course or courses or screening or intervention* or curricula or curriculum or subject or subjects)<br>S4 (health-promoting-school*)<br>S5 (School-based or schoolchildren or school-age* or preschool* or kindergarten* or kindergarten-school* or "nursery school*" or "early childhood education" or grade-one or grade-two or grade-three or grade-four or grade-five or grade-six or grade-seven or "grade 1" or "grade 2" or "grade 3" or "grade 4" or "grade 5" or "grade 6" or "grade 7" or "first grade*" or "second grade*" or "third grade*" or "fourth grade*" or "fifth grade*" or "sixth grade*" or "seventh grade*" or "primary school*" or "primary student*" or "primary teacher*" or "primary education" or "elementary school*" or "elementary ed*" or "elementary student*" or "elementary teacher*" or (school* N5 ("k-7" or "k-6")))<br>S6 (S1 AND S2 AND S3 AND S5) OR (S2 AND S4)
