# Supplementary material for: Integration of oral health promotion in primary school health programs: A scoping review
Source: PLOS Glob Public Health. 2026 Oct 5;6(10):e0007390. doi: 10.1371/journal.pgph.0007390 (PMC13638057; doi:10.1371/journal.pgph.0007390)
Supplement: Supplementary file 3 [file pgph.0007390.s003.pdf]

S3 Appendix. Codes and Categories Created to Report Extracted Data

A. Contents / Topics Reported Related to General Health Promotion	Number of Studies	Content Related to General Health Category
Nutrition	25	Diet and nutrition
Healthy nutrition		
Healthy eating (nutrition)		
Healthy eating (nutrition)		
Healthy eating		
Healthy food choices		
Nutrition and healthy eating; Food hygiene; food safety, security, and supply; basic cooking skills		
Healthy foods		
Diet, sugar, and health		
Food health; Influence of nutrition on oral health		
Unhealthy diet / nutrition / healthy eating; Food safety		
Nutrition and food safety		
Healthy eating		
1. Unhealthy diet and nutrition (increased consumption of fruits, vegetables, water and unflavoured milk; decreased consumption of unhealthy foods such as sugary, starchy, and sticky foods, and sugary drinks)<br>2. Building self-efficacy (for engaging in healthy eating, drinking, mealtime, and snacking behaviors) and focus on subjective norms (healthy eating being a norm for other families with young children)		
Healthy food choices		
Nutrition		
Healthy nutrition		
Healthy nutrition and diet		
Nutrition and diet		
Nutrition, Obesity prevention, improving nutritional status, Important nutrients and their effects on body and related foods, Relation between nutrients and energy supply, Classification of regularly consumed food into different food groups ("food pyramid" and recommendations of healthy food)		
Healthy diet		
Healthy diet		
Types of traditional foods eaten by the Māori,		
Nutrition		
Healthy nutrition and cooking skills		
Handwashing (personal hygiene)	15	Hygiene
Hygiene		
Hygiene		
Personal hygiene (handwashing with soap and physical cleanliness); Menstrual hygiene		
Personal hygiene (handwashing techniques, nail hygiene, bathing practices, importance of using footwear)		
Personal hygiene		
Cleanliness		
Basic hygiene		
Handwashing (personal hygiene)		

Handwashing (personal hygiene)		
Personal hygiene		
Handwashing and deworming (hygiene)		
Personal hygiene		
Personal hygiene		
Hygiene		
Smoking	10	Substance Use and Addiction
Substance use and abuse		
Substance abuse		
Smoking and alcohol		
Tobacco use		
Tobacco control		
Anti-smoking and drug abuse control		
Smoking prevention		
Smoking prevention		
Addiction		
Environmental hygiene; climate change and global warming, emergency drills and trainings; recycling' resource preservation	5	Environment-Related & Science-Related Topics
Agriculture and recycling		
Agriculture and recycling		
Environmental hygiene		
Combined science outreach		
Mental health activities	7	Mental health
1. Positive thinking about health, self, and others<br>2. Develop positive attitudes, personal skills, and coping strategies<br>3. Body image, self-care, self-efficacy, self-esteem, and self-confidence<br>4. Diminish anxiety or less effective social responses<br>5. Goal setting to improve life, changing life, and control over life		
Mental health (illness understanding; communication skills, coping and seeking support; self-awareness; emotion, stress, and conflict management; stigma towards mental illness)		
Mental health and well-being		
1. Thinking positively about health, self, and others<br>2. Body image, self-esteem, self-confidence, self-efficacy, self-care, goal-setting, changing life, control over life, improving personal skills<br>3. Diminish anxiety or less effective social responses, develop positive attitudes and coping strategies		
Stress management		
Mental health		
Healthy recreation (activities and leisure time)	8	Physical activity
Exercise behavior		
Importance of physical activities		
Physical activity		
Physical activity / active living		
Physical activity		
Physical activity		
Physical activity		
Personal safety and injury prevention	7	Injury Prevention
Injury prevention and first-aid treatment		
Trauma and violence		
Personal safety and injury prevention		

Dental trauma prevention		
First aid		
Safety and injury prevention; First-aid		
Bullying prevention	6	Abuse (includes sexual abuse and bullying)
Sexual abuse		
Sexual abuse		
Bullying and exclusion		
Child abuse prevention		
Bullying		
Security	4	General Safety
Online privacy		
Safe work		
Safety		
Behavior skills	4	Topics Related to Behavior Change
Building self-efficacy (for engaging in healthy eating, drinking, mealtime, and snacking behaviors) and focus on subjective norms (healthy eating being a norm for other families with young children)		
Behavioural rehearsal (practising healthy behaviors)		
Behavior rehearsal (practising healthy behaviors)		
Vaccination and dealing with pain	14	Other Health Issues (includes vaccination / immunization, pain, sleep, adolescent health, HIV/AIDS, infectious diseases, human body, deworming)
Child health and development (developmental milestones and child management techniques (Immunization (purpose, diseases which immunization can prevent, when baby should be immunized)		
Chronic pain prevention; vaccination		
Sleep		
Sleep schedule and screen time		
Sex education		
HIV / AIDS and teenage pregnancy		
HIV / AIDS		
Infectious diseases		
Infectious disease and endemic disease prevention; Healthy lifestyles and teenagers		
Healthy growth and development		
Human anatomy and physiology		
Deworming and iron supplementation		
Cardiovascular health; Anatomy and physiology; Knowledge of hypertension		
Health literacy	6	Concept of Health & Disease Prevention
Health literacy		
Health awareness; Health as a continuum; Problem prevention, symptom reduction, improving and maintaining good health; acceptance of heart or oral diseases		
Preventing problems, reducing symptoms, improving and maintaining good health; Health as a continuum (not just sick/well) and examples and being happy despite having diseases		
General health		
Concept of health and causes of disease		

B. Contents / Topics Reported Related to Oral Health Promotion	Number of Studies	Content Related to Oral Health Category
Unhealthy diet and nutrition (Tooth-healthy and unhealthy foods and beverages - sugary, starchy, sticky); Building self-efficacy (for healthy eating, drinking, mealtime, and snacking behaviors) and focus on subjective norms	19	Diet and nutrition
Unhealthy diet / nutrition (foods high in fibre, fat, sugar, salt, minerals); Usual breakfast and favourite foods		
Unhealthy diet / nutrition		
Healthy nutrition		
Nutrition and diet		
Cariogenic impact of sugar- and carbohydrate-containing foods; Recognise frequency of consuming cariogenic snacks		
Sugar and sugary drinks and their effects, Identify hidden sugars in sugary drinks and foods,		
Diet, sugar, and health (general and dental)		
Influence of nutrition on oral health		
Healthy foods		
Healthy food choices		
Healthy food choices		
Healthy eating (nutrition)		
Healthy eating (nutrition)		
Healthy eating		
Healthy diet (healthier snacking habits in both the school and the wider environment)		
Healthy diet (good and bad foods for health), care of teeth		
Healthy diet		
Oral health education (no sugar, no alcohol, no smoking)		
Maintaining oral health	24	Oral hygiene, dental health, dental care, methods of prevention
Oral health, oral health check-ups		
Care of teeth		
Dental care		
Dental care		
Dental health		
Dental health and oral hygiene		
Oral hygiene		
Oral hygiene		
Oral hygiene		
Group toothbrushing (dental hygiene)		
Tooth brushing (oral hygiene)		
Toothbrushing (oral hygiene)		
Toothbrushing (oral hygiene)		
Toothbrushing (oral hygiene)		
Toothbrushing		
Brushing teeth effectively under supervision of teachers and researchers		
Tooth paste use, brushing and fluoride use (oral hygiene); Dental caries prevention and management		
Toothbrushing, fluoride mouthrinse, fissure sealant		
Toothbrushing; Other preventive treatments (rarely)		
Regular dental care and visits, oral hygiene, toothbrushing, and flossing		
Caries prevention methods (use of fluoride toothpaste, brushing technique and		

brushing frequency)		
How can we protect our teeth? How fissure sealants and fluoride can protect our teeth, Brushing frequency and technique		
Self-care for oral health, importance of dental visits		
Role of dental plaque in caries	6	Oral Diseases & Related Concepts
Human anatomy and physiology		
Oral health; Teeth and their functions; Dental plaque and tooth decay		
Healthy mouth, Inevitability of oral diseases, Health as a continuum, Satisfaction rating related to oral health and how to improve it, Acceptance of heart or oral diseases		
Microorganisms in body and mouth, Effect of acid on teeth		
Oral health and healthy mouth; Inevitability of oral diseases and health as a continuum		
Tobacco use prevention	5	Substance Use (Includes Tobacco & Alcohol)
Substance use and abuse		
Smoking		
Anti-smoking		
Oral health education (no sugar, no alcohol, no smoking)		
Thinking positively about health, self, others, and condition of mouth; Good body image, self-efficacy, and self-confidence	3	Skill Building
Positive thinking about health, self, others, and mouths; Body image, self-care, self-efficacy, self-esteem, and self-confidence; Satisfaction rating related to oral health and how to improve it		
Building self-efficacy (for healthy eating, drinking, mealtime, and snacking behaviors) and focus on subjective norms		
Injury prevention	3	Injury prevention
Injury prevention		
Dental trauma and injury prevention		
Dental anxiety	1	Stress

C. Activities Implemented Related to General Health Promotion	Number of Studies	General Health Activities Category
Activities delivered by schoolteachers	16	Activity-Based Learning
Organise health quiz, drawing, essay writing, competitions; campaigns; supervise hand washing and toothbrushing; conduct DM Club activities on co-curricular days; engage in outside school activities		
Cooking traditional local recipes; Drawing, interactive games, puzzles; Daily monitoring of handwashing and oral hygiene by teachers		
"Course review horay method" (testing understanding of presented material, through direct questions or demonstrations); Group formation; Practice of washing hands and brushing teeth observed by facilitators		
Healthy school projects through brainstorming, planning, implementation, and evaluation with teacher support -suggesting to others to choose healthy foods; Games, mask making, role play, poster making (classroom activities); Working with teachers to run a healthy snack shop and children determined that confectionary would not be sold in school		
Working to run a healthy snack shop and children determined that confectionary would not be sold in school; Teacher-supported healthy school projects (brainstorming, planning, implementation, and evaluation) such as suggesting to others to choose healthy foods; Classroom activities (mask making, role play, games, poster making)		
Classroom activities to enable children to recognize and choose healthy foods (measuring portions of food, reading food labels, identify and compare nutrient content of common foods); Students keeping a log book to monitor fitness level and exercise plans; Physical education program / aerobic fitness classes; Role plays		
Teacher-led workshops and story-telling; Teacher-hosted cooking workshops with rare and lesser-known healthy ingredients; Workshops offered by community organization (on online privacy, legal and criminal aspects of non-consensual distribution of intimate images); Students take turns pedalling on a stationary bicycle for 20 minutes during normal class activities (kilometres compiled and accumulated for entire school); Trip to the shore to pick up cigarette butts; Nurse and social worker partner to provide workshops on health and environmental impacts of tobacco		
Hands-on experiments and educational, sensory, cooking, and molecular gastronomy workshops; Group activities, educational games, story, description, demonstration, observation, brainstorming, simulation		
Perform physical and cultural activities from Monday to Friday for three hours daily; Workshops on food, nutrition, and hygiene habits; Perform general and oral hygiene practices (supervised tooth brushing)		
Learning stations (to taste and smell different fruits and vegetables, and determine sugar and fat contents of sweets, snacks and beverages); Learning about food shopping and how to prepare healthy breakfast; "Fitness Pyramid" (to reflect student's physical activity and show ways to increase physical activity); Competitions on vaccination, quiz on pain, active games; Other activities (cartoons and pictorial illustrations, role-plays)		
Group discussions, role play, demonstrations, drama, poster competition, toy making from waste		
Daily group handwashing with soap		
Daily supervised handwashing with soap and clean water; Daily tasks implemented by teachers		
Children performed daily group handwashing with soap		
Several structured physical activities (integrating messages on nutrition, oral health education, and dental trauma prevention)		

Community involvement in school health	13	Empowerment and Advocacy (children, teachers, parents, community)
Parents were informed about activities		
Committee consisting of school health and dental services representatives was established to form a multidisciplinary working group consisting of a physician, a dentist, a dietician and some educators		
Formal school initiatives to promote healthy eating (within management, teachers, community involvement, teacher and parent education, family engagement, providing healthy food and snacks, menu by nutritionist);		
Implementation of written school policy, co-developed in partnership among researchers, practitioners, school community (dieticians, school meal advisors, teachers, health promotion officers, local dairy suppliers of school milk), parents, and, governors of school and preschool groups, and approved by Board of Governors - allowing only consumption of milk and fruit at break-time and schools must agree not to sell snacks high in fat or sugar in school; Awareness building and negotiation; Teachers must agree not to reward students with candy' Facilitate healthier eating by empowering children to make healthier choices		
Voluntary community involvement in school health programs		
Canteen operators' participation, demonstrations of WASH-related daily good practices with school communities, nutrition-related good practices to teachers, caregivers/parents, canteen operators; PTA meetings and engagement of caregivers/parents for food preferences, education, weekly menu, giving printed recipes, cooking food with local ingredients, package and prepare meals; At-home activities; 6 modules delivered by NGO staff; Advocacy with administration, involvement of ministries, school system engagement through audits; Observation of daily meals packed by caregivers for children		
All parents must be informed in writing of the school's intention to adopt a healthy snacks policy and the benefits to the school		
Parent education (family health) through newsletter delivering information and recipes to families and siblings of Heart Smart students; Parents participation facilitated through COR (Heart) Committee composed of parents and teachers; Food service workers in cafeteria learn principles and procedures for reducing salt, sugar, and fat in recipes; Behavioural pledges		
Assurance from teachers that confectionery, cakes, biscuits or sugar-sweetened soft drinks will not be given as rewards or prizes		
Children receive nutritional counselling to prevent obesity and health education		
Children determine that confectionary would not be sold in schools; Projects involved whole school and all students and staff participated		
Children determined that confectionery would not be sold in school; Participation of whole schools, students, and staff		
Health promotion, education, awareness, and basic health messages, visits by school health nurses to talk to students	13	Classroom Session, Discussions, and Instructions
Classroom instructions by trained teachers		
Health education		
Classroom instruction (modules)		
Health education (cooperative learning model); Children explained about handwashing and toothbrushing through power point presentations with opportunity to ask about related material; Showing hand washing and toothbrushing video for ± 15 minutes		
Teacher-delivered sessions (1 to 2 hours) developed with children, didactic teaching, discussion, and in-class presentations		
Teacher led in-class conversations on bullying; School visits by community-based organization to talk to children about how to safely travel from home to school on their bicycle		

Nurse-delivered awareness campaign of 40-45 minutes; Lectures, power point presentations, and live demonstrations (whiteboard, drawings) with content taken from WHO AND UNICEF		
6 modules led by NGO-staff; Teacher-led in-school discussions		
Teaching activities by teachers		
Class teachers assisted by principal researcher to implement an action-oriented child friendly curriculum (1 hour of learning each week of the school year = 30 sessions over 3 terms)		
Didactic teaching, weekly teacher-delivered sessions (co-developed with children), discussion, and in-class presentations		
A video introducing group hygiene activities		
Children receive healthy meals	9	School Nutrition Policy and Environment Changes
Organization of cost-effective school feeding program		
Implementation of written school policy, allowing only consumption of milk and fruit at break-time and schools must agree not to sell snacks high in fat or sugar in school		
Formal school initiatives to promote healthy eating (within management, teachers, community involvement, teacher and parent education, family engagement, providing healthy food and snacks, menu by nutritionist); Favourable school environment i.e. no convenience or fast-food store within 500 m of the school		
Promotion of healthy food through healthier lunches and sale of healthy foods in some schools		
Promotion of healthy food in schools		
Written healthy snack policy; During mid-morning breaktime, only milk/water and/or fruit/vegetables should be allowed; Smart snacks scheme (which runs annually); All parents must be informed in writing of the school's intention to adopt a healthy snacks policy and the benefits to the school		
Implementation of school policy allowing only consumption of milk, water, vegetables, and fresh fruit at school break-times and closing of tuck shops		
Healthy school lunches		
Orientation and supervision by school health nurses	9	Provision of Support and Supervision
Technical support		
Committee consisting of school health and dental services representatives was established to form a multidisciplinary working group consisting of a physician, a dentist, a dietician and some educators		
National DMP (Doktor Muda Program) convention held every 2 years to allow idea exchange and promote best practices		
Incentives and supportive leadership		
Direct observation of program sessions by trained project staff; Social support for parents, teachers, and children (to serve and consume fruits, vegetables, water, and unflavoured milk)		
Researcher support to deliver activities, supportive leadership, and incentives		
Class teachers assisted by principal researcher to implement an action-oriented child friendly curriculum (1 hour of learning each week of the school year = 30 sessions over 3 terms)		
Monitoring of school activity by Community practitioners and Community Dental Service for difficulties, policy compliance and adherence		
Regular government health education curriculum	8	Curriculum Inclusion
Health promotion in school curricula		
Health education as part of regular school curriculum		
Health education in regular lectures in subject biology, physical education and math, and in framework of special project days		
Inclusion of health-related topics in school curriculum		
Health messages in curriculum		
Health messaging forming a part of the curriculum		

2 supplementary theoretical activities on hygiene/maintaining oral health and influence of nutrition on oral health - 50 minutes each were introduced in chapter 'Personal Hygiene' and 'Food Health' and Optional subject called 'Education for Health' (EH) added to curriculum		
Demonstrations of WASH-related daily good practices with school communities, nutrition-related good practices to teachers	7	Teacher Training
Teacher training and use of teachers' guides		
Teachers trained by researcher and provided session handbook		
Teachers trained by researcher, provided handbook, with researcher support to deliver activities		
Use of train-the-trainer model (to train schools and their staff in program implementation)		
Teachers trained to implement GeKoKidS program and received a booklet containing presentation slides and teaching materials		
Educational staff received practical guidance and training materials, but no further support to implement programme activities		
Put up posters	7	Use of Educational Materials
Information, wall posters, and learning materials; Educational material for use by school teachers, parents, health service workers		
Bilingual and culturally appropriate parent engagement materials		
Stickers for display near toilet and hand washing facilities		
Use of student activity workbooks		
Use of posters and educational materials		
Worksheets on dental health and vaccination		
Undertake health campaigns at community elderly centre	7	Events and Campaigns
Health fairs		
Whole school assembly		
Haque city council spent 200,000 guilders per year for a long-term dental health campaign		
End-of-year open day		
Events		
Events		
“Fitness Olympics” (application of a test for assessing children's and adolescents' motor skills)	6	Screenings and Service Provision
Annual provision of school health services and screenings by Department of Health		
Volunteer-assisted periodic risk factor screenings for staff and students, periodic assessments of body composition, and fitness tests		
School health services and annual screenings by Department of Health		
Biannual deworming with single dose of albendazole (400 mg); Annual physical examination		
Biannual deworming as part of respective national government-coordinated deworming programme		
Distribution of single (10-ml) commercial toothpaste sachet, a toothbrush,	6	Resource Provision
Technical support and supplies such as soap, cleaning rotas, toilet user kits, cleaning products, and a manual provided; One basic ready-to-install handwashing facility per classroom; Stickers for display near toilet and handwashing facilities		
Construction and maintenance of group washing facilities		
CATCH Healthy Smiles coordination kit (resources and instructions to school administration to integrate and coordinate program activities for simultaneous implementation across the school); Digital CATCH online platform to access program resources for all schools		
Toothbrushes and fluoridated toothpastes provided by schools to children		
Donation of resources; WASH equipment and disposable material		
Group of children (DM) selected and trained by trained teachers through modular curriculum, for peer education and empowerment, and to act as role models. DM conducted	4	Peer Support

health talks; leaflet distribution; prepare health messages; put up posters; organise health quiz, drawing, essay writing, competitions; campaigns; supervise hand washing and toothbrushing; monitor peers; keep record of health problems; provide first aid; help teachers to record student’s health report card for weight, height, eye test, health inspection and personal hygiene on school health day; conduct DM Club activities on co-curricular days; engage in outside school activities, and undertake health campaigns at community elderly centre
Invitation of other classmates to a breakfast
Development of peer support systems
Teaching peers how to brush their teeth effectively

D. Activities Implemented Related to Oral Health Promotion	Number of Studies	Oral Health Activities Category
Children determined that confectionery would not be sold in schools	13	Interest Holder Engagement, Advocacy, and Empowerment
Children determined that confectionery would not be sold in schools; Suggesting to others to choose healthy foods		
Assurance from teachers that confectionery, cakes, biscuits or sugar-sweetened soft drinks will not be given as rewards or prizes		
Facilitate healthier eating by empowering children to make healthier choices; School break-time policy (Boosting Better Breaks - BBB), developed in partnership among researchers, practitioners, and school community (team of dieticians, school meal advisors, teachers, health promotion officers, and local dairy suppliers of school milk); Awareness building, negotiation, and co-development of policy with parents, teachers, and school governors and pre-school groups within school environment; Teachers must agree not to reward students with candy		
Awareness for learners, educators, and parents; Incorporation of oral health education into parent meetings		
Committee consisting of school health and dental services representatives was established to form a multidisciplinary working group consisting of a physician, a dentist, a dietician and some educators		
School principal, classroom teachers, and support staff involvement in translating concepts from English to Māori and vice-versa, and ensuring activities delivered with school's cultural values		
Social support for parents, teachers, and children (to serve and consume fruits, vegetables, water, and unflavoured milk); Bilingual and culturally appropriate parent engagement materials (program website resources, interactive parent-child ''homework activities,'' infographics, CATCH family fun nights in schools)		
Written information sent to parents about school snack policy		
Children and parents informed about examination results; Reduction in consumption of sugar-sweetened beverages; Prizes for classes with best caries status		
Parents were informed about activities		
Mothers informed of dental care of children		
Parent education (family health) through newsletter delivering information and recipes to families and siblings of Heart Smart students, Behavioural pledges, Parents participation facilitated through COR (Heart) Committee composed of parents and teachers; Food service workers in cafeteria learn principles and procedures for reducing salt, sugar, and fat in recipes		
Oral health education	14	Oral Health Education, Discussions, and Instructions
Education		
Dental hygiene education		
Nurse delivered awareness sessions; Lectures; Power point presentations; and Live demonstrations (whiteboard, drawings)		
Educational sessions by trained teachers.		
Classroom instructions by trained teachers		
Classroom instruction (modules)		
Received oral health education offered sporadically during workshops on food, nutrition, and hygiene habits; Taught tooth brushing technique by trained supervisor and teachers		
Weekly visits by nurses in one school to advise children; Awareness for learners; Those that had knowledge of oral health would incorporate this into their teaching; Brushing instructions		
Health education (cooperative learning model); Children explained about handwashing and toothbrushing through power point presentations with opportunity to ask about related material; Showing hand washing and toothbrushing video for ± 15 minutes; "Course review horay method" (testing understanding of presented material, through direct questions or demonstrations)		
Educational sessions by teachers		
Class teachers assisted by principal researcher to implement an action-oriented child friendly		

curriculum (1 hour of learning each week of the school year i.e. 30 sessions over 3 terms)		
In-class presentations to students on proper teeth brushing technique		
Video showing group hygiene activities		
Supervised daily toothbrushing	12	Structured Toothbrushing
Performed daily supervised toothbrushing (10-minutes) after meals using fluoridated toothpaste and toothbrush		
Daily supervised toothbrushing with fluoride toothpaste by children		
Supervised toothbrushing with fluoride toothpaste (0.3 ml; 1,450 ppm)		
Daily group toothbrushing with 0.3 ml of toothpaste (containing 1450 ppm free available fluoride)		
Teacher-led activities, included daily monitoring of handwashing and oral hygiene by teachers		
Weekly toothbrushing with fluoride toothpaste (under supervision)		
Tooth brushing under supervision of teachers and researcher		
Regular toothbrushing in schools		
Practice of washing hands and brushing teeth observed by facilitators		
Children's participation in daily oral hygiene instructions in presence of mothers and supervised by teachers		
Toothbrushing program (daily in 8 schools and 2-3 times per week in 4 schools)		
At-home activities; drawing, interactive games, and puzzles	11	Experiential Learning (workshops and group activities)
Learning activities (Role play, Group discussions, Drama, Poster competitions, Toy making from waste household materials)		
Group formation		
Group activities and healthy school projects (suggesting to others to choose healthy foods, working with teachers to run a healthy snack shop		
Group activities and projects; Working with teachers to run a healthy snack shop		
Competitions; Team activities; Creating healthy breakfast; Measuring sugar in various foods; Preparing poster; Hands-on sessions; Demonstrations / science experiments; 6 Interactive activities conducted during school hours in English; ensuring activities delivered with school's cultural values		
Prizes for classes with best caries status		
Nurse and social worker partner to provide workshops on health and environmental impacts of tobacco; Trip to the shore to pick up cigarette butts		
Students use a nutrition schedule to fill beverage and food impulses and schedule analysed by teacher and/or dentist and Worksheets for calculations of caries prevalence		
Use of student activity books; Classroom activities to enable children to recognize and choose healthy foods (measuring portions of food, reading food labels, identify and compare nutrient content of common foods); Health fairs		
Several structured physical activities (integrating messages on nutrition, oral health education, and dental trauma prevention)		
Direct observation of program sessions by trained project staff; CATCH Healthy Smiles coordination kit (resources and instructions to school administration to integrate and coordinate program activities for simultaneously implementation across the school); Digital CATCH online platform (to access program resources for all schools - http://catchinfo.org/)	9	Monitoring and Professional Support
Educational staff received practical guidance and training support		
Some schools had intervention support from Colgate World of Care and provincial and Department of Health (not continuous and dependent on availability of supplies); Mobile messenger application set up between researcher and school health teams for ease of communication and sharing ideas between schools		
Community practitioners and Community Dental Service monitor school activity, keeping an eye out for difficulties encountered and policy compliance and adherence		
Supervision by trained and calibrated students and staff from dentistry, oral health and human nutrition		

Class teachers assisted by principal researcher to implement an action-oriented child friendly curriculum (1 hour of learning each week of the school year i.e. 30 sessions over 3 terms)		
Feedback to teachers about caries status, vaccination rates, weight distribution, smoking habits and school meals		
Surveillance visits by a supervisor		
School management team was requested for a scientific counsellor		
Practical intervention (visit to a dental office volunteered by their biology teacher and the dentist - professional evaluation of oral health and professional brushing)	9	Dental Screenings and Service Provision
Professional visits		
Check-ups and visits		
Oral health check-ups		
Service provision (not regular)		
Dental hygienist visits for school-wide dental screening		
Students motivated to participate in biannual, free of charge dentist screening		
Fluoride mouthrinse and fissure sealant programs (inconsistent frequency); Colgate World of Care visits once a year; School visits by oral health personnel when supplies were available		
Other caries control interventions (tooth extraction, abscess drainage, medications, and brushing with fluoride gel		
Teacher training; Use of teachers' guides	6	Teacher Training
Educational staff received practical guidance and training support		
Awareness of educators		
Teacher education		
Classroom instructions by trained teachers		
Use of train-the-trainer model (to train schools and their staff in program implementation)		
Use of educational materials (clean water, toothbrush, paste, dental floss, game puzzles, apples, samples of local crops)	5	Use of Educational Materials
Stickers for display near toilet and handwashing facilities		
Since 1972 the 6-9-year-old children and since 1974 the 9-11-year-old children in elementary schools were reached by information, wall posters, and learning materials; Group developed educational material for use by school teachers, parents, health service workers		
Parent education (family health) through newsletter delivering information and recipes to families and siblings of Heart Smart student		
Bilingual and culturally appropriate parent engagement materials (program website resources, interactive parent-child ‘‘homework activities,’’ infographics, CATCH family fun nights in schools)		
Healthy school lunches	5	School Nutrition Policy and Environment Changes
Sugary snacks control by vendors and tuck shops; Promotion of healthier lunches		
Introduction of healthy foods at school break-times (milk, water, vegetables, and fresh fruit); Closing of tuck shops		
Written healthy snacks policy; Only milk, water, fruits, and vegetables allowed during breaktime; Written information sent to parents about school snack policy		
Implementation of school policy allowing only milk and fruit consumption at break-time; School break-time policy (Boosting Better Breaks - BBB), developed in partnership among researchers, practitioners, and school community (team of dieticians, school meal advisors, teachers, health promotion officers, and local dairy suppliers of school milk); Schools must agree not to sell snacks high in fat or sugar in school environment		
Inclusion of oral health-related topics in school curriculum	5	Curriculum Inclusion/Restructure
Oral health education in regular lectures in the subject biology and math		
Optional subject called 'Education for Health' (EH) added to curriculum - 2 supplementary theoretical activities on hygiene/maintaining oral health and influence of nutrition on oral health - 50 minutes each were introduced in chapter 'Personal Hygiene' and 'Food Health'		

Incorporation of oral health education into school curriculum and parent meetings		
Classroom curriculum for oral health education		
Provision of toothbrushes and fluoridated toothpastes by schools to children	5	Provision of Supplies
Toothbrushes and fluoridated toothpastes provided by schools to children		
Toothpaste and technical support provided		
Construction and maintenance of group washing facilities		
Toothbrushes, 3-month supply of toothpaste, and brushing instructions provided - program discontinued once supplies depleted; Outsourcing of supplies		
Anti-smoking campaign	4	Events and Campaigns
Haque city council spent 200,000 guilders per year for a long-term dental health campaign		
End-of-year open day		
Health fairs		
Older students supporting younger students	3	Student Peer Support
Teaching peers how to brush their teeth effectively		
Teaching effective toothbrushing to peers		

E. Program Implementation Strategies	Number of Studies	Strategy Category
By building health-related skills through group activities/projects<br>By building health-related skills through classroom instruction<br>By building health-related skills through supervised actions<br>Student empowerment	25	Student Skill Building and Empowerment
Through teacher involvement<br>Through teachers' training<br>Through didactic materials for teachers	19	Teacher-Focused Strategies
Through school engagement<br>Principal involvement<br>Involvement of school canteen<br>Parent engagement<br>Involvement of PTA<br>Community partnerships<br>Political involvement	16	Interest Holder Involvement
Through changes in physical and/or social environment<br>Through school policy	13	Changes in School Structure and Policies
Through other strategies such as whole school approach	11	Other Strategies
By improving access to resources<br>By making products available	6	Resource-Based Approach
Through making oral health part of existing curriculum<br>Through curriculum redesign / restructuring	5	Curriculum Inclusion
Through professional supervision of activities	5	Professional Support

F. Oral Health-Related Outcomes Reported	Number of Studies	Outcome Category
1. School environment promoting healthy eating and incorporates socio-environmental and policy aspects of health promotion may be effective in reducing dental caries in elementary school children.<br>2. Three distinct school environment types were identified: Type 1 and 2 schools had strong healthy eating programs, whereas Type 3 had weak programs. Type 1 schools had favourable neighbourhood food environments, whereas Type 2 and 3 had unfavourable ones.<br>3. Adjusting for potential confounders, children attending Type 1 and 2 schools had 21% (incidence rate ratio¼0.79, 95% CI¼0.68, 0.90) and 6% (incidence rate ratio¼0.94, 95% CI¼0.83,1.07) lower 2-year incidence of dental caries, respectively, compared with Type 3 schools.	13	Children's Oral Health Outcomes *(increase in caries-free and plaque-free status; reduced decayed, missing, and filled teeth)*
Children in intervention schools in all three countries had lower increment (average of 24%) in number of decayed, missing, and filled permanent teeth between baseline and follow-up, in comparison to children in controls schools		
1. Significantly higher increase in mean BMI and lower prevalence of moderate to heavy soil-transmitted helminths infection than control group (after year 1)<br>2. No changes in prevalence of caries and dental infections across groups		
1. In 7- and 9-year-olds, significant dsfs reductions of 32.5 and 25.6% in 1978 were found (Pt<0.001)<br>2. Percentage of caries free children increased in all age groups over the last nine years (d3mfs = 0; D3MFS = 0)<br>3. Percentages of caries free children were 52%, 21%, and 7% for 7-, 9- and 11- year age groups, respectively		
1. Significant increase in DMFT between year one and year two in children in BBB schools<br>2. Moderate increase in mean number of sound permanent teeth for children attending BBB participating schools (year one: 12.44 [95% CI 11.46, 13.40]: year two: 14.71 [95% CI 13.49, 15.99]) compared with those attending control schools (year one: 13.75 [95% CI 12.84, 14.65]: year two: 14.65 [95% CI 13.52, 15.80]) (F [1,85] = 3.66; p < 0.05 [one-tail test])<br>3. Significant increases in mean DMFT (F[1,85] = 22.01; p < 0.001), mean number of missing teeth (F[1,85] = 4.17; p < 0.05), and mean number of filled teeth (F[1,85] = 22.98; p < 0.001) between year one and year two. Moderate increase in mean number of sound permanent teeth for children attending BBB participating schools (year one: 12.44 [95% CI 11.46, 13.40]:year two: 14.71 [95% CI 13.49, 15.99]) compared with those attending control schools (year one: 13.75 [95% CI 12.84, 14.65]: year two: 14.65 [95% CI 13.52, 15.80])(F[1,85] = 3.66; p < 0.05 [one-tail test])		
1. A 35% higher caries risk was observed in the control group<br>2. However, parents' socioeconomic characteristics modified the effect of the program on their children, as high socio-economic status in the intervention group was associated with 94% reduction in the incidence risk ratio (p<0.001)		
Mean percentage differences for plaque-free surfaces (8.8±28.5%) and healthy gingival units (23.3%±23.2%) were significantly higher in the intervention group		
Mean increments in f-s was 0.33 and 0.06 and of F-S was 0.16 and 0.03 in experimental and control groups respectively		
Only 27% students were caries free and 90% required preventive care		
1. Intervention group had lower scores on Child Perception Questionnaire (CPQ), representing better oral health related quality of life, accounting for 6.07 CPQ points at 2 weeks post-intervention and 6.50 CPQ points at 3-months post intervention<br>2. Intervention group had sense of coherence points 6.39 higher than those of the controls at 2		

weeks post-intervention and 4.05 at 3-months post-intervention<br>3. More children in intervention group had normal gingivae (31.81%) than the controls (19.51%) [χ^2 (1) = 4.41, p = 0.04] at 3-months post-intervention		
Significantly lower proportion of intervention children reported bleeding gums (31.4%) and presence of plaque or calculus (7.5%) as causes of oral impacts compared to control group, respectively (35.5%, p = 0.016; 9.7%, p = 0.032)		
Handwashing and toothbrushing behaviours and plaque scores did not significantly differ between intervention and control schools, four-months post-intervention		
1. Participating schools had significantly lower percentage of children with > 2 decayed teeth (t_{239}=4.08, <0.001) and requiring urgent dental treatment (t_{241}=2.96, p=0.004)<br>2. Schools participating in 3 or > health-related activities had significantly lower percentage of children requiring urgent dental treatment (F_2=7.04, p<0.001) and children with > 2 decayed teeth than schools with 1-2 health-related activities or no health promotion initiatives (F_2=4.56, p=0.008)<br>3. Lower percentage of children in low-income participating "Healthy Schools" required preventive care, had non-urgent treatment needs, had urgent treatment needs, and > 2 decayed teeth than in low-income non-participating schools (F_2=21.7, p<0.001); (F_2=9.79, p<0.001); (F_2=10.86, p<0.001); and (F_2=12.04, p<0.001) respectively		
Decay into dentine at follow-up was predicted by school intervention status and evidence of sugar snacks consumption outside school and at home		
Handwashing and toothbrushing behaviours and plaque scores did not significantly differ between intervention and control schools, four-months post-intervention.	10	Children's Behavioural Outcomes *(improvement in oral hygiene habits, toothbrushing behaviors, toothpaste use, use of fluoride tablets, dental visits, dietary habits/food choices, and healthy lifestyle)*
Hand hygiene and tooth brushing practices increased from 0% (pre-test) to 92% (post-test) and from 3% (pre-test) to 100% (post-test) respectively, with no changes in control group		
Positive changes in students' nutritional behaviours, hygiene practices, and life skills		
1. Practice overall scores of healthy lifestyles increased significantly in 8-year-old students (34.09% improvement) and 11-year-old students (30.21% improvement)<br>2. Factors such as governorate, normal-weight status, and close supervision influenced improvement		
1. 60% children at baseline and all children at follow-up had at least one sugar snack in their rubbish bag<br>2. 47% children at baseline visited shops around school hours, increasing to 63% at 24-month follow-up, with more frequent snack-related rubbish found among those stopping on the way home from school<br>3. Initially, children in policy schools consumed healthier snacks (milk and fruit), while control school children had more chocolate and crisps, but by 24 months, both groups showed increased consumption of less healthy snacks (chocolate, crisps, and sugary drinks)<br>4. In school environment, intervention group children had significantly lower mean scores for sugar snacks scores at baseline but equivalent mean sugar snacks scores at follow-up compared with children attending control schools<br>5. In outside school environment, there was no effect of school intervention on sugar snack scores		
1. Fluoride tablets use increased in each age group between 1972 and 1975, especially in 9- and 11-year-old children<br>2. However, in 1978 a decrease was found as compared with 1975.		
1. Children's twice-a-day toothbrushing and dental visits were significantly higher in experimental group (26% and 10%) compared to control group (19% and 3%)<br>2. Increments in fluoride toothpaste use among children was significantly higher in experimental group (11%) compared to control group (5%)		

3. Frequency of eating cakes and biscuits declined by 5% in experimental group compared to increase by 5% in control group		
1. Food choices and handwashing improved significantly - overall scores shifted from 0.83 to 0.87 (P=0.001) and from 0.57 to 0.68 (P<0.001) respectively (with boys handwashing being more competent compared to girls)<br>2. Children over 9 years significantly benefited from the course (increased knowledge, attitude, practices, food choices, handwashing), with the exception of nutrient sources (significant drop from intermediate to weak)<br>3. Children under 10 years significantly improved in practices and handwashing while significantly decreasing their score in dental care		
1. There was a significant improvement in hygiene practices 3 months after intervention<br>2. Regular handwashing increased from 42% to 79%, nail trimming from 36% to 74%, brushing teeth twice daily from 28% to 69%, and regular bathing from 60% to 87% (p<0.001 for all)<br>3. The incidence of diarrheal episodes decreased from 31% to 14%, respiratory infections from 45% to 21%, and skin infections from 18% to 7% (p<0.05)<br>4. Improvement in hygiene scores was significantly associated with reduced morbidity (p=0.003		
1. Improvements in good oral hygiene habits and diet<br>2. Number of sugary drinks consumed daily declined<br>3. According to interview participants, even though students may be aware of recommended oral health practices, they will not necessarily implement them<br>4. Not all dental terminology and scientific knowledge was retained long term because of this initiative; however, improvements were observed when reinforcement of messages was delivered regularly throughout the study duration		
1. Knowledge overall scores of healthy lifestyles increased significantly in 8-year-old students (34.09% improvement) and 11-year-old students (30.21% improvement)<br>2. Factors such as governorate, normal-weight status, and close supervision influenced improvement	9	Shifts in Children's Oral Health Knowledge and Attitudes *(personal and oral hygiene, cavities, importance of fluoride, diet, nutrition, healthy snacking, and healthy lifestyle)*
1. Post-intervention, 99.05% reached high knowledge levels<br>2. Significant knowledge gain observed for both girls and boys (p < 0.0001)		
Highly significant improvement in knowledge of immunization and dental care among children post-intervention		
1. Personal hygiene knowledge increased in treatment groups (pre-test: 8%; post-test: 97%), with no change in control group<br>2. Positive attitudes towards personal hygiene increased in treatment group (pre-test: 61%; post-test: 100%), with slight decrease in control groups (pre-test: 100%; post-test: 92%)		
1. Improvements in knowledge related to oral hygiene, dental health, and diet; and participant's understanding of effect on fluoride on teeth<br>2. Number of sugary drinks consumed daily declined<br>3. According to interview participants, even though students may be aware of recommended oral health practices, they will not necessarily implement them<br>4. Not all dental terminology and scientific knowledge was retained long term because of this initiative; however, improvements were observed when reinforcement of messages was delivered regularly throughout the study duration		
1. Up to 66% of 5th grade students at post-test acknowledged the occurrence of cavities, whereas 25-33% children from all grades were unable to do so at baseline<br>2. Responses of students from 6th and 7th grade were concentrated on bad breath during baseline. After completion of theoretical activities, their responses were more diverse with raised awareness of occurrence of dental cavities<br>3. At post-test, all students were aware that dental problems lead to other diseases		

1. All children were aware of the scheme being run in their school and were aware of the importance of healthy snacking for teeth and overall health<br>2. Children felt that they ate their lunches better and were less interested in sugary and fatty foods		
1. Nutrition attitude was considerably affected - overall score increased from 0.27 to 0.40 (P=0.001)<br>2. Competence in nutrient sources was negatively affected (from 0.64 to 0.48, P=0.001), especially among girls<br>3. Children over 9 years significantly benefited from the course (increased knowledge, attitude, practices, food choices, handwashing), with the exception of nutrient sources (significant drop from intermediate to weak)		
Improved knowledge of health, nutrition, hygiene, and sanitary practices among students		
1. Intervention group had lower scores on Child Perception Questionnaire (CPQ), representing better oral health related quality of life, accounting for 6.07 CPQ points at 2 weeks post-intervention and 6.50 CPQ points at 3-months post intervention<br>2. Intervention group had sense of coherence points 6.39 higher than those of the controls at 2 weeks post-intervention and 4.05 at 3-months post-intervention<br>3. More children in intervention group had normal gingivae (31.81%) than the controls (19.51%) [χ^2 (1) = 4.41, p = 0.04] at 3-months post-intervention	3	Impact on Children's OHRQoL *(including sense of coherence)*
1. Prevalence of overall impacts (on daily performance) was 57.8% among intervention children and 60.8% among control children<br>2. Significantly less intervention children had oral impact on cleaning teeth (p = 0.034) and having 'very severe' level of impact intensity on speaking (p = 0.038)<br>3. Significantly more intervention children reported having 'very little' and 'little' levels of impact intensity on cleaning teeth (p = 0.037) and emotional stability (p = 0.020), respectively<br>4. Significantly lower proportion of intervention children reported bleeding gums (31.4%) and presence of plaque or calculus (7.5%) as causes of oral impacts compared to control group, respectively (35.5%, p = 0.016; 9.7%, p = 0.032)		
1. Children in intervention group (Child Perceptions Questionnaire score mean: 7.22) reported fewer impacts of oral health on their daily lives than those in control group (9.14) at 3-month post intervention<br>2. Sense of coherence significantly improved in intervention group (SOC mean at 2-weeks: 52.98; SOC mean at 3-months: 52.75) compared to control group (SOC mean at 2-weeks: 52.21; SOC mean at 3-months: 51.65)<br>3. Improved sense of coherence predicted better functional and symptom status (oral health related quality of life) at 3-month follow up<br>4. Improved sense of coherence, higher age of child, greater mother's education, lower DMFT scores, and intervention indirectly predicted better functional status at 3-month follow-up via better perceived symptoms<br>5. Higher age of child and the intervention also indirectly predicted better functional status at 3-month follow-up via sense of coherence improvement<br>6. Intervention predicted better-perceived symptom at 3-month follow-up via sense of coherence improvement		
1. Health messages formed part of curriculum (96% schools), but staff were not confident in implementing due to lack of oral health knowledge<br>2. Program created oral health awareness for educators / parents (23% schools) and awareness of correct brushing technique (31% schools)	5	Shifts in Parents and Educators' Oral Health Knowledge and Attitudes
Significant increase in guardians' knowledge about causes of tooth decay and educational value of play, toys, and importance of chatting		
Knowledge and positive attitude of teachers in experimental group changed at 40% and 20%		

respectively compared to control group (5% and 8%)		
1. More than 95% teachers and physical education coaches were enthusiastic about activities<br>2. More than 90% teachers reported being enthusiastic about promoting toothbrushing in schools		
Perceived importance of health issues<br>1. Relatively few principals viewed dental problems (30%) or cigarette smoking (5%) as important health issues among students attending their schools<br>Availability of HPIs (health promotion interventions)<br>2. More than 80% schools offered HPIs related to oral health, bullying, physical activity, and sex education - each of which is government mandated<br>3. Less than half of schools offered HPIs related to injury prevention (45%) or mental health (42%), and only 9% offered tobacco control HPIs<br><br>Alignment between HPI availability and perceived importance of health issue<br>1. HPI availability generally aligned with perceived importance of the health issue; except for oral health HPI, available in 94% of schools, but only 29% of principals in schools with oral health HPIs reported that dental problems were an important issue<br>2. Also, HPI availability and perceived importance were tightly aligned for tobacco control - 91% of principals reported no tobacco control HPIs in their school, and 87% of these principals regarded smoking as an unimportant issue		
1. Proportion of mothers performing weekly cleaning and checking their child's teeth after brushing was significantly higher for experimental group<br>2. Proportion of teachers who gave oral health instruction to children increased at 34% in experimental group compared to 7% in control group<br>3. Teachers in experimental group allocated significantly more time for oral health education	3	Parents and Teachers Behavioural Outcomes *(improvements in engagement in children's oral hygiene and oral health education)*
One of the schools engaged in a nutrition education promotional activity during the Global Handwashing Day, while another school exploited recipes learned during nutrition course to ensure children have a regular intake of proteins		
Parents started sending tiffin from home and started teeth brushing		
1. Schools perceived dental health (p<0.0l) to be improved after scheme introduction (non-parametric T-tests)<br>2. Schools perceived litter reduction (p<0.001), wastage of school meals (p<O.S), alertness and concentration of pupils (p<0.001), behaviour of children (p<0.001) and dental health (p<0.01) to be improved after scheme introduction (non-parametric T-tests)<br>3. Teachers felt that children were more aware of healthy eating messages as a result of the scheme in their schools (58%)<br>4. Teachers also stated that children brought different varieties of fruits and vegetables for break time, the scheme stimulated children to make healthier choices at lunchtime, and that the scheme was being incorporated into the curriculum i.e. science, maths, English<br>5. Teachers and parents alike stated that they felt that if a child had arrived at school without breakfast, this would warrant additional snacks being taken at snack time	1	School and Parents Perceptions of Children's Knowledge, Behaviors and Clinical Outcomes
1. All countries except FSM and Palau, taught hygiene and oral health and all countries except FSM taught mental health and healthy eating<br>2. Hygiene was taught as part of health, physical education, sciences, social studies, home economics, morals, religion, and language<br>3. Mental health was taught as part of health (as part of morals or religion in Japan and Indonesia)	5	School Curriculum and Program Implementation

4. Nutrition and oral health education were taught mainly as part of health, home economics, and social studies (taught as part of health or nutrition in Philippines, Marshall Islands, and Fiji; and as part of morals, home economics, and social service in Japan and FSM)<br>5. Hygiene and oral health were more likely to be taught in primary schools than in high schools		
1. Health messages formed part of curriculum (96% schools), but staff were not confident in implementing due to lack of oral health knowledge<br>2. Daily toothbrushing was conducted by only 8 schools		
One of the schools engaged in a nutrition education promotional activity during the Global Handwashing Day, while another school exploited recipes learned during nutrition course to ensure children have a regular intake of proteins		
1. 77% of toothbrushing was conducted in bathroom after lunch and 23% was conducted in classroom as part of a class<br>2. There was variability in implementation across the two schools, with N = 33 times the toothbrushing activity was conducted in school 1 vs.10 times in school 2 during program period		
Availability of HPIs (health promotion interventions)<br>1. More than 80% schools offered HPIs related to oral health, bullying, physical activity, and sex education - each of which is government mandated<br>2. Less than half of schools offered HPIs related to injury prevention (45%) or mental health (42%), and only 9% offered tobacco control HPIs		
1. Teachers requested the following: (i) suggestions on how to deal with class parties and rewards, (ii) assistance with providing fruits and vegetables in the school at a cost price, (iii) a local contact person whom they could identify as the link person for the healthy breaks scheme, and (iv) resources and support materials for teaching<br>2. A small proportion of parents felt that Scheme criteria were too strict and suggested inclusion of sandwiches<br>3. A small proportion of children cited that they would like one day a week where they could have 'naughty' snacks	4	Program Feedback *(from children, parents, teachers, schools)*
All lessons and activities were implemented and adequate equipment were available		
1. As shown by low rate of refusal, the program and evaluation study were well accepted<br>2. High degree of acceptance of teacher training and appropriateness of the GeKoKidS program were reported<br>3. As per first implementation meeting, participating teachers from intervention schools communicated that GeKoKidS program was predominantly implemented on health days and was integrated within the school mission statement but had not yet been implemented within classes<br>4. During second meeting, teachers reported that GeKoKidS program had been further implemented within classes		
Suggestions from stakeholders<br>1. Efforts to make the program sustainable by integrating in government system<br>2. Active involvement of Ministry of Education<br>3. Coordination between stakeholders to generate resources<br>4. All teachers should be trained on SHN program implementation, with frequent and longer trainings, expanded to other parts of the country		
Barriers and challenges identified for successful implementation of oral health promotion programme included lack of funds, human resources, knowledge and ownership, as well as high workloads and time constraints	3	Program Implementation Challenges

1. Program discontinued once supplies were depleted and resource availability was a challenge to program success (100% schools)<br>2. Learners did not have toothbrushes and paste to brush their teeth at home (92% schools)<br>3. Time constraints limited implementation (70% schools)<br>4. Availability of some support for oral health interventions (39% schools); Support from Colgate World of Care and provincial Department of Health was not continuous and was dependent on availability of supplies, and only 1 school had nurse support<br>5. Water access a major barrier to intervention2 implementation and success (38% schools)<br>6. Students chewed toothbrush / ate toothpaste (23% schools)<br>7. Low staff numbers resulted in higher workloads (8% schools)<br>8. No budget for oral health from provincial Department of Basic Education<br>9. Educators recommended parents to purchase supplies, but this was unaffordable for parents<br>10. Lack of adequate basins, taps, and cups for rinsing and storage<br>11. Learners left the space untidy, leading to time wastage in cleaning<br>12. Hygienic storage of toothbrushes and toothpaste supplies was challenging, especially in larger classes<br>13. Labelling and distribution of toothbrushes was time consuming<br>14. Educators sent toothbrushes and toothpaste home for learners to use; however, some learners did not use them at home or lost them<br>15. Program discontinuation due to inability to secure sponsorships for supplies and large classes		
1. Lack of coordination between stakeholders led to lack of planning for program sustainability and scale-up<br>2. Allocated funds insufficient to implement all program components<br>3. Insufficient human resources and physical infrastructures<br>4. Limited training opportunities especially at district and school level		
Policies included statements on oral health promotion, but was not translated into practice at school level	3	Oral Health Related School Policies (strong healthy eating school programs, favourable neighbourhood environments, policy statements)
1. Cooperation between schools and community improve<br>2. Communities were mobilized in SHN activities, leading to community awareness<br>3. Open defecation decreased and more toilets were built		
Three distinct school environment types were identified: Type 1 and 2 schools had strong healthy eating programs, whereas Type 3 had weak programs. Type 1 schools had favourable neighbourhood food environments, whereas Type 2 and 3 had unfavourable ones.		
