# Supplementary material for: Integration of oral health promotion in primary school health programs: A scoping review
Source: PLOS Glob Public Health. 2026 Oct 5;6(10):e0007390. doi: 10.1371/journal.pgph.0007390 (PMC13638057; doi:10.1371/journal.pgph.0007390)
Supplement: Supplementary file 4 [file pgph.0007390.s004.pdf]

S4 Appendix. Reasons for Exclusion of Studies During Full-Text Screening

Studies excluded (n = 293)

No mention of existing health promotion program (n = 63)

Non-English full text (n = 61)

Wrong study design (n = 28)

Preschool population (n = 19)

No description or mention of oral health intervention (n = 17)

Details Unavailable (n = 14)

Wrong setting (n = 13)

Care or Service-related Program (n = 10)

Curriculum document (n = 9)

Perspective paper (n = 7)

Study Protocol (n = 5)

Adolescent population (n = 5)

Secondary school population (n = 5)

Review paper (n = 4)

Program manual (n = 3)

Not based in elementary school (n = 3)

Details of study population unavailable (n = 3)

Letter (n = 3)

Not Peer-Reviewed (n = 3)

Description of curriculum development process (n = 2)

Description of intervention development process (n = 2)

Guideline document (n = 2)

Government document (n = 1)

Correspondence article (n = 1)

University intervention (n = 1)

Survey of adults on dental health (n = 1)

Description of another included paper (n = 1)

Study exploring views of program coordinators (n = 1)

Question and Answers (n = 1)

Editorial (n = 1)

Commentary (n = 1)

News article (n = 1)

Adult population (n = 1)

Practicum Report (n = 1)
