# Supplementary material for: Integration of oral health promotion in primary school health programs: A scoping review
Source: PLOS Glob Public Health. 2026 Oct 5;6(10):e0007390. doi: 10.1371/journal.pgph.0007390 (PMC13638057; doi:10.1371/journal.pgph.0007390)
Supplement: Supplementary file 5 [file pgph.0007390.s005.pdf]

S5 Appendix. Study Aims, Study Population, and Sample Size

Study Number	Covidence Study ID	Author(s) and Publication Year	Study Aim/Purpose	Study Population	Sample Size of Study Population Including Gender Distribution
**1**	# 2121	Truin et. al. 1981	Assess changes in dental health as a result of the campaign 'Haagse Aktie', based on dietary and dental health education	7-, 9-, and 11-year-old children in the Haque, Netherlands	532 children (7-year-old children = 191 9-year-old children = 179 11-year-old children = 162)
**2**	# 11130	Knight and Grantham-McGregor 1985	Report results of a project carried out in a remote rural Jamaican school, with the aims to improve school children's and their guardians' knowledge and practices related to immunization, dental care, and child development	Grade 3, 5, and 7 children and their mothers or guardians from a remote, rural, primary school in Jamaica	77 children<br>85 guardians
**3**	# 2007	Butcher et al. 1988	Describe need for cardiovascular health education in public schools in Jefferson Parish (county), New Orleans, United States, and identify objectives for the nation which are being addressed by one such intervention	Children from elementary public schools in Jefferson Parish (county), New Orleans, United States	Not applicable
**4**	# 1668	Freeman et al. 2001	Assess effectiveness of community partnership in implementing Boost Better Breaks (BBB) school policy, to promote healthier eating and reduce childhood dental caries	9-year-old children across 16 primary schools from rural part of Northern Ireland	201 children (intervention = 99; control = 102)
**5**	# 17548	O'Neill and O'Donnell 2002	Not mentioned (On reading the full-text, the aim of the study seems to be "to evaluate the Smart Snacks Scheme" for improving snacking habits of school children)	Phase 1 involved health education coordinators of primary schools<br><br>Phase 2 involved one-to-one interviews with teaching staff and a sample of parents<br><br>Phase 3 involved undertaking focus groups with school-children	Not specified
**6**	# 1579	Petersen et al. 2004	Assess oral health outcomes of a school-based oral health education (OHE) programme on children, mothers, and school teachers in China, and evaluate the methods applied and materials used	Children from primary schools, their mothers, and teachers in Wuhan city, central China	At baseline:<br>803 children and their mothers (experimental = 404; control = 399)<br>369 teachers<br><br>At follow-up:<br>666 children and their mothers (experimental = 335; control = 331)<br>347 teachers
**7**	# 1315	Freeman and Oliver 2009	Evaluate effectiveness of Boosting Better Breaks - break-time policy in reducing obvious decay experience and sugar snacking in a cohort of nine-year old children attending intervention and control primary schools	9-year-old children from grade 5 and their parents/guardians from primary schools from rural part of Northern Ireland	147 children (intervention = 74; control = 73)
**8**	# 3416	Franze et al. 2011	Describe contents and evaluation of GeKo^{KidS} program, its acceptance by teachers, and describe methods to increase response of schools, students and parents	9- to 13-year-old children from grade 5 children from 19 schools in Greifswald/East Pomerania, Germany, their parents, and teachers	School dentist examinations = 882 children<br>Extended school dentist examination = 863 children<br>Student survey = 852 children Parent survey = 721 parents

**9**	# 1189	Muirhead and Lawrence 2011	Compare school-level oral health outcomes in schools participating in Ontario's "Healthy Schools" program and non-participating schools in York region, Ontario in 2007-2008 and examine effect of neighbourhood socio-economic factors	Children from kindergarten, elementary, and high schools in York Region, Ontario	243 schools (participating = 106; non-participating = 137)<br><br>Children's sample size not specified
**10**	# 989	Monse et al. 2013	Introduce and discuss methodology of a health educational intervention and briefly present its baseline and 1-year outcomes	6- to 7-year-old children in grade 1 from public elementary schools on the island province of Camiguin, Philippines	Intervention = 168 children; Control group 1 = 173 children; Control group 2 = 133 children
**11**	# 1005	Nammontri, Robinson, and Baker 2013	Test the effect of a school-based intervention to enhance sense of coherence on oral health related quality of life in children	Children from primary schools in Khonkaen, Thailand	12 primary schools (intervention = 6; control = 6)<br><br>2- weeks post-intervention:<br>260 children (intervention = 133; control = 127)<br><br>3- months post-intervention:<br>257 children (intervention = 132; control = 125)
**12**	# 927	Yusof and Jaafar 2013	Compare children's oral health related quality of life (OHRQoL) in schools with 6 years of implementation of a health promoting school model (Doktor Muda - DM) in Malaysia and in schools without the program	Children from elementary schools in Negri Sembilan, Malaysia	3285 children (52.8% girls) (intervention = 1224; control = 2061)
**13**	# 3024	Reddy and Singh 2015	Understand the contextualised delivery of oral health service provision within Health Promoting Schools, to conduct a situational analysis of existing services provided at these schools, and to review current health and education policies	Basic education manager involved with health promotion<br><br>Provincial and district health promotion managers from Department of Health<br><br>School principals across 23 primary health-promoting schools in KwaZulu-Natal, South Africa	23 schools
**14**	# 605	Borges-Yáñez, Castrejón-Pérez, and Camacho 2017	Assess short-term efficacy of supervised tooth brushing for reducing dental plaque, gingival inflammation, and gingival bleeding in schoolchildren participating in SaludARTE program during academic year 2013-2014 and to compare them with children from a school that was not participating in the program	6- to 13- year-old children from public elementary schools in Mexico City and their parents	186 children from 5 schools 12-weeks post-intervention (4 intervention schools = 151 children; 1 control school = 35 children)<br><br>159 parents (89.3% females) (intervention = 129; control = 30)
**15**	# 607	Duijster et al. 2017	Describe findings of a health promotion intervention (FIT) on the parasitological, weight and oral health status of children from attending schools	Children from public elementary schools in Cambodia, Indonesia and Lao PDR	1499 children from 82 schools (intervention = 768 children from 41 schools [girls= 51.6%]; control = 731 children from 41 schools [girls= 46.1%])
**16**	# 570	Edasseri et. al. 2017	Identify distinct school environments based on oral health promoting and neighbourhood environmental factors, and to estimate the relation between school environment types and 2-year dental caries incidence among Québec children aged 8-10 years	8- to 10-year-old White children at risk of obesity and their families from Greater Montreal schools	330 children attending 200 schools
**17**	# 2865	Reddy and Singh 2017	Assess efficiency and sustainability of toothbrushing program implemented at health-promoting schools in KwaZulu-Natal Province, South Africa	Health-promoting primary schools in KwaZulu-Natal, South Africa including the school health teams	Phase 1 (Assessment): 23 schools<br>Phase 2 (Implementation): 20 schools<br>Phase 3 (Review): 13 schools

**18**	# 486	Qadri et. al. 2018	Investigate effect of oral health component of a multidisciplinary health-promoting program on children's dental health in primary schools in West Pomerania, Germany	Children in grade 5 from primary schools in West Pomerania, Germany	18 schools (intervention = 8; control = 10)<br><br>740 children (48% girls) (intervention = 336; control = 404)
**19**	# 18169	Shrestha et al. 2019	To explore the School Health and Nutrition (SHN) program implementation, its impact, and challenges in Nepal	Focal persons for SHN program from central level (Ministries of Health and Education), aid agency, district level (district education office and health office), and from school (focal teacher/school principal), as well as local non- government organization /resource person/School Management Committee (SMC) member	32 key informants across seven districts in Nepal
**20**	# 437	Tomazoni et. al. 2019	Test effectiveness of a school-based intervention to enhance sense of coherence (SOC) and oral health related quality of life (OHRQoL) of socially vulnerable Brazilian children, and explore pathways by which the intervention may improve OHRQoL and SOC	8- to 14-year-old children (in grades 4 and 5) in Santa Maria, Brazil	2-weeks post intervention:<br>356 children from 5 primary schools (intervention = 165 children from 3 schools; control = 191 children from 2 schools)<br><br>3-month follow-up:<br>342 children (intervention = 158; control; = 184)
**21**	# 301	Duijster et. al. 2020	Explore whether a school-based water, sanitation and hygiene programme, which includes group hygiene activities, contributes to the formation of independent handwashing and toothbrushing habits among Filipino children.	Children studying in grades 2 to 6 from 20 public elementary schools in the Batangas province of Philippines	For handwashing:<br>20 schools at endline (intervention = 10; control = 10)<br><br>For toothbrushing:<br>503 children (from grades 2 to 6) at endline from 10 schools (intervention = 5; control = 5)<br><br>For toilet satisfaction and child surveys:<br>320 children (from grade 4 only) at endline from 20 schools (intervention = 10; control = 10)
**22**	# 14348	Riya, Arief, and Kurnia 2020	Explain the effect of health education methods review horay courses and videos media on improving personal hygiene abilities	Children from elementary schools in Surabaya, Indonesia	173 elementary school students from 2 elementary schools (intervention = 36; control = 36)
**23**	# 2298	Loch et. al. 2021	Evaluate whether intervention improved oral health knowledge and oral hygiene practices among participants, and investigate whether those were retained 18-months after program cessation	5- to 12- year-old children from one primary school in Dunedin, New Zealand that adopted a Māori immersion	20 children (65% girls) for questionnaire (5 to 6 years = 10 children; 7 to 9 years = 6 children; 10 to 12 years = 4 children)<br><br>6 children (50% girls) participated in semi-structured interviews 18-months post-intervention

**24**	# 8538	Petrus-Vancea and Stirb 2021	Identify oral problems and promote a healthy lifestyle at gimnazial school pupils from a Romanian disadvantaged area, and identify progress made by pupils following theoretical or practical interventions	11- to 14- year-old school children from a rural Romanian disadvantaged area	44 children
**25**	# 17165	Riglea et. al. 2022	Describe social inequalities across elementary schools in: (i) level of importance that school principals attribute to 13 common health-related issues among students in their school; (ii) availability of health promotion interventions (HPIs) within their school addressing 8 health topics and (iii) (mis)alignment between perceived importance and HPI availability	School principals and/or nominated staff members from public elementary schools in Quebec	171 public elementary schools from 32 school boards in Quebec (females = 60%; principals = 93%' vice-principals = 4%; teachers = 3%; average participant age = 47.3 years)
**26**	# 9281	Sharma. et. al. 2022	Present the intervention framework and results of feasibility testing of CATCH (Coordinated Approach to Child Health) Healthy Smiles oral health promotion program among children in grades K-2 in Houston, Texas (pilot study)	Children from kindergarten and elementary schools and their parents in Houston, Texas, USA	125 parent-child dyads from 2 schools (kindergarten = 31; grade 1 = 42; grade 2 = 52) Children with equal gender distribution<br><br>Mean parent age = 35.3 years (90% parents were women)
**27**	# 18173	Szczepanska et al. 2022	Assess change in knowledge of healthy eating principles among 7–9-year-old children following participation in the "Colourful means healthy" nutrition education project	Children from grade 2	317 children (47% females)
**28**	# 4723	Yamgai et. al. 2022	Combine nutrition, handwashing, and dental care education to improve health literacy of school children and boost engagement of parents and teachers in promotion of nutrition-related behaviours conducive to health and well-being	Children from primary schools in in Yaoundé, Cameroon	71 children from 4 primary schools (50.7% girls)
**29**	# 21	Nishio et. al. 2023	Identify components of health-related topics and in which subjects they were taught in primary, secondary, and high school curricula of 8 countries	School health specialists from Japanese school health consortium, representatives and curricula from each of the 8 partner countries	8 countries<br>5 school health specialists
**30**	# 9111	Moussi et al. 2024	Assess impact of an awareness campaign on health knowledge and practices of 5-, 8-, and 11-year-old students throughout pre- and post-intervention testing focused on general health and healthy habits	5-, 8-, and 11-year-old children from 27 public and semi-private schools in Lebanon and their parents	1144 children (52.4% females) 5 years old = 448; 8 years old = 350; 11 years old = 346)
**31**	# 21026	Shaikh, Prasidda, and Eruva 2025	Assess the impact of a structured health education intervention on personal hygiene practices and morbidity patterns among primary school children through a collaborative pediatric and Preventive and Social Medicine (PSM) approach	6- to 12-year-old children in grades 1 to 5 from two government primary schools	200 children from two schools (54% boys; 46% girls) (mean age = 9.1 ± 1.8 years)
