# Supplementary material for: Integration of oral health promotion in primary school health programs: A scoping review
Source: PLOS Glob Public Health. 2026 Oct 5;6(10):e0007390. doi: 10.1371/journal.pgph.0007390 (PMC13638057; doi:10.1371/journal.pgph.0007390)
Supplement: Supplementary file 6 [file pgph.0007390.s006.pdf]

S6 Appendix. Content and Activities of General Health Programs and Activities of Oral Health Programs

Study Number	Covidence Study ID	Author(s) and Publication Year	General Health Content	General Health Activities	Oral Health Activities
**1**	# 2121	Truin et. al. 1981	Nutrition	1. Haque city council spent 200,000 guilders per year for a long-term dental health campaign<br>2. Committee consisting of school health and dental services representatives was established to form a multidisciplinary working group consisting of a physician, a dentist, a dietician and some educators<br>3. Educational material for use by school teachers, health service workers<br>4. Group developed educational material for use by school teachers, parents, health service workers<br>5. Since 1972 the 6-9-year-old children and since 1974 the 9-11-year-old children in elementary schools were reached by information, wall posters, and learning materials	1. Haque city council spent 200,000 guilders per year for a long-term dental health campaign<br>2. Committee consisting of school health and dental services representatives was established to form a multidisciplinary working group consisting of a physician, a dentist, a dietician and some educators<br>3. Educational material for use by school teachers, health service workers<br>4. Group developed educational material for use by school teachers, parents, health service workers<br>5. Since 1972 the 6-9-year-old children and since 1974 the 9-11-year-old children in elementary schools were reached by information, wall posters, and learning materials
**2**	# 11130	Knight and Grantham-McGregor 1985	1. Child health and development (developmental milestones and child management techniques)<br>2. Immunization (purpose, diseases which immunization can prevent, when baby should be immunized)	1. Learning activities involving role play, group discussions, demonstrations, drama, poster competitions, toy making from waste household materials<br>2. End-of-year open day<br>3. Class teachers assisted by principal researcher to implement an action-oriented child friendly curriculum (1 hour of learning each week of the school year i.e. 30 sessions over 3 terms)	1. Learning activities (role play, group discussions, demonstrations, drama, poster competitions, toy making from waste household materials)<br>2. End-of-year open day<br>3. Class teachers assisted by principal researcher to implement an action-oriented child friendly curriculum (1 hour of learning each week of the school year i.e. 30 sessions over 3 terms)
**3**	# 2007	Butcher et al. 1988	1. Cardiovascular health<br>2. Anatomy and physiology<br>3. Knowledge of hypertension<br>4. Behaviour skills<br>5. Stress management<br>6. Smoking prevention<br>7. Exercise behaviour<br>8. Nutrition and diet<br>Child abuse prevention	1. Teacher training<br>2. Use of teacher's guides<br>3. Classroom instruction (modules)<br>4. Use of student activity workbooks<br>5. Physical education program / aerobic fitness classes<br>6. Healthy school lunches<br>7. Parent education (family health) through newsletter delivering information and recipes to families and siblings of Heart Smart students<br>8. Behavioural pledges<br>9. Parents participation facilitated through COR (Heart) Committee composed of parents and teachers<br>10. Health fairs<br>11. Volunteer-assisted periodic risk factor screenings for staff and students<br>12. Periodic assessments of body composition	1. Teacher training<br>2. Use of teacher's guides<br>3. Classroom instruction (modules)<br>4. Use of student activity workbooks<br>5. Healthy school lunches<br>6. Parent education (family health) through newsletter delivering information and recipes to families and siblings of Heart Smart students<br>7. Behavioural pledges<br>8. Parents participation facilitated through COR (Heart) Committee composed of parents and teachers<br>9. Health fairs<br>10. Classroom activities to enable children to recognize and choose healthy foods (measuring portions of food, reading food labels, identify and compare nutrient content of common foods)<br>11. Food service workers in cafeteria learn principles and

				13. Classroom activities to enable children to recognize and choose healthy foods (measuring portions of food, reading food labels, identify and compare nutrient content of common foods)<br>14. Food service workers in cafeteria learn principles and procedures for reducing salt, sugar, and fat in recipes<br>15. Students keeping a log book to monitor fitness level and exercise plans<br>16. Fitness tests<br>17. Development of peer support systems; Role-plays	procedures for reducing salt, sugar, and fat in recipes
**4**	# 1668	Freeman et al. 2001	Healthy diet	1. Implementation of school policy allowing only consumption of milk and fruit at break-time<br>2. Policy development in partnership among researchers, practitioners, and school community (dieticians, school meal advisors, teachers, health promotion officers, and local dairy suppliers of school milk)<br>3. Co-development of policy with parents, teachers, and governors of the schools and pre-school groups within school environment<br>4. Awareness building and negotiation<br>5. Written policy, approved by the Board of Governors<br>6. Schools must agree not to sell snacks high in fat or sugar in school<br>7. Teachers must agree not to reward students with candy<br>8. Facilitate healthier eating by empowering children to make healthier choices<br>9. Monitoring of school activity by Community practitioners and Community Dental Service for difficulties, policy compliance and adherence	1. Implementation of school policy allowing only milk and fruit consumption at break-time<br>2. School break-time policy (Boosting Better Breaks - BBB), developed in partnership among researchers, practitioners, and school community (team of dieticians, school meal advisors, teachers, health promotion officers, and local dairy suppliers of school milk)<br>3. Awareness building, negotiation, and co-development of policy with parents, teachers, and school governors and pre-school groups within school environment<br>4. Schools must agree not to sell snacks high in fat or sugar in school environment<br>5. Teachers must agree not to reward students with candy<br>6. Facilitate healthier eating by empowering children to make healthier choices<br>7. Community practitioners and Community Dental Service monitor school activity, keeping an eye out for difficulties encountered and policy compliance and adherence
**5**	# 17548	O'Neill and O'Donnell 2002	Healthy foods	Smart snacks scheme (which runs annually) consisted of following criteria finalized by health promotion staff in conjunction with dietetic, dental, environmental health and education staff:<br>1. During mid-morning breaktime, only milk/water and/or fruit/vegetables should be allowed<br>2. Written healthy snacks policy<br>3. All parents must be informed in writing of the school's intention to adopt a healthy snacks policy and the benefits to the school	1. Only milk, water, fruits, and vegetables allowed during breaktime<br>2. Written healthy snacks policy<br>3. Written information sent to parents about school snack policy
**6**	# 1579	Petersen et al. 2004	General health, diet, sugar, and health	Classroom instructions by trained teachers	1. Classroom instructions by trained teachers<br>2. Children's participation in daily oral hygiene instructions in presence of mothers and supervised by teachers<br>3. Mothers informed of dental care of children

**7**	# 1315	Freeman and Oliver 2009	Healthy diet	1. Implementation of school policy allowing only consumption of milk, water, vegetables, and fresh fruit at school break-times<br>2. Closing of tuck shops<br>3. Assurance from teachers that confectionery, cakes, biscuits or sugar-sweetened soft drinks will not be given as rewards or prizes<br>4. Negotiated with all stakeholders to ensure that principal teachers, class teachers, parents, wholesalers and providers of milk, fruit and vegetables developed partnerships to support the break-time policy	1. Introduction of healthy foods at school break-times (milk, water, vegetables, and fresh fruit)<br>2. Closing of tuck shops<br>3. Assurance from teachers that confectionery, cakes, biscuits or sugar-sweetened soft drinks will not be given as rewards or prizes<br>4. Negotiated with all stakeholders to ensure that principal teachers, class teachers, parents, wholesalers and providers of milk, fruit and vegetables developed partnerships to support the break-time policy
**8**	# 3416	Franze et al. 2011	1. Health literacy<br>2. Nutrition<br>3. Physical activity<br>4. Vaccination<br>5. Smoking prevention<br>6. Chronic pain prevention<br>7. Obesity prevention<br>8. Improving nutritional status<br>9. Important nutrients and their effects on body and related foods<br>10. Relation between nutrient and energy supply<br>11. Classification of regularly consumed food into different food groups ("food pyramid" and recommendations of healthy food)	1. Health education in regular lectures in subjects biology, physical education and math, and in framework of special project days<br>2. Teachers trained to implement GeKo^{KidS} program and received a booklet containing presentation slides and teaching materials<br>3. Worksheets on dental health and vaccination<br>4. Competitions on vaccination<br>5. Quiz on pain<br>6. Learning stations (to taste and smell different fruits and vegetables, and determine sugar and fat contents of sweets, snacks and beverages)<br>7. Learning about food shopping and how to prepare healthy breakfast<br>8. Invitation of other classmates to a breakfast<br>9. Active games<br>10. "Fitness Pyramid" (to reflect student's physical activity and show ways to increase physical activity)<br>11. "Fitness Olympics" (application of a test for assessing children's and adolescents' motor skills)<br>12. Other activities (cartoons and pictorial illustrations, role-plays, events)	1. Oral health education in regular lectures in the subjects biology and math<br>2. Students use a nutrition schedule to fill beverage and food impulses and schedule analysed by teacher and/or dentist<br>3. Students motivated to participate in biannual, free of charge dentist screening<br>4. Work sheets for calculations of caries prevalence
**9**	# 1189	Muirhead and Lawrence 2011	1. Healthy eating<br>2. Physical activity<br>3. Bullying prevention<br>4. Personal safety and injury prevention<br>5. Substance use and abuse<br>6. Healthy growth and development<br>7. Mental health activities	Not specified	Not specified

**10**	# 989	Monse et al. 2013	8. Personal hygiene	Intervention group:<br>1. Daily supervised handwashing with soap and clean water<br>2. Biannual deworming with single dose of albendazole (400 mg)<br>3. Implemented by teachers (daily tasks) and school health nurses (orientation and supervision)<br><br>External concurrent control group:<br>1. Annual physical examination<br>2. Biannual deworming by school nurses<br>3. Distribution of single (10-ml) commercial toothpaste sachet, a toothbrush, and an oral health message at beginning of school year<br>4. Health education as part of regular school curriculum	For two of the three experimental groups:<br>1. Supervised toothbrushing with fluoride toothpaste (0.3 ml; 1,450 ppm)<br>2. Other caries control interventions (tooth extraction, abscess drainage, medications<br>3. Brushing with fluoride gel
**11**	# 1005	Nammontri., Robinson, and Baker 2013	1. Healthy food choices<br>2. Health awareness<br>3. Health as a continuum<br>4. Problem prevention, symptom reduction, improving and maintaining good health<br>5. Positive thinking about health, self, and others<br>6. Develop positive attitudes, personal skills, and coping strategies<br>7. Body image, self-care, self-efficacy, self-esteem, and self-confidence<br>8. Satisfaction rating of aspects related to family, friends, and schools<br>9. Acceptance of heart or oral diseases<br>10. Diminish anxiety or less effective social responses<br>11. Behaviour rehearsal (practising healthy behaviours)<br>12. Goal setting to improve life, changing life, and control over life	1. Weekly teacher-delivered sessions (co-developed with children)<br>2. Teachers trained by researcher and provided session handbook<br>3. Researcher support to deliver activities<br>4. Classroom activities, didactic teaching and discussion, mask making, role play, games, poster making, in-class presentations<br>5. Teacher-supported healthy school projects (brainstorming, planning, implementation, and evaluation) such as suggesting to others to choose healthy foods; working to run a healthy snack shop<br>6. Children determined that confectionery would not be sold in school<br>7. Participation of whole school, students and staff<br>8. Incorporation of incentives and supportive leadership	1. Educational sessions by trained teachers<br>2. Group activities and projects<br>3. Teaching effective toothbrushing to peers<br>4. Tooth brushing under supervision of teachers and researcher<br>5. Working with teachers to run healthy snack shop<br>6. Children determined that confectionery would not be sold in schools<br>7. Provision of toothbrushes and fluoridated toothpastes by schools to children
**12**	# 927	Yusof and Jaafar 2013	1. Environmental hygiene<br>2. Mental health<br>3. Infectious disease and endemic disease prevention<br>4. Safety and injury prevention	1. Group of children 'Doktor Muda' (DM) selected and trained by trained teachers through modular curriculum, for peer education and empowerment, and to act as role models<br>2. National 'Doktor Muda Program' (DMP) convention held every 2 years to allow idea exchange and promote best practices	1. Weekly toothbrushing with fluoride toothpaste (under supervision)<br>2. Anti-smoking campaign

			5. Healthy nutrition and diet<br>6. Healthy lifestyles and teenagers<br>7. Anti-smoking and drug abuse control<br>8. First aid	3. DM conducted health talks<br>4. Leaflet distribution<br>5. Prepare health messages<br>6. Put up posters<br>7. Organise health quiz, drawing, essay writing, competitions<br>8. Campaigns<br>9. Supervise hand washing and toothbrushing<br>10. Monitor peers<br>11. Keep record of health problems<br>12. Provide first aid<br>13. Help teachers to record student's health report card for weight, height, eye test, health inspection and personal hygiene on school health day<br>14. Conduct DM Club activities on co-curricular days<br>15. Engage in outside school activities, and undertake health campaigns at community elderly centre	
**13**	# 3024	Reddy and Singh 2015	1. Basic hygiene<br>2. Nutrition and food safety<br>3. Tobacco use<br>4. HIV/AIDS<br>5. Sexual abuse<br>6. Agriculture<br>7. Recycling	1. Health messages formed part of the curriculum<br>2. Promotion of healthy food in schools<br>3. Annual provision of school health services and screenings by Department of Health<br>4. Community involvement in school health (activities include HIV/AIDS, sexual abuse, agriculture, recycling, tobacco use, nutrition and food, safety)	Education, tooth brushing, fluoride mouthrinse, fissure sealant (inconsistent; not regular)
**14**	# 605	Borges-Yáñez, Castrejón-Pérez, and Camacho 2017	1. Healthy eating (nutrition)<br>2. Hygiene	1. Children receive healthy meals, nutritional counselling to prevent obesity, and health education<br>2. Perform general and oral hygiene practices (supervised tooth brushing)<br>3. Perform physical and cultural activities from Monday to Friday for three hours daily<br>4. Workshops on food, nutrition, and hygiene habits	<u>Children from intervention group:</u><br>1. Taught tooth brushing technique by trained supervisor and teachers<br>2. Performed daily supervised toothbrushing (10-minutes) after meals using fluoridated toothpaste and toothbrush<br>3. Received oral health education offered sporadically during workshops on food, nutrition, and hygiene habits<br>4. Surveillance visits by a supervisor<br>5. Parents or guardians received a written oral health diagnosis of their children<br><br><u>Children from control group:</u><br>1. Received only one lecture about oral health education<br>2. Note describing identified dental issue sent to person responsible for child
**15**	# 607	Duijster et al. 2017	1. Hand washing<br>Deworming (hygiene)	<u>Intervention group:</u><br>1. Daily group handwashing with soap<br>2. Biannual deworming as part of respective national government-coordinated deworming programme<br>3. Construction and maintenance of group washing facilities	<u>Intervention schools:</u><br>1. Daily group toothbrushing with 0.3 ml of toothpaste (containing 1450 ppm free available fluoride)<br>2. Construction and maintenance of group washing facilities<br>3. Educational staff received practical guidance and training support

				4. Educational staff received practical guidance and training materials, but no further support to implement programme activities<br><br>Control group:<br>Implemented regular government health education curriculum and biannual deworming as part of national deworming program	
**16**	# 570	Edasseri et. al. 2017	Healthy eating (nutrition)	1. Favourable school environment i.e. no convenience or fast-food store within 500 m of the school<br>2. Formal school initiatives to promote healthy eating (within management, teachers, community involvement, teacher and parents' education, family engagement)<br>3. Providing healthy food and snacks<br>4. Menu by nutritionist	1. Dental hygiene education<br>2. Dental health professional visits
**17**	# 2865	Reddy and Singh 2017	1. Unhealthy diet/nutrition/healthy eating<br>2. Food safety<br>3. Cleanliness<br>4. Smoking and alcohol<br>5. HIV / AIDS<br>6. Sexual abuse<br>7. Teenage pregnancy<br>8. Trauma and violence<br>9. Agriculture<br>10. Recycling<br>11. Community empowerment	Based on related reference #21 from this paper:<br>1. Health messages in curriculum and parent meetings<br>2. School health services and annual screenings by Department of Health<br>3. Promotion of healthy food through healthier lunches and sale of healthy foods in some schools<br>4. Availability of school health nurses (but they lacked expertise and knowledge in oral health promotion, had large areas to support, and had high workloads)<br>5. Voluntary community involvement in school health programs Appointments were made telephonically with school health teams to formulate and implement interventions<br><br>Based on response from author about details of health promotion activities implemented in schools:<br>1. Health promotion, education, awareness, and basic health messages, visits by school health nurses to talk to students<br>2. Health activities varied depending on learners needs, surrounding community, and available resources	1. Toothbrushing program (daily in 8 schools and 2-3 times per week in 4 schools) (toothbrushes, 3-month supply of toothpaste, and brushing instructions provided - program discontinued once supplies depleted)<br>2. Fluoride mouthrinse and fissure sealant programs (inconsistent frequency)<br>3. Outsourcing of supplies<br>4. Some schools had intervention support from Colgate World of Care and provincial and Department of Health (not continuous and dependent on availability of supplies)<br>5. School visits by oral health personnel when supplies were available<br>6. Colgate World of Care visits once a year<br>7. Weekly visits by nurses in one school to advise children<br>8. Incorporation of oral health education into school curriculum and parent meetings<br>9. Mobile messenger application set up between researcher and school health teams for ease of communication and sharing ideas between schools<br>10. Promotion of healthier lunches<br>11. Sugary snacks control by vendors and tuck shops<br>12. Those that had knowledge of oral health would incorporate this into their teaching.<br>13. Awareness for learners, educators, and parents
**18**	# 486	Qadri et. al. 2018	1. Healthy nutrition<br>2. Health literacy<br>3. Dealing with pain<br>4. Healthy recreation (activities and leisure time)	Intervention schools:<br>1. General health promotion program<br>2. School curricula<br>3. Activities delivered by schoolteachers	For both intervention and control schools:<br>1. Teacher education<br>2. Free check-ups and visits by national health system<br>3. Reduction in consumption of sugar-sweetened beverages

			5. Vaccination<br>6. Smoking	<u>Control schools:</u><br>Teachers and children did not receive additional program or training on health promotion during study period	<u>Additional activities for intervention schools:</u><br>1. Feedback to teachers about caries status, vaccination rates, weight distribution, smoking habits and school meals<br>2. Children and parents informed about examination results<br>3. Prizes for classes with best caries status
**19**	# 18169	Shrestha at al. 2019	1. General health<br>2. First aid<br>3. Deworming<br>4. Iron supplementation<br>5. Nutrition<br>6. Hygiene	1. General health check-ups<br>2. First aid services<br>3. Deworming<br>4. Iron supplementation<br>5. Child club activities<br>6. Special health classes<br>7. Maintaining School Health and Nutrition (SHN) register<br>8. Providing mid-day meals<br>9. School cleaning<br>10. Improving access to safe drinking water, toilet, and hand-washing facilities<br>11. Constructing toilets and waste disposal pits in schools<br>12. Teachers in project schools were trained to conduct SHN activities	Oral health check-up
**20**	# 437	Tomazoni et. al. 2019	1. Thinking positively about health, self, and others<br>2. Healthy food choices<br>3. Body image, self-esteem, self-confidence, self-efficacy, self-care, goal-setting, changing life, control over life, improving personal skills<br>4. Diminish anxiety or less effective social responses, develop positive attitudes and coping strategies<br>5. Behaviour rehearsal (practising healthy behaviours)<br>6. Preventing problems, reducing symptoms, improving and maintaining good health<br>7. Satisfaction rating of aspects related to family, friends, and schools<br>8. Health as a continuum (not just sick/well) and examples	1. Teacher-delivered sessions (1 to 2 hours) developed with children<br>2. Teachers trained by researcher, provided handbook, with researcher support to deliver activities<br>3. Classroom activities involving didactic teaching and discussion; games; mask making; role play; poster making; and in-class presentations<br>4. Healthy school projects through brainstorming, planning, implementation, and evaluation with teacher support - suggesting to others to choose healthy foods; teaching peers how to brush their teeth effectively; working with teachers to run a healthy snack shop<br>5. Children determined that confectionery would not be sold in schools<br>6. Toothbrushes and fluoridated toothpastes provided by schools to children<br>7. Projects involved whole school and all students and staff participated<br>8. Incentives and supportive leadership	1. Educational sessions by teachers, group activities, and healthy school projects (suggesting to others to choose healthy foods, teaching peers how to brush their teeth effectively, working with teachers to run a healthy snack shop<br>2. Children determined that confectionery would not be sold in schools)<br>3. Toothbrushes and fluoridated toothpastes provided by schools to children

			and being happy despite having diseases		
**21**	# 301	Duijster et. al. 2020	Handwashing (personal hygiene)	Intervention schools:<br>1. Children performed daily group handwashing with soap under teacher or student supervision<br>2. One basic ready-to-install group handwashing facility (HWF) per classroom<br>3. Technical support and supplies such as soap, cleaning rotas, toilet user kits, cleaning products, and a manual provided<br>4. A video introducing group hygiene activities<br>5. Stickers for display near toilet and hand-washing facilities<br><br>Control schools:<br>Informed about Water, Sanitation and Hygiene (WASH) in School policy (promotion of daily group hygiene activities, with no hardware or consumables provided)	Intervention schools:<br>1. Daily supervised toothbrushing with fluoride toothpaste by children under teacher or student supervision<br>2. Toothpaste and technical support provided<br>3. Video showing group hygiene activities<br>4. Stickers for display near toilet and hand- washing facilities<br><br>Control schools:<br>Informed about WASH in School policy (i.e. promotion of daily group hygiene activities with no hardware or consumables provided)
**22**	# 14348	Riya, Arief, and Kurnia 2020	Handwashing (personal hygiene)	Treatment group interventions:<br>1. Health education (cooperative learning model)<br>2. Showing hand washing and toothbrushing video for ± 15 minutes<br>3. "Course review horay method" (testing understanding of presented material, through direct questions or demonstrations)<br>4. Group formation<br>5. Children explained about handwashing and toothbrushing through power point presentations with opportunity to ask about related material<br>6. Practice of washing hands and brushing teeth observed by facilitators<br><br>Control group interventions:<br>1. Simple video / slide on the general concept of personal hygiene<br>2. After the post-test, control group was given same intervention as treatment group, starting with video screenings	Treatment group interventions:<br>1. Health education (cooperative learning model)<br>2. Showing hand washing and toothbrushing video for ± 15 minutes<br>3. "Course review horay method" (testing understanding of presented material, through direct questions or demonstrations)<br>4. Group formation<br>5. Children explained about handwashing and toothbrushing through power point presentations with opportunity to ask about related material<br>6. Practice of washing hands and brushing teeth observed by facilitators<br><br>Control group interventions:<br>1. Simple video / slide on the general concept of personal hygiene<br>2. After the post-test, control group was given same intervention as treatment group, starting with video screenings
**23**	# 2298	Loch et. al. 2021	1. Combined science outreach<br>2. Types of traditional foods eaten by the Māori<br>3. Importance of physical activities	Unclear	1. Supervised daily toothbrushing<br>2. Oral health education<br>3. Competitions<br>4. Team activities<br>5. Creating healthy breakfast<br>6. Measuring sugar in various foods<br>7. Preparing poster<br>8. Hands-on sessions<br>9. Demonstrations / science experiments

					10. Six interactive activities conducted during school hours in English<br>11. Supervision by trained and calibrated students and staff from dentistry, oral health and human nutrition<br>12. School principal, classroom teachers, and support staff involvement in translating concepts from English to Māori and vice-versa, and ensuring activities delivered with school's cultural values<br>13. Older students supporting younger students
**24**	# 8538	Petrus-Vancea and Stirb 2021	1. Personal hygiene<br>2. Food health<br>3. Influence of nutrition on oral health<br>4. Human anatomy and physiology	<u>For 5th and 6th grade:</u><br>1. Optional subject called 'Education for Health' (EH) added to curriculum<br>2. Supplementary theoretical activities on hygiene/maintaining oral health and influence of nutrition on oral health - 50 minutes each were introduced in chapter 'Personal Hygiene' and 'Food Health'<br><br><u>For 7th grade:</u><br>1. 'Human Anatomy and Physiology' was added to Biology curriculum<br>2. School management team was requested for a scientific counsellor<br>3. Parents were informed about activities	<u>For 5th and 6th grade:</u><br>1. Optional subject called 'Education for Health' (EH) added to curriculum<br>2. 2 supplementary theoretical activities on hygiene/maintaining oral health and influence of nutrition on oral health - 50 minutes each were introduced in chapter 'Personal Hygiene' and 'Food Health'<br>3. Practical intervention added for 5th grade (visit to a dental office volunteered by their biology teacher and the dentist - professional evaluation of oral health and professional brushing)<br><br><u>For 7th grade:</u><br>1. 'Human Anatomy and Physiology' was added to Biology curriculum, consisting of 2 supplementary theoretical information (each of 50 minutes) concerning oral cavity hygiene<br>2. School management team was requested for a scientific counsellor<br>3. Parents were informed about activities
**25**	# 17165	Riglea et. al. 2022	1. Bullying and exclusion<br>2. Physical activity/active living<br>3. Sex education<br>4. Healthy eating<br>5. Personal safety and injury prevention<br>6. Mental health and well-being<br>7. Tobacco control<br>8. Online privacy	1. Teacher led in-class conversations on bullying, followed by a whole-school assembly<br>2. Teacher-led workshops and storytelling<br>3. Teacher-hosted cooking workshops with rare and lesser-known healthy ingredients<br>4. Students take turns pedalling on a stationary bicycle for 20 minutes during normal class activities (kilometres compiled and accumulated for entire school)<br>5. Workshops offered by community organization (on online privacy, legal and criminal aspects of non-consensual distribution of intimate images)<br>6. School visits by community-based organization to talk to children about how to safely travel from home to school on their bicycle<br>7. Nurse and social worker partner to provide workshops on health and environmental impacts of tobacco<br>8. Trip to the shore to pick up cigarette butts	1. Dental hygienist visits for school-wide dental screening<br>2. In-class presentations to students on proper teeth brushing technique<br>3. Nurse and social worker partner to provide workshops on health and environmental impacts of tobacco<br>4. Trip to the shore to pick up cigarette butts

**26**	# 9281	Sharma et. al. 2022	1. Unhealthy diet and nutrition (increased consumption of fruits, vegetables, water and unflavoured milk; decreased consumption of unhealthy foods such as sugary, starchy, and sticky foods, and sugary drinks)<br>2. Building self-efficacy (for engaging in healthy eating, drinking, mealtime, and snacking behaviours) and focus on subjective norms (healthy eating being a norm for other families with young children)<br>3. Physical activity<br>4. Dental trauma prevention	1. Several structured physical activities (integrating messages on nutrition, oral health education, and dental trauma prevention)<br>2. Social support for parents, teachers, and children (to serve and consume fruits, vegetables, water, and unflavoured milk)<br>3. Bilingual and culturally appropriate parent engagement materials (program website resources, interactive parent-child ‘‘homework activities,’’ infographics, CATCH family fun nights in schools)<br>4. CATCH Healthy Smiles coordination kit (resources and instructions to school administration to integrate and coordinate program activities for simultaneous implementation across the school)<br>5. Digital CATCH online platform (to access program resources for all schools - http://catchinfo.org/)<br>6. Use of train-the-trainer model (to train schools and their staff in program implementation)<br>7. Direct observation of program sessions by trained project staff	1. Regular toothbrushing in schools<br>2. Classroom curriculum for oral health education<br>3. Several structured physical activities (integrating messages on nutrition, oral health education, and dental trauma prevention)<br>4. Social support for parents, teachers, and children (to serve and consume fruits, vegetables, water, and unflavoured milk)<br>5. Bilingual and culturally appropriate parent engagement materials (program website resources, interactive parent-child ‘‘homework activities,’’ infographics, CATCH family fun nights in schools)<br>6. CATCH Healthy Smiles coordination kit (resources and instructions to school administration to integrate and coordinate program activities for simultaneously implementation across the school)<br>7. Digital CATCH online platform (to access program resources for all schools - http://catchinfo.org/)<br>8. Use of train-the-trainer model (to train schools and their staff in program implementation)<br>9. Direct observation of program sessions by trained project staff
**27**	# 18173	Szczepanska et al. 2022	1. Healthy nutrition<br>2. Cooking skills<br>3. Physical activity recommendations<br>4. Hygiene<br>5. Safe work<br>6. Sleep	4 modules consisting of educational, sensory, cooking, and molecular gastronomy workshops, hands-on experiments, educational games, implementation through research and teaching staff, teaching activities by teachers, group activities, story, description, demonstration, observation, brainstorming, simulation	Unclear
**28**	# 4723	Yamgai et. al. 2022	1. Healthy nutrition<br>2. Handwashing (personal hygiene)<br>3. Water, Sanitation, and Hygiene (WASH)<br>4. Safety	1. Modules delivered by staff from non-government organization (NGO)<br>2. At-home activities, teacher-led in-school discussions, drawing, interactive games, puzzles, educational materials, events<br>3. Advocacy with administration, involvement of ministries, school system engagement through audits, donation of resources, use of posters<br>4. Water, Sanitation, and Hygiene (WASH) equipment and disposable material<br>5. PTA meetings and engagement of caregivers/parents for food preferences, education, weekly menu, giving printed recipes, cooking food with local ingredients, package and prepare meals<br>6. Daily monitoring of handwashing and oral hygiene by teachers, observation of daily meals packed by caregivers for children<br>7. Canteen operators’ participation	1. Module on dental care consisting of at-home activities and in-school discussions including drawing, interactive games, puzzle<br>2. Module implemented during lunch and repeated twice in each school by NGO staff<br>3. Use of educational materials (clean water, toothbrush, paste, dental floss, game puzzles, apples, samples of local crops)<br>4. Teacher-led activities, included daily monitoring of handwashing and oral hygiene by teachers

				8. Demonstrations of WASH-related daily good practices with school communities, nutrition-related good practices to teachers, caregivers/parents, canteen operators<br>9. Organisation of cost-effective school feeding program<br>10. Cooking traditional local recipes	
**29**	# 21	Nishio et. al. 2023	1. Personal hygiene (handwashing with soap and physical cleanliness<br>2. Menstrual hygiene<br>3. Environmental hygiene, climate change and global warming, emergency drills and trainings, recycling, resource preservation<br>4. Food hygiene; food safety, security, and supply; basic cooking skills<br>5. Injury prevention and first-aid treatment<br>6. Concept of health and causes of disease<br>7. Infectious diseases<br>8. Mental health (illness understanding; communication skills, coping and seeking support; self-awareness; emotion, stress, and conflict management; stigma towards mental illness)<br>9. Nutrition and healthy eating<br>10. Substance abuse	Inclusion of health-related topics in school curriculum	Inclusion of oral health-related topics in school curriculum
**30**	# 9111	Moussi et al. 2024	1. Sleep schedule<br>2. Screen time<br>3. Personal hygiene<br>4. Physical activity<br>5. Nutrition<br>6. Security<br>7. Bullying<br>8. Addiction	1. Nurse-delivered awareness campaign of 40-45 minutes<br>2. Lectures, power point presentations, and live demonstrations (whiteboard, drawings)<br>3. Content taken from World Health Organization (WHO) and United Nations Children's fund (UNICEF)	1. Nurse delivered awareness sessions<br>2. Lectures; power point presentations<br>3. Live demonstrations (whiteboard, drawings)

**31**	# 21026	Shaikh, Prasidda, and Eruva, 2025	1. Handwashing techniques<br>2. Nail hygiene<br>3. Bathing practices<br>4. Importance of using footwear	Series of interactive health education sessions focusing on hygiene were delivered by a collaborative team of paediatricians and Preventive and Social Medicine (PSM) experts using<br>1. Visual aids<br>2. Demonstrations<br>3. Group discussions<br>4. Storytelling	Series of interactive health education sessions focusing on hygiene were delivered by a collaborative team of paediatricians and PSM experts using<br>1. Visual aids<br>2. Demonstrations<br>3. Group discussions<br>4. Storytelling
