# Supplementary material for: Integration of oral health promotion in primary school health programs: A scoping review
Source: PLOS Glob Public Health. 2026 Oct 5;6(10):e0007390. doi: 10.1371/journal.pgph.0007390 (PMC13638057; doi:10.1371/journal.pgph.0007390)
Supplement: Supplementary file 7 [file pgph.0007390.s007.docx]

**Manuscript title:** Integration of Oral Health Promotion in Primary School Health Programs: A Scoping

**Reviewer’s comments**

This is an important and relevant research topic with significant public health implications, particularly in relation to preventive oral healthcare and school-based health promotion. The manuscript is generally well written and addresses a potentially valuable area of evidence synthesis. However, there are some methodological and reporting concerns that require clarification and strengthening.

1. Kindly clarify the inconsistency in the reported search timeline. The abstract stated that publications from 1981–2025 were included, whereas the methods section reported searches conducted “from inception to February 2026,” with updates in April 2025 and February 2026. Please clearly specify:

- the actual publication date range covered;
- whether “1981–2025” refers only to publication years;
- whether the February 2026 update identified studies published beyond 2025.

2. The eligibility criteria appear insufficiently aligned with the stated objective of evaluating integration of oral health into school health programmes. Several included studies focused primarily on nutrition, immunization, child health, and general hygiene without a clear oral health component. Kindly justify the inclusion of studies that did not explicitly address oral health integration within school health systems or programmes.
3. Consider restructuring the eligibility criteria using the Population–Concept–Context (PCC) framework to improve methodological clarity and consistency.
4. Clearly define the Population included in the review. Specify whether eligible populations included:

- school children/adolescents;
- teachers;
- school health personnel;
- community health workers linked to schools;
- dental professionals involved in school programmes;
- policymakers/programme implementers;
- parents/ caregivers.

5. Clearly define the Concept (“integration of oral health”). Eligible studies should explicitly describe oral health being incorporated into school health programmes, policies, systems, or educational activities. This may include:

- oral hygiene education;
- toothbrushing programmes;
- fluoride education/interventions;
- sugar reduction or diet-related oral health education;
- teacher-led oral health instruction;
- school dental screening;
- fissure sealant or fluoride varnish programmes;
- oral health policies within school health systems;
- integration into school curricula;
- training of teachers or health workers for oral health promotion;
- Implementation outcomes such as feasibility, acceptability, barriers/facilitators, sustainability, and cost-effectiveness.

6. Clearly define the Context and eligible settings. Specify:

- type of schools included (primary, secondary, special, boarding, public/private, community schools);
- level of programme implementation (district/state/national);
- whether policy documents, school health guidelines, or government implementation reports were eligible sources.

7. Kindly consider **revisiting the study selection process** to **ensure that all included publications directly contribute evidence** regarding oral health integration into school health programmes as stated above
