# Supplementary material for: Integration of oral health promotion in primary school health programs: A scoping review
Source: PLOS Glob Public Health. 2026 Oct 5;6(10):e0007390. doi: 10.1371/journal.pgph.0007390 (PMC13638057; doi:10.1371/journal.pgph.0007390)
Supplement: Supplementary file 8 [file pgph.0007390.s009.docx]

**PLOS Global Public Health Decision: Revision required [PGPH-D-26-00971] - [EMID:a6d93d71505702b0]**

PGPH-D-26-00971

Integration of Oral Health Promotion in Primary School Health Programs: A Scoping Review
PLOS Global Public Health

Dear Dr. Surani,

Thank you for submitting your manuscript to PLOS Global Public Health. After careful consideration, we feel that it has merit but does not fully meet PLOS Global Public Health’s publication criteria as it currently stands. Therefore, we invite you to submit a revised version of the manuscript that addresses the points raised during the review process.

The manuscript has been evaluated by four reviewers, and their comments are available below.

The reviewers have raised a number of concerns that need attention. Could you please revise the manuscript to carefully address the concerns raised?

Please submit your revised manuscript by Aug 16 2026 11:59PM. If you will need more time than this to complete your revisions, please reply to this message or contact the journal office at [globalpubhealth@plos.org](mailto:globalpubhealth@plos.org). When you're ready to submit your revision, log on to <https://www.editorialmanager.com/pgph/> and select the 'Submissions Needing Revision' folder to locate your manuscript file.

Please include the following items when submitting your revised manuscript:

- A letter that responds to each point raised by the editor and reviewer(s). You should upload this letter as a separate file labeled 'Response to Reviewers'.
- A marked-up copy of your manuscript that highlights changes made to the original version. You should upload this as a separate file labeled 'Revised Manuscript with Track Changes'.
- An unmarked version of your revised paper without tracked changes. You should upload this as a separate file labeled 'Manuscript'.

Guidelines for resubmitting your figure files are available below the reviewer comments at the end of this letter.

As the corresponding author, your ORCID iD is verified in the submission system and will appear in the published article. PLOS supports the use of ORCID, and we encourage all coauthors to register for an ORCID iD and use it as well. Please encourage your coauthors to verify their ORCID iD within the submission system before final acceptance, as unverified ORCID iDs will not appear in the published article. *Only* the individual author can complete the verification step; PLOS staff *cannot* verify ORCID iDs on behalf of authors.
We look forward to receiving your revised manuscript.

Kind regards,

Johanna Pruller, Ph.D.
PLOS Staff Editor
PLOS Global Public Health

Journal Requirements:

1. Please ensure that you have uploaded your PRISMA flowchart as Figure 1, or else explain why this is not possible. Blank flowcharts can be found here: <http://www.prisma-statement.org/>
2. We ask that a manuscript source file is provided at Revision. Please upload your manuscript file as a .doc, .docx, .rtf or .tex.
3. We note that your Data Availability Statement is currently as follows: "The study is based on a review of published literature. All relevant data are contained within the manuscript and its Supplementary files."

Please confirm at this time whether or not your submission contains all raw data required to replicate the results of your study. Authors must share the “minimal data set” for their submission. PLOS defines the minimal data set to consist of the data required to replicate all study findings reported in the article, as well as related metadata and methods (<https://journals.plos.org/plosone/s/data-availability#loc-minimal-data-set-definition>).
 
 For example, authors should submit the following data:
 
 - The values behind the means, standard deviations and other measures reported;
 - The values used to build graphs;
 - The points extracted from images for analysis.
 
Authors do not need to submit their entire data set if only a portion of the data was used in the reported study.
 
If your submission does not contain these data, please either upload them as Supporting Information files or deposit them to a stable, public repository and provide us with the relevant URLs, DOIs, or accession numbers. For a list of recommended repositories, please see <https://journals.plos.org/plosone/s/recommended-repositories>.
 
If there are ethical or legal restrictions on sharing a de-identified data set, please explain them in detail (e.g., data contain potentially sensitive information, data are owned by a third-party organization, etc.) and who has imposed them (e.g., an ethics committee). Please also provide contact information for a data access committee, ethics committee, or other institutional body to which data requests may be sent. If data are owned by a third party, please indicate how others may request data access.

If the reviewer comments include a recommendation to cite specific previously published works, please review and evaluate these publications to determine whether they are relevant and should be cited. There is no requirement to cite these works unless the editor has indicated otherwise. 

Additional Editor Comments (if provided):

[Note: HTML markup is below. Please do not edit.]

**Reviewers' comments:**

Reviewer's Responses to Questions

Comments to the Author

**1. Does this manuscript meet PLOS Global Public Health’s** [**publication criteria**](https://track.editorialmanager.com/CL0/https:%2F%2Fjournals.plos.org%2Fglobalpublichealth%2Fs%2Fcriteria-for-publication/1/010f019ed926867d-d999cb8a-e730-4654-a320-833e8cefaaff-000000/yOp-1H3LYF5c3ghJYy-jMHTND5Xfr9M_nO9pZTxByJg=258)**? Is the manuscript technically sound, and do the data support the conclusions? The manuscript must describe methodologically and ethically rigorous research with conclusions that are appropriately drawn based on the data presented.**

Reviewer #1: Yes

Reviewer #2: Partly

Reviewer #3: Yes

Reviewer #4: Partly

**2. Has the statistical analysis been performed appropriately and rigorously?**

Reviewer #1: I don't know

Reviewer #2: N/A

Reviewer #3: N/A

Reviewer #4: N/A

**3. Have the authors made all data underlying the findings in their manuscript fully available (please refer to the Data Availability Statement at the start of the manuscript PDF file)?**

The [PLOS Data policy](https://track.editorialmanager.com/CL0/https:%2F%2Fjournals.plos.org%2Fglobalpublichealth%2Fs%2Fdata-availability/1/010f019ed926867d-d999cb8a-e730-4654-a320-833e8cefaaff-000000/cFqJGFXHVX8IIi3K6SWXVo348dm77bTvbzfSCQy2Enw=258) requires authors to make all data underlying the findings described in their manuscript fully available without restriction, with rare exception. The data should be provided as part of the manuscript or its supporting information, or deposited to a public repository. For example, in addition to summary statistics, the data points behind means, medians and variance measures should be available. If there are restrictions on publicly sharing data—e.g. participant privacy or use of data from a third party—those must be specified.

Reviewer #1: Yes

Reviewer #2: Yes

Reviewer #3: Yes

Reviewer #4: Yes

**4. Is the manuscript presented in an intelligible fashion and written in standard English?**

PLOS Global Public Health does not copyedit accepted manuscripts, so the language in submitted articles must be clear, correct, and unambiguous. Any typographical or grammatical errors should be corrected at revision, so please note any specific errors here.

Reviewer #1: Yes

Reviewer #2: Yes

Reviewer #3: Yes

Reviewer #4: Yes

**5. Review Comments to the Author**

Please use the space provided to explain your answers to the questions above. You may also include additional comments for the author, including concerns about dual publication, research ethics, or publication ethics. (Please upload your review as an attachment if it exceeds 20,000 characters)

6. PLOS authors have the option to publish the peer review history of their article ([what does this mean?](https://track.editorialmanager.com/CL0/https:%2F%2Fjournals.plos.org%2Fglobalpublichealth%2Fs%2Feditorial-and-peer-review-process%23loc-peer-review-history/1/010f019ed926867d-d999cb8a-e730-4654-a320-833e8cefaaff-000000/wtORGrMUCvDO4tNfpndtg21XzRqOhzefiWtI3a58wcA=258)). If published, this will include your full peer review and any attached files.

**Do you want your identity to be public for this peer review?** If you choose “no”, your identity will remain anonymous but your review may still be made public.

For information about this choice, including consent withdrawal, please see our [Privacy Policy](https://track.editorialmanager.com/CL0/https:%2F%2Fwww.plos.org%2Fprivacy-policy/1/010f019ed926867d-d999cb8a-e730-4654-a320-833e8cefaaff-000000/Tt65jfHHa1Pl4vOhcfm8sEvMPytEaORBQ-T8mnydewE=258).

Reviewer #1: No

Reviewer #2: No

Reviewer #3: **Yes:** Morteza Banakar

Reviewer #4: No

 [NOTE: If reviewer comments were submitted as an attachment file, they will be attached to this email and accessible via the submission site. Please log into your account, locate the manuscript record, and check for the action link "View Attachments". If this link does not appear, there are no attachment files.]

Figure Resubmissions:

While revising your submission, we strongly recommend that you use PLOS’s NAAS tool (<https://ngplosjournals.pagemajik.ai/artanalysis>) to test your figure files. NAAS can convert your figure files to the TIFF file type and meet basic requirements (such as print size, resolution), or provide you with a report on issues that do not meet our requirements and that NAAS cannot fix.

After uploading your figures to PLOS’s NAAS tool - <https://ngplosjournals.pagemajik.ai/artanalysis>, NAAS will process the files provided and display the results in the "Uploaded Files" section of the page as the processing is complete. If the uploaded figures meet our requirements (or NAAS is able to fix the files to meet our requirements), the figure will be marked as "fixed" above. If NAAS is unable to fix the files, a red "failed" label will appear above. When NAAS has confirmed that the figure files meet our requirements, please download the file via the download option, and include these NAAS processed figure files when submitting your revised manuscript.

**Response to Reviewer #1**

The manuscript is well-written and comprehensive.

**Response:** We thank the reviewer for their feedback. All comments have been addressed and corresponding changes outlined in our response as per track change version of the revised manuscript.

**Comment 1:** Please provide a clear statement of the reasons for excluding articles removed after full-text screening (e.g., in a PRISMA flow diagram or a supplementary table). This is currently missing.

**Response 1:** A supplementary table *(S3 Appendix)* has been added listing exclusion reasons and number of studies excluded for each reason. The PRISMA 2020 flow diagram has also been updated.

**Comment 2:** Risk of bias assessment: The risk of bias for the included studies should be presented, either in a summary table within the main text or as a supplementary file.

**Response 2:** The primary objective of our review was to comprehensively map the breadth of existing literature on the integration of oral health into elementary school health promotion programs, identify the range of implementation strategies employed, and highlight knowledge gaps to inform future research and practice. Consistent with the methodological guidance for scoping reviews, we did not undertake a formal risk of bias assessment. We have clarified this in the Methods section *(page 8, lines 135 to 140)* and acknowledged it as a limitation in the Strengths and Limitations *(page 88, lines 565 to 568).*

**Comment 3**: Checklist scores: The scores or results of the quality assessment checklists, if applicable, must also be reported, preferably in a table or appendix.

**Response 3:** As no formal critical appraisal or quality assessment was conducted, there are no checklist scores to report. We have clarified as stated above.

**Comment 4:** Clarification regarding a missing eligible study "Effectiveness of an integrated model of oral health promoting schools in improving children’s knowledge and the KAP of their parents, Iran". Could you kindly clarify why it was excluded? I ask this only to ensure transparency and consistency in the selection process.

**Response 4:** Eligibility criteria for the review were (1) presence of oral health activities and (2) presence of general health activities, (3) in an elementary school setting. Upon re-examining the article against our predefined eligibility criteria, we determined that it did not meet the inclusion criteria of presence of general health activities. Hence it was excluded. We have shared the reasons for exclusion of excluded articles in a separate supplementary file.

**Response to Reviewer #2**

**Comment:** This is an important and relevant research topic with significant public health implications, particularly in relation to preventive oral healthcare and school-based health promotion. The manuscript is generally well written and addresses a potentially valuable area of evidence synthesis. However, there are some methodological and reporting concerns that require clarification and strengthening.

**Response:** We thank the reviewer for their feedback. All comments have been addressed and corresponding changes outlined in our response as per track change version of the revised manuscript.

**Comment 1:** Kindly clarify the inconsistency in the reported search timeline. The abstract stated that publications from 1981–2025 were included, whereas the methods section reported searches conducted “from inception to February 2026,” with updates in April 2025 and February 2026. Please clearly specify:
the actual publication date range covered;

- whether “1981–2025” refers only to publication years;
- whether the February 2026 update identified studies published beyond 2025.

**Response 1:** Thank you for identifying this and we apologise for the confusion. The initial database search was conducted in October 2023, with an updated search in April 2025 (yielding 30 studies). Prior to submission of our manuscript to PLOS Global Public Health, a final updated database search was conducted in February 2026, in order to capture any newly published eligible studies. During the February 2026 update, only one new study was eligible for inclusion in data extraction *(study #31 in all tables - by Shaikh, Prasidda, and Eruva published in 2025)*. The final studies (n = 31) for which data was extracted and reported in the current manuscript were published between 1981 and 2025. We have clarified this in the abstract *(page 2, lines 25 to 26)* and results *(characteristics of included studies, page 13, line 258)* sections to ensure consistency. Database search was not restricted by geographical location and time i.e. we searched six databases right from inception to the most current February 2026 update.

**Comment 2:** The eligibility criteria appear insufficiently aligned with the stated objective of evaluating integration of oral health into school health programmes. Several included studies focused primarily on nutrition, immunization, child health, and general hygiene without a clear oral health component. Kindly justify the inclusion of studies that did not explicitly address oral health integration within school health systems or programmes.

**Response 2:** We thank the reviewer for this important comment and appreciate the opportunity to clarify our methodology. One of the primary goals of this review was to map how oral health is incorporated into school health programs, recognizing that integration often occurs under broader health promotion initiatives (e.g., nutrition, general hygiene) rather than standalone oral health interventions. To account for this, our review utilized the Common Risk Factor Approach (CRFA) as a theoretical framework. Under this framework, interventions targeting shared behaviours such as sugar consumption or hygiene routines are recognized as integrated health activities, even if the primary study authors did not explicitly label them as "oral health integration."

However, to ensure strict alignment with our review's core focus:

- We re-checked all included studies to verify that they either explicitly described an oral health activity / outcome within the general health promotion activities OR targeted a direct, shared risk factor (e.g., dietary sugar reduction or face / mouth hygiene) with identifiable data relevant to oral health promotion
- Studies that focused solely on unrelated domains (e.g., general immunization) without a direct common risk factor or oral health component were excluded

We have now added an operational definition of “integration” in the Introduction *(page 5, lines 90 to 101)* and clarified these inclusion boundary rules in the Methods section *(page 9, lines 167 to 174)* to enhance transparency.

**Comment 3:** Consider restructuring the eligibility criteria using the Population–Concept–Context (PCC) framework to improve methodological clarity and consistency.

**Response 3:** The eligibility criteria have now been reorganized according to Population–Concept–Context (PCC) framework to improve methodological clarity. The Methods section has been revised accordingly (*page 9, lines 159 to 178*).

**Comment 4:** Clearly define the Population included in the review.

Specify whether eligible populations included:

- school children/adolescents;
- teachers;
- school health personnel;
- community health workers linked to schools;
- dental professionals involved in school programmes;
- policymakers/programme implementers;
- parents/ caregivers

**Response 4:** We have added additional details regarding the population in the Methods section *(page 9, lines 160 to 166)*.

**Comment 5:** Clearly define the Concept ("integration of oral health").

Eligible studies should explicitly describe oral health being incorporated into school health programmes, policies, systems, or educational activities. This may include:

- sugar reduction or diet-related oral health education;
- school dental screening;
- training of teachers or health workers for oral health promotion;
- Implementation outcomes such as feasibility, acceptability, barriers/facilitators, sustainability, and cost-effectiveness.

**Response 5:** We have expanded the Concept to define "integration of oral health" as the incorporation of oral health-related activities into existing school health programs (explicitly or implicitly) through the school curriculum, policies, education, preventive services, or experiential, participatory, and hands-on approaches. The change is reflected in Methods section *(page 9, lines 167 to 174)*. We have also included a clear conceptual definition of integration informed by relevant literature in the Introduction *(page 5, lines 74 to 79 and lines 90 to 101).*

**Comment 6:** Clearly define the Context and eligible settings.

Specify:

- type of schools included (primary, secondary, special, boarding, public/private, community schools);
- level of programme implementation (district/state/national);
- whether policy documents, school health guidelines, or government implementation reports were eligible sources.

**Response 6:** The methods section *(page 9, lines 175 to 178)* has been expanded with details about the context being primary schools across the world – either private or public, with program implementation at school-level. Clarification regarding the exclusion of policy documents, school health guidelines, and government implementation reports have also been specified *(page 10; line 203)*.

**Comment 7:** Kindly consider revisiting the study selection process to ensure that all included publications directly contribute evidence regarding oral health integration into school health programmes as stated above.

**Response 7:** We revisited the study selection process and confirm that all included publications contribute evidence related to integration of oral health within school health programs, as per the revised PCC framework details and conceptual definition of integration added as stated above.

We have made additional revisions to the methods section to improve transparency of evidence selection process *(pages 9 to 12).*

**Response to Reviewer #3:**

Dear Authors,

**Comment:** The manuscript is generally clearly written, though some sections would benefit from tighter phrasing and improved readability.

**Response:** We thank the reviewer for their feedback. All comments have been addressed and corresponding changes outlined in our response as per track change version of the revised manuscript.

**Abstract**

**Comment 1:** The statement “while only three involved parents and educators” appears inconsistent with the program-population details in Table 1 and the narrative on Page 23. Multiple studies explicitly list parents/guardians or teachers as part of the program population or as active participants (e.g., studies 2, 6, 7, 8, 14, 17, and others). Please verify the exact count and clarify what “involved” means (e.g., active engagement in delivery vs. passive receipt of materials). This detail is central to the paper’s argument about limited multi-level integration, so precision matters.

**Response:** Thank you for the detailed review and identifying this inconsistency. We have re-examined all included studies and revised the manuscript to address discrepancies noted by the reviewer as per point below:

- Specifically, we have added details in Methods *(PCC framework section, page 9, lines 160 to 166)* to clearly distinguish between – (1) ‘program’ population i.e. those who were active (children) or passive (parents and families) recipients of the program or those who were engaged in program delivery (school personnel, other professionals, parents, etc.) and (2) ‘study’ population i.e. those who were studied to assess the program and may not have necessarily received or been part of the program.
- We have also modified the results *(Program Population section, pages 28 to 30)* to include studies identified by the reviewer that were inadvertently omitted from the program population. Further, we have added details on the extent of “involvement” of parents, teachers, and others in the programs as recipients or delivery agents. We have also added table 2 *(pages 30 to 32)* to show this distinction.
- To avoid confusion and to maintain conciseness, we have deleted the write-up on ‘study population’, and directed the reader to S4 Appendix for details *(page 28, lines 292 to 303)*.
- The discussion has also been revised accordingly *(pages 84 to 85, lines 476 to 488)*.
- Table 1 in original manuscript has now been moved to Supplementary file *(S4 Appendix Table S4.1)* based on suggestions from other reviewers.

**Comment 2:** The percentages “unhealthy diet (addressed in 70% studies)” and “poor hygiene (about 40%)” should be double-checked against the final count in Table 2 for exactness. “Close to 80%” and “19.4%” are appropriately qualified or precise; consistency in reporting style would help.

**Response:** Thank you for the minute observation. We have re-checked the data in Table 2 *(table 3 in revised submission)* and made changes in the abstract to ensure accurate reporting of numbers (unhealthy diet is reported in 18 studies and poor hygiene in 11 studies). We have also maintained consistency by using numbers through the manuscript, with minimal use of fractions and percentages.

**Comment 3:** Consider whether the abstract could be slightly condensed (it is currently quite dense) while retaining all PRISMA-ScR elements.

**Response:** Thank you for this suggestion. We have revised the abstract to improve readability by condensing several sentences while retaining all key PRISMA-ScR reporting elements and keeping it below the required length of less than 300 words.

**Introduction**

**Comment:** The common-risk-factor approach and the rationale for integration are well explained and appropriately referenced (Fig 1 is helpful). The transition to the knowledge gap on Page 6 is clear.

Minor clarity point: the sentence beginning “Integrating oral health within existing school health promotion programs has several benefits…” (Page 6, lines 88–90) is a bit long and could be split for easier reading.

**Response:** Thank you for the positive feedback. We have revised the sentence beginning “Integrating oral health within existing school health promotion programs has several benefits…” by dividing it into two shorter sentences *(page 7, lines 117 to 120)* to improve readability and made edits throughout the introduction section to improve readability *(pages 4 to 7).*

**Methods**

**Comment:** Excellent transparency: protocol registration, librarian involvement, dual screening/extraction, pilot-tested form, and calibration exercise are all strengths. One suggestion for added rigor: although risk-of-bias/quality appraisal is not mandatory in scoping reviews, a short explicit justification for its omission (e.g., “given the heterogeneity of study designs and the scoping aim of mapping the breadth of the literature rather than synthesizing effect sizes”) would pre-empt reviewer questions and align with the later acknowledgment of heterogeneity as a limitation (Page 64). The four research questions are well-framed using population-concept-context logic.

**Response:** Thank you for the positive comments and helpful suggestions. We have added a statement in the Methods *(page 8, lines 135 to 140)* explaining that a formal risk-of-bias assessment was not undertaken because we conducted a scoping review, which aims to comprehensively map the available evidence rather than evaluate intervention effectiveness.

**Results**

**Comment 1: Study selection and characteristics**

Clear PRISMA flow description. The geographic and methodological distribution is well summarized. Minor: the statement “Seventeen studies were conducted in high-income regions” would benefit from an explicit note on the classification system used (e.g., World Bank categories at the time of analysis), especially since Southeast Asian studies are grouped with high-income regions in the abstract but discussed separately in the text.

**Response:** Thank you for the positive comments and the suggestion to use the World Bank classification**.** We have classified the countries in all 31 included studies based on the World Bank income classification applicable at the time of the analysis. This is reflected in *figure 3 (page 13, lines 267 to 268).* Accordingly, we have modified the abstract and results to reflect this change.

**Comment 2: Tables 1 and 2**

These tables are impressively comprehensive but extremely long (Table 1 spans ~10 pages). Readability suffers in the current format. I strongly recommend moving the full detailed versions to supplementary files and inserting a condensed summary table in the main text (key columns: study ID, country/region/income level, design, program duration, main oral-health component, common risk factors addressed, key outcomes). This would improve flow without losing information.

**Response:** Thank you for this valuable suggestion. We agree that the tables are extensive. Accordingly, we have created a *figure 3 (page 13, lines 267 to 268)* to show the geographical distribution of studies through a map (instead of table).

We have condensed larger tables from the original submission to create three shorter tables in the revised submission – table 1 on study designs *(pages 15 to 16)*, table 2 on program population *(pages 30 to 32)*, and table 3 on key results *(pages 42 to 54)*.

All details in tables 1 to 4 from original submission (including program activities) have been moved to supplementary file *(S4 Appendix)*.

**Comment 3: Program design / frameworks and outcomes**
The finding that no studies used an explicit integration-specific framework (only four used broader behavioural/educational theories) is one of the paper’s most important contributions. It is clearly stated and well-supported.

**Discussion**

**Comment 1:** The synthesis is strong and logically organized. The points about geographic imbalance, quantitative dominance, limited multi-stakeholder engagement, and the absence of integration frameworks are particularly well made and have clear implications.

**Comment 2:** Readability suggestion: several paragraphs are dense (e.g., the long paragraph on Page 61 beginning “Most programs targeted primary school-aged children…” and the paragraph on Page 62 beginning “A striking gap identified…”). Breaking them into shorter sentences or adding brief sub-headings (e.g., “Geographic and Methodological Patterns”, “Scope of Integration and Theoretical Gaps”) would help readers navigate the key messages.

**Response:** We have revised the Discussion by shortening several long paragraphs. Also, as suggested we have divided the Discussion section into the following sub-headings:

- Geographical and Methodological Scope
- Nature of Integration and Theoretical Gaps
- Strengthening School Oral Health Integration
- Strengths and Limitations
- Implications for Research, Policy, and Practice

**Comment 3:** The observation that substance use, stress, and injury are “largely neglected” is important; a short clause noting that even the studies addressing diet/hygiene rarely bundled these other shared determinants would strengthen the point.

**Response:** Thank you for this insight to strengthen the discussion. We have revised the discussion to emphasize that, although diet and hygiene were commonly addressed, programmes rarely incorporated other shared common risk factors such as substance use, stress, and injury, highlighting an important gap in integrated school health programming *(page 85, lines 504 to 509).*

**Strengths and Limitations**

**Comment:** Balanced and appropriately self-critical. The English-language restriction and heterogeneity are fairly acknowledged. One additional limitation worth brief mention: the broad inclusion criteria (studies with an oral-health component or addressing shared common risk factors) may have captured some programs in which oral-health integration was incidental or minimal. A short note on how the authors handled “degree of integration” during screening/extraction would add transparency.

**Response:** Thank you for the positive feedback and important suggestions. We have added this under Strengths and Limitations *(page 88, lines 562 to 565)* and clarified that, to address variations in the degree of integration, we developed an operational definition of integration and applied this consistently during full text screening and data extraction. Studies were included only if they explicitly or implicitly incorporated oral health within broader school health programs according to our predefined eligibility criteria.

**Conclusions**

**Comment:** These sections are clear, actionable, and appropriately cautious for a scoping review. The emphasis on ecological/integration frameworks, multi-stakeholder engagement, system-level and equity outcomes, and greater LMIC attention is well justified by the findings. Minor suggestion: the final sentence of the Conclusions could explicitly echo the common-risk-factor approach introduced in the Introduction to reinforce thematic coherence.

**Response:** We thank the reviewer for the positive feedback and advice on improving thematic consistency. We have revised the concluding sentence to reinforce the importance of integrating oral health into school health programmes through a common risk factor approach *(page 90, lines 599 to 602).*

**Minor / Editorial Points**

**Comment 2:** Figure captions (especially Fig 1 and the PRISMA flow diagram) should be fully self-explanatory so readers do not need to consult the text. In the program-duration paragraph (Page 24), stating approximate fractions (“about one-third”, “close to a quarter”) is fine, but adding the exact counts (e.g., “7 of 21 studies…”) would increase precision. A few very long sentences appear throughout; a light copy-edit for sentence length would further improve flow.

References are current and relevant; no obvious omissions.

**Response:** Thank you for these helpful editorial suggestions. We have revised the figure captions to make them self-contained, added exact counts alongside approximate fractions where appropriate, and edited the manuscript to improve sentence structure and overall readability.

**Response to Reviewer #4**

**Comment:** This manuscript addresses a relevant and timely topic in global public health by exploring the integration of oral health promotion into primary school health programs. The adoption of recognized frameworks for scoping reviews and the implementation of a comprehensive search across multiple databases are important strengths of the study. However, despite this solid methodological foundation, the manuscript presents conceptual and analytical limitations that weaken its scientific contribution in its current form. Below, I outline several points that, if addressed, could substantially strengthen the manuscript.

**Response:** We thank the reviewer for their feedback. All comments have been addressed and corresponding changes outlined in our response as per track change version of the revised manuscript.

**Comment 1:** A central issue relates to the absence of a clear and **operational definition of “integration.”** Throughout the manuscript, this concept appears to encompass multiple dimensions (such as curricular inclusion, common risk factor approaches, and multicomponent interventions) without explicitly clarifying what constitutes integration within the context of this review. To address this, I recommend that the authors provide a clear conceptual definition, preferably grounded in existing literature, and explicitly describe how this concept was applied in the eligibility criteria. Building on this definition, it would be valuable to classify included studies according to levels or types of integration, which could help structure and strengthen the presentation of results.

**Response 1:** We thank the reviewer for this constructive feedback. We agree that establishing a clear operational definition of "integration" is critical for structuring this review.

a. Operational Definition and Application to Eligibility: We have now added a clear conceptual definition of integration in the Introduction *(page 5, lines 74 to 79 and 90 to 101)* grounded in existing school health promotion literature. Specifically, we define integration of oral health into school health *as ‘explicit or implicit incorporation of oral health-related activities into existing school health programs, by addressing shared risk factors common to oral diseases and other health conditions, to create connections and unification between oral health and other health promotion activities.*

In the Methods section *(page 9, lines 167 to 174)*, we clarified how this definition operationalized our eligibility criteria, specifically including programs where oral health activities were either explicitly linked to school health routines or embedded via shared risk factors (e.g., nutrition education, hygiene policies, etc.).

b. Categorization of Included Studies: Regarding the suggestion to classify studies by theoretical "levels" of integration (e.g., vertical vs. horizontal; macro / meso / micro; etc.), we carefully considered this during analysis. However, none of the primary studies utilized formal integration frameworks or terminology. Retrospectively assigning studies to complex deductive frameworks would have required significant subjective interpretation and introduced author bias. Instead, we adopted an inductive coding approach to group studies according to the concrete implementation strategies reported by the original authors. This resulted in 8 distinct strategy categories presented in the Results section *(page 69)*:
i. Student skill-building or empowerment
ii. Teacher-focused strategies
iii. Interest holder involvement
iv. Changes in school structure or policies
v. Other strategies, including whole school approaches
vi. Oral health inclusion in existing curriculum or curriculum restructuring
vii. Professional support
viii. Improving resource access or product availability

We believe this grounded approach provides a more authentic mapping of how integration is practically operationalized in real-world school settings.

**Comment 2:** Relatedly, the **inclusion criteria** appear broad, particularly in allowing studies that address shared risk factors even when oral health is not clearly a central component of the intervention. This may have led to the inclusion of programs that do not truly represent integrated approaches. It would therefore be important for the authors to clarify how they differentiated integrated programs from those that are merely multicomponent or consist of parallel actions. If possible, the results could be revised to explicitly distinguish between these different scenarios.

**Response 2:** We appreciate this valuable suggestion. We have made several changes in the manuscript to clarify the literature-based definition of integration and how it was conceptualized and operationalized for this review.

We acknowledge that none of the included studies explicitly used the term *“integration of oral health”*. Some studies provided a clear description or mention of the oral health-related activities. For studies where this was not explicitly stated, we implied inclusion of oral health based on whether any of the shared risk factors common to oral diseases and other health problems were addressed. The objective of this review was to identify how oral health has been incorporated into existing school health promotion programs, regardless of the terminology used by the authors.

We therefore defined ‘integration of oral health into school health’ as the *“explicit or implicit incorporation of oral health-related activities into existing school health programs addressing common risk factors so as to create connections and unification between oral health and other health promotion activities”.*

Accordingly, this review included studies if they demonstrated this integration through strategies involving student skill building, curriculum restructuring, school policy changes, teacher and family involvement, professional engagement, resource access, or other coordinated whole school approaches rather than describing or assessing oral health as a standalone intervention without any general health component(s).

These clarifications and explanations can be found in the Introduction *(page 5, lines 74 to 79 and lines 90 to 101)* and Methods section *(page 9; lines 167 to 174).*

**Comment 3:** Another point that would benefit from clarification is the **choice of a scoping review design**. Although the manuscript refers to the use of Arksey and O’Malley and PRISMA-ScR, it does not provide an explicit rationale for adopting this approach. I recommend including a brief justification in the Methods section, explaining why a scoping review was the most appropriate design given the study objectives.

**Response 3:** Thank you for this recommendation. We have added a brief rationale on why a scoping review was the most appropriate methodology in the Methods section *(page 8, lines 135 to 140)*.

**Comment 4:** Regarding the **study selection process**, the authors indicate that reasons for exclusion at the full-text stage were recorded; however, these are not reported in the manuscript. To improve transparency and ensure alignment with PRISMA-ScR recommendations, I suggest including a summary of the main reasons for exclusion, either within the PRISMA flow diagram or as a supplementary table (e.g., presenting categories of exclusion and corresponding counts).

**Response 4:** We agree that this improves transparency and have added a supplementary table (S3) summarizing the reasons for exclusion of full-text articles. The PRISMA flow diagram has been revised to direct readers to this supplementary material.

**Comment 5:** From an analytical perspective, the manuscript presents a very detailed description of the included studies, particularly in the tables. However, the synthesis remains predominantly descriptive. To strengthen the contribution of the study, it would be helpful for the authors to further develop the analysis by identifying patterns or grouping similar strategies. One concrete approach would be to organize implementation strategies using an established framework from implementation science, such as the ERIC taxonomy, which would allow for a more structured and comparable categorization.

**Response 5:** We carefully considered applying the ERIC taxonomy. However, many included studies predated the publication of ERIC (2015) and primarily focused on describing intervention activities and outcomes, with limited reporting of implementation processes, evidence-based implementation strategies, or contextual determinants such as barriers and facilitators.

Consequently, retrospective mapping of intervention strategies to specific ERIC strategies would have required considerable interpretation beyond the reported data and could have introduced subjectivity. We identified recurring patterns inductively and grouped similar implementation approaches into overarching categories (named as “implementation strategies” in the manuscript), providing a structured synthesis of the evidence *(table 3, page 42)*.

We have expanded the Discussion *(page 87, lines 536 to 545)* to highlight the value of implementation science frameworks, including ERIC, for improving the planning, reporting, and evaluation of future school-based oral health integration initiatives.

Additionally, the research team is planning to produce a communication paper addressing reporting of oral health interventions in school-based settings using the ERIC framework.

**Comment 6:** In addition, while the discussion highlights several important findings, these are somewhat dispersed throughout the text. I recommend synthesizing these insights in a more structured format—for example, by including a table of recommendations summarizing implications for program design, implementation strategies, and future research priorities. This would enhance the clarity, applicability, and overall impact of the review.

**Response 6:** We have added a summary table *(table 4, page 89)* that synthesizes key recommendations arising from the review.

**Comment 7:** In some parts of the **discussion**, the interpretation of findings appears to imply effectiveness or impact of the interventions. Given that this is a scoping review, including heterogeneous studies and without formal assessment of methodological quality, I suggest revising these sections to ensure that conclusions remain aligned with the descriptive nature of the study and focused on identifying evidence gaps.

**Response 7:** We have carefully revised the Discussion to avoid language implying intervention effectiveness. For example, throughout the manuscript, we emphasize that the review maps existing evidence and implementation approaches rather than evaluating intervention effectiveness or establishing causal relationships. We have also highlighted the absence of formal methodological quality assessment as an inherent limitation of the scoping review methodology.

**Comment 8:** Finally, regarding the **presentation of results, the tables** are highly detailed, reflecting considerable effort in data organization, but this may hinder readability. I recommend simplifying study identification (e.g., using author and year or study ID) and considering moving part of the detailed information to supplementary materials, making the main tables more concise and easier to navigate.

**Response 8:** Based on suggestions from this reviewer and reviewer #3, we have simplified the presentation of results, condensed original tables into three smaller tables. We have moved all detailed tables from original submission to the Supplementary File *(S4. Appendix)*

The three new summary tables are Table 1: Study Designs *(page 15)*, Table 2: Program Population *(page 30)*, and Table 3: Key Results *(page 42)*.

We have used a map to show geographical distribution of the study to break the monotony occurring as a result of the text and table heavy manuscript *(Figure 3, page 13, line 267 to 268)*.

**Overall Comment:** Overall, this study addresses an important topic and has clear potential to contribute to the field. However, it would benefit from revisions aimed at improving conceptual clarity, strengthening the analytical component, and enhancing methodological transparency

**Response:** The manuscript has been substantially revised in response to these comments by clarifying the conceptual definition of integration, adding additional details in the Population–Concept–Context framework, improving methodological transparency, reducing redundant information, simplifying the presentation of results, and modifying the discussion.
