# Supplementary material for: Integration of oral health promotion in primary school health programs: A scoping review
Source: PLOS Glob Public Health. 2026 Oct 5;6(10):e0007390. doi: 10.1371/journal.pgph.0007390 (PMC13638057; doi:10.1371/journal.pgph.0007390)
Supplement: Peer review history

# Peer review: Decision Letter 0

**DOI:** 10.1371/journal.pgph.0007390.r001

**Authors:** Johanna Pruller

18 Jun 2026

PGPH-D-26-00971

Integration of Oral Health Promotion in Primary School Health Programs: A Scoping Review

PLOS Global Public Health

Dear Dr. Surani,

Thank you for submitting your manuscript to PLOS Global Public Health. After careful consideration, we feel that it has merit but does not fully meet PLOS Global Public Health’s publication criteria as it currently stands. Therefore, we invite you to submit a revised version of the manuscript that addresses the points raised during the review process.

The manuscript has been evaluated by four reviewers, and their comments are available below.

The reviewers have raised a number of concerns that need attention. Could you please revise the manuscript to carefully address the concerns raised?

Please submit your revised manuscript by Aug 16 2026 11:59PM. If you will need more time than this to complete your revisions, please reply to this message or contact the journal office at globalpubhealth@plos.org. When you're ready to submit your revision, log on to https://www.editorialmanager.com/pgph/ and select the 'Submissions Needing Revision' folder to locate your manuscript file.

Please include the following items when submitting your revised manuscript:

- A letter that responds to each point raised by the editor and reviewer(s). You should upload this letter as a separate file labeled 'Response to Reviewers'.
- A marked-up copy of your manuscript that highlights changes made to the original version. You should upload this as a separate file labeled 'Revised Manuscript with Track Changes'.
- An unmarked version of your revised paper without tracked changes. You should upload this as a separate file labeled 'Manuscript'.

Guidelines for resubmitting your figure files are available below the reviewer comments at the end of this letter.

As the corresponding author, your ORCID iD is verified in the submission system and will appear in the published article. PLOS supports the use of ORCID, and we encourage all coauthors to register for an ORCID iD and use it as well. Please encourage your coauthors to verify their ORCID iD within the submission system before final acceptance, as unverified ORCID iDs will not appear in the published article. *Only*  the individual author can complete the verification step; PLOS staff *cannot*  verify ORCID iDs on behalf of authors.

We look forward to receiving your revised manuscript.

Kind regards,

Johanna Pruller, Ph.D.

PLOS Staff Editor

PLOS Global Public Health

Journal Requirements:

1. Please ensure that you have uploaded your PRISMA flowchart as Figure 1, or else explain why this is not possible. Blank flowcharts can be found here: http://www.prisma-statement.org/

2. We ask that a manuscript source file is provided at Revision. Please upload your manuscript file as a .doc, .docx, .rtf or .tex.

3. We note that your Data Availability Statement is currently as follows: "The study is based on a review of published literature. All relevant data are contained within the manuscript and its Supplementary files."

Please confirm at this time whether or not your submission contains all raw data required to replicate the results of your study. Authors must share the “minimal data set” for their submission. PLOS defines the minimal data set to consist of the data required to replicate all study findings reported in the article, as well as related metadata and methods (https://journals.plos.org/plosone/s/data-availability#loc-minimal-data-set-definition).

For example, authors should submit the following data:

- The values behind the means, standard deviations and other measures reported;

- The values used to build graphs;

- The points extracted from images for analysis.

Authors do not need to submit their entire data set if only a portion of the data was used in the reported study.

If your submission does not contain these data, please either upload them as Supporting Information files or deposit them to a stable, public repository and provide us with the relevant URLs, DOIs, or accession numbers. For a list of recommended repositories, please see https://journals.plos.org/plosone/s/recommended-repositories.

If there are ethical or legal restrictions on sharing a de-identified data set, please explain them in detail (e.g., data contain potentially sensitive information, data are owned by a third-party organization, etc.) and who has imposed them (e.g., an ethics committee). Please also provide contact information for a data access committee, ethics committee, or other institutional body to which data requests may be sent. If data are owned by a third party, please indicate how others may request data access.

If the reviewer comments include a recommendation to cite specific previously published works, please review and evaluate these publications to determine whether they are relevant and should be cited. There is no requirement to cite these works unless the editor has indicated otherwise.

Additional Editor Comments (if provided):

[Note: HTML markup is below. Please do not edit.]

Reviewers' comments:

Reviewer's Responses to Questions

**Comments to the Author**

1. Does this manuscript meet PLOS Global Public Health’s publication criteria?

Reviewer #1: Yes

Reviewer #2: Partly

Reviewer #3: Yes

Reviewer #4: Partly

**********

2. Has the statistical analysis been performed appropriately and rigorously?

Reviewer #1: I don't know

Reviewer #2: N/A

Reviewer #3: N/A

Reviewer #4: N/A

**********

3. Have the authors made all data underlying the findings in their manuscript fully available (please refer to the Data Availability Statement at the start of the manuscript PDF file)?

The PLOS Data policy

Reviewer #1: Yes

Reviewer #2: Yes

Reviewer #3: Yes

Reviewer #4: Yes

**********

4. Is the manuscript presented in an intelligible fashion and written in standard English?

Reviewer #1: Yes

Reviewer #2: Yes

Reviewer #3: Yes

Reviewer #4: Yes

**********

Reviewer #1: The manuscript is well-written and comprehensive.

Please provide a clear statement of the reasons for excluding articles removed after full-text screening (e.g., in a PRISMA flow diagram or a supplementary table). This is currently missing.

Risk of bias assessment: The risk of bias for the included studies should be presented, either in a summary table within the main text or as a supplementary file.

Checklist scores: The scores or results of the quality assessment checklists if applicable for your checklist must also be reported, preferably in a table or appendix.

Clarification regarding a missing eligible study "Effectiveness of an integrated model of oral health‑promoting schools in improving children’s knowledge and the KAP of their parents, Iran". Could you kindly clarify why it was excluded? I ask this only to ensure transparency and consistency in the selection process.

Thank You,

Reviewer #2: Manuscript title: Integration of Oral Health Promotion in Primary School Health Programs: A Scoping

Reviewer’s comments

This is an important and relevant research topic with significant public health implications, particularly in relation to preventive oral healthcare and school-based health promotion. The manuscript is generally well written and addresses a potentially valuable area of evidence synthesis. However, there are some methodological and reporting concerns that require clarification and strengthening.

1. Kindly clarify the inconsistency in the reported search timeline. The abstract stated that publications from 1981–2025 were included, whereas the methods section reported searches conducted “from inception to February 2026,” with updates in April 2025 and February 2026. Please clearly specify:

• the actual publication date range covered;

• whether “1981–2025” refers only to publication years;

• whether the February 2026 update identified studies published beyond 2025.

2. The eligibility criteria appear insufficiently aligned with the stated objective of evaluating integration of oral health into school health programmes. Several included studies focused primarily on nutrition, immunization, child health, and general hygiene without a clear oral health component. Kindly justify the inclusion of studies that did not explicitly address oral health integration within school health systems or programmes.

3. Consider restructuring the eligibility criteria using the Population–Concept–Context (PCC) framework to improve methodological clarity and consistency.

4. Clearly define the Population included in the review. Specify whether eligible populations included:

• school children/adolescents;

• teachers;

• school health personnel;

• community health workers linked to schools;

• dental professionals involved in school programmes;

• policymakers/programme implementers;

• parents/ caregivers.

5. Clearly define the Concept (“integration of oral health”). Eligible studies should explicitly describe oral health being incorporated into school health programmes, policies, systems, or educational activities. This may include:

• oral hygiene education;

• toothbrushing programmes;

• fluoride education/interventions;

• sugar reduction or diet-related oral health education;

• teacher-led oral health instruction;

• school dental screening;

• fissure sealant or fluoride varnish programmes;

• oral health policies within school health systems;

• integration into school curricula;

• training of teachers or health workers for oral health promotion;

• Implementation outcomes such as feasibility, acceptability, barriers/facilitators, sustainability, and cost-effectiveness.

6. Clearly define the Context and eligible settings. Specify:

• type of schools included (primary, secondary, special, boarding, public/private, community schools);

• level of programme implementation (district/state/national);

• whether policy documents, school health guidelines, or government implementation reports were eligible sources.

7. Kindly consider revisiting the study selection process to ensure that all included publications directly contribute evidence regarding oral health integration into school health programmes as stated above

Reviewer #3: Dear Authors,

The manuscript is generally clearly written, though some sections would benefit from tighter phrasing and improved readability.

Abstract (Page 2)

The statement “while only three involved parents and educators” appears inconsistent with the program-population details in Table 1 and the narrative on Page 23. Multiple studies explicitly list parents/guardians or teachers as part of the program population or as active participants (e.g., studies 2, 6, 7, 8, 14, 17, and others). Please verify the exact count and clarify what “involved” means (e.g., active engagement in delivery vs. passive receipt of materials). This detail is central to the paper’s argument about limited multi-level integration, so precision matters.

The percentages “unhealthy diet (addressed in 70% studies)” and “poor hygiene (about 40%)” should be double-checked against the final count in Table 2 for exactness. “Close to 80%” and “19.4%” are appropriately qualified or precise; consistency in reporting style would help.

Consider whether the abstract could be slightly condensed (it is currently quite dense) while retaining all PRISMA-ScR elements.

Introduction (Pages 4–6)

The common-risk-factor approach and the rationale for integration are well explained and appropriately referenced (Fig 1 is helpful). The transition to the knowledge gap on Page 6 is clear.

Minor clarity point: the sentence beginning “Integrating oral health within existing school health promotion programs has several benefits…” (Page 6, lines 88–90) is a bit long and could be split for easier reading.

Methods (Pages 6–9)

Excellent transparency: protocol registration, librarian involvement, dual screening/extraction, pilot-tested form, and calibration exercise are all strengths.

One suggestion for added rigor: although risk-of-bias/quality appraisal is not mandatory in scoping reviews, a short explicit justification for its omission (e.g., “given the heterogeneity of study designs and the scoping aim of mapping the breadth of the literature rather than synthesizing effect sizes”) would preempt reviewer questions and align with the later acknowledgment of heterogeneity as a limitation (Page 64).

The four research questions are well-framed using population-concept-context logic.

Results

Study selection and characteristics (Pages 9–10)

Clear PRISMA flow description. The geographic and methodological distribution is well summarized.

Minor: the statement “Seventeen studies were conducted in high-income regions” would benefit from an explicit note on the classification system used (e.g., World Bank categories at the time of analysis), especially since Southeast Asian studies are grouped with high-income regions in the abstract but discussed separately in the text.

Tables 1 and 2 (Pages 11–29)

These tables are impressively comprehensive but extremely long (Table 1 spans ~10 pages). Readability suffers in the current format. I strongly recommend moving the full detailed versions to supplementary files and inserting a condensed summary table in the main text (key columns: study ID, country/region/income level, design, program duration, main oral-health component, common risk factors addressed, key outcomes). This would improve flow without losing information.

Program design / frameworks and outcomes (Pages 59–60)

The finding that no studies used an explicit integration-specific framework (only four used broader behavioural/educational theories) is one of the paper’s most important contributions. It is clearly stated and well-supported.

Discussion (Pages 61–63)

The synthesis is strong and logically organized. The points about geographic imbalance, quantitative dominance, limited multi-stakeholder engagement, and the absence of integration frameworks are particularly well made and have clear implications.

Readability suggestion: several paragraphs are dense (e.g., the long paragraph on Page 61 beginning “Most programs targeted primary school-aged children…” and the paragraph on Page 62 beginning “A striking gap identified…”). Breaking them into shorter sentences or adding brief sub-headings (e.g., “Geographic and Methodological Patterns”, “Scope of Integration and Theoretical Gaps”) would help readers navigate the key messages.

The observation that substance use, stress, and injury are “largely neglected” is important; a short clause noting that even the studies addressing diet/hygiene rarely bundled these other shared determinants would strengthen the point.

Strengths and Limitations (Page 64)

Balanced and appropriately self-critical. The English-language restriction and heterogeneity are fairly acknowledged.

One additional limitation worth brief mention: the broad inclusion criteria (studies with an oral-health component or addressing shared common risk factors) may have captured some programs in which oral-health integration was incidental or minimal. A short note on how the authors handled “degree of integration” during screening/extraction would add transparency.

Implications for Research, Policy, and Practice (Pages 64–65) and Conclusions (Page 65)

These sections are clear, actionable, and appropriately cautious for a scoping review. The emphasis on ecological/integration frameworks, multi-stakeholder engagement, system-level and equity outcomes, and greater LMIC attention is well justified by the findings.

Minor suggestion: the final sentence of the Conclusions could explicitly echo the common-risk-factor approach introduced in the Introduction to reinforce thematic coherence.

Minor / Editorial Points

Figure captions (especially Fig 1 and the PRISMA flow diagram) should be fully self-explanatory so readers do not need to consult the text.

In the program-duration paragraph (Page 24), stating approximate fractions (“about one-third”, “close to a quarter”) is fine, but adding the exact counts (e.g., “7 of 21 studies…”) would increase precision.

A few very long sentences appear throughout; a light copy-edit for sentence length would further improve flow.

References are current and relevant; no obvious omissions.

Reviewer #4: This manuscript addresses a relevant and timely topic in global public health by exploring the integration of oral health promotion into primary school health programs. The adoption of recognized frameworks for scoping reviews and the implementation of a comprehensive search across multiple databases are important strengths of the study.

However, despite this solid methodological foundation, the manuscript presents conceptual and analytical limitations that weaken its scientific contribution in its current form. Below, I outline several points that, if addressed, could substantially strengthen the manuscript.

A central issue relates to the absence of a clear and operational definition of “integration.” Throughout the manuscript, this concept appears to encompass multiple dimensions (such as curricular inclusion, common risk factor approaches, and multicomponent interventions) without explicitly clarifying what constitutes integration within the context of this review. To address this, I recommend that the authors provide a clear conceptual definition, preferably grounded in existing literature, and explicitly describe how this concept was applied in the eligibility criteria. Building on this definition, it would be valuable to classify included studies according to levels or types of integration, which could help structure and strengthen the presentation of results.

Relatedly, the inclusion criteria appear broad, particularly in allowing studies that address shared risk factors even when oral health is not clearly a central component of the intervention. This may have led to the inclusion of programs that do not truly represent integrated approaches. It would therefore be important for the authors to clarify how they differentiated integrated programs from those that are merely multicomponent or consist of parallel actions. If possible, the results could be revised to explicitly distinguish between these different scenarios.

Another point that would benefit from clarification is the choice of a scoping review design. Although the manuscript refers to the use of Arksey and O’Malley and PRISMA-ScR, it does not provide an explicit rationale for adopting this approach. I recommend including a brief justification in the Methods section, explaining why a scoping review was the most appropriate design given the study objectives.

Regarding the study selection process, the authors indicate that reasons for exclusion at the full-text stage were recorded; however, these are not reported in the manuscript. To improve transparency and ensure alignment with PRISMA-ScR recommendations, I suggest including a summary of the main reasons for exclusion, either within the PRISMA flow diagram or as a supplementary table (e.g., presenting categories of exclusion and corresponding counts).

From an analytical perspective, the manuscript presents a very detailed description of the included studies, particularly in the tables. However, the synthesis remains predominantly descriptive. To strengthen the contribution of the study, it would be helpful for the authors to further develop the analysis by identifying patterns or grouping similar strategies. One concrete approach would be to organize implementation strategies using an established framework from implementation science, such as the ERIC taxonomy, which would allow for a more structured and comparable categorization.

In addition, while the discussion highlights several important findings, these are somewhat dispersed throughout the text. I recommend synthesizing these insights in a more structured format—for example, by including a table of recommendations summarizing implications for program design, implementation strategies, and future research priorities. This would enhance the clarity, applicability, and overall impact of the review.

In some parts of the discussion, the interpretation of findings appears to imply effectiveness or impact of the interventions. Given that this is a scoping review, including heterogeneous studies and without formal assessment of methodological quality, I suggest revising these sections to ensure that conclusions remain aligned with the descriptive nature of the study and focused on identifying evidence gaps.

Finally, regarding the presentation of results, the tables are highly detailed, reflecting considerable effort in data organization, but this may hinder readability. I recommend simplifying study identification (e.g., using author and year or study ID) and considering moving part of the detailed information to supplementary materials, making the main tables more concise and easier to navigate.

Overall, this study addresses an important topic and has clear potential to contribute to the field. However, it would benefit from revisions aimed at improving conceptual clarity, strengthening the analytical component, and enhancing methodological transparency.

**********

what does this mean?). If published, this will include your full peer review and any attached files.

**Do you want your identity to be public for this peer review?**  If you choose “no”, your identity will remain anonymous but your review may still be made public.

For information about this choice, including consent withdrawal, please see our Privacy Policy

Reviewer #1: No

Reviewer #2: No

Reviewer #3: **Yes:**  Morteza Banakar

Reviewer #4: No

**********

[NOTE: If reviewer comments were submitted as an attachment file, they will be attached to this email and accessible via the submission site. Please log into your account, locate the manuscript record, and check for the action link "View Attachments". If this link does not appear, there are no attachment files.]

Attachment Submitted filename: Reviewers comment.docx

# Author comment: Author response to Decision Letter 1

**DOI:** 10.1371/journal.pgph.0007390.r002

3 Aug 2026

Attachment Submitted filename: PLOS GPH_Response to Reviewers_01.08.2026.docx

# Peer review: Decision Letter 1

**DOI:** 10.1371/journal.pgph.0007390.r003

**Authors:** Julia Robinson

13 Sep 2026

Integration of Oral Health Promotion in Primary School Health Programs: A Scoping Review

PGPH-D-26-00971R1

Dear Surani,

We are pleased to inform you that your manuscript 'Integration of Oral Health Promotion in Primary School Health Programs: A Scoping Review' has been provisionally accepted for publication in PLOS Global Public Health.

Before your manuscript can be formally accepted you will need to complete some formatting changes, which you will receive in a follow up email. A member of our team will be in touch with a set of requests.

Please note that your manuscript will not be scheduled for publication until you have made the required changes, so a swift response is appreciated.

IMPORTANT: The editorial review process is now complete. PLOS will only permit corrections to spelling, formatting or significant scientific errors from this point onwards. Requests for major changes, or any which affect the scientific understanding of your work, will cause delays to the publication date of your manuscript.

If your institution or institutions have a press office, please notify them about your upcoming paper to help maximize its impact. If they'll be preparing press materials, please inform our press team as soon as possible -- no later than 48 hours after receiving the formal acceptance. Your manuscript will remain under strict press embargo until 2 pm Eastern Time on the date of publication. For more information, please contact globalpubhealth@plos.org.

Thank you again for supporting Open Access publishing; we are looking forward to publishing your work in PLOS Global Public Health.

Best regards,

Julia Robinson

Executive Editor

PLOS Global Public Health

***********************************************************

Reviewer Comments (if any, and for reference):

Reviewer's Responses to Questions

**Comments to the Author**

Reviewer #3: All comments have been addressed

**********

publication criteria?

Reviewer #3: Yes

**********

3. Has the statistical analysis been performed appropriately and rigorously?

Reviewer #3: No

**********

4. Have the authors made all data underlying the findings in their manuscript fully available (please refer to the Data Availability Statement at the start of the manuscript PDF file)?

The PLOS Data policy

Reviewer #3: Yes

**********

5. Is the manuscript presented in an intelligible fashion and written in standard English?

Reviewer #3: Yes

**********

Reviewer #3: Dear Authors,

I appreciate the considerable effort made to address the reviewers’ comments. The revision has substantially strengthened the manuscript, particularly regarding methodological transparency, conceptual definition, and presentation of findings.

Best

**********

what does this mean?). If published, this will include your full peer review and any attached files.

**Do you want your identity to be public for this peer review?**  If you choose “no”, your identity will remain anonymous but your review may still be made public.

For information about this choice, including consent withdrawal, please see our Privacy Policy

Reviewer #3: **Yes:**  Morteza Banakar

**********
